# Genomic evidence supports the “long chronology” for the peopling of Sahul

**DOI:** 10.1126/sciadv.ady9493

**Published:** 2025-11-28

**Authors:** Francesca Gandini, Mafalda Almeida, M. George B. Foody, Nano Nagle, Anders Bergström, Anna Olivieri, Simão Rodrigues, Alessandro Fichera, Gonzalo Oteo-Garcia, Antonio Torroni, Alessandro Achilli, William Pomat, Zafarina Zainuddin, Ken Khong Eng, Tarek Shoeib, Teresa Rito, David Bulbeck, Sue O’Connor, Jarosław Bryk, Maria Pala, Michael J. Grant, Ceiridwen J. Edwards, Stephen J. Oppenheimer, Robert J. Mitchell, Pedro A. Soares, Helen Farr, Martin B. Richards

**Affiliations:** ^1^ School of Applied Sciences, University of Huddersfield, Huddersfield, UK.; ^2^ B-Cell Neoplasia Unit and Strategic Research Program on CLL, IRCCS Ospedale San Raffaele, Milan, Italy.; ^3^ Department of Biology, CBMA (Centre of Molecular and Environmental Biology), University of Minho, Braga, Portugal.; ^4^ Department of Biochemistry and Genetics, La Trobe Institute for Molecular Sciences, La Trobe University, Melbourne, Victoria, Australia.; ^5^ School of Biological Sciences, University of East Anglia, Norwich, UK.; ^6^ Dipartimento di Biologia e Biotecnologie, Università di Pavia, Pavia, Italy.; ^7^ Department of Environmental Biology, Sapienza Università di Roma, Rome, Italy.; ^8^ Papua New Guinea Institute of Medical Research, Post Office Box 60, Goroka, Papua New Guinea.; ^9^ Analytical Biochemistry Research Centre (ABrC) and Human Identification/DNA Unit, Universiti Sains Malaysia, 11800 USM Penang, Malaysia.; ^10^ BPP University, London, UK.; ^11^ Department of Forensic Science, Faculty of Biomedical Science, University of Benghazi, Benghazi, Libya.; ^12^ College of Asia & the Pacific, Australian National University, Canberra, Australia.; ^13^ Coastal and Offshore Archaeological Research Services, Ocean and Earth Science, National Oceanography Centre Southampton, University of Southampton Waterfront Campus, European Way, Southampton, UK.; ^14^ School of Anthropology and Museum Ethnography, University of Oxford, Oxford, UK.; ^15^ Centre for Maritime Archaeology, Archaeology, University of Southampton, Southampton, UK.

## Abstract

The timing of the settlement of Sahul—the Pleistocene landmass formed by present-day New Guinea, Australia, and Tasmania that existed until ~9000 years ago (~9 ka)—remains highly contentious. The so-called “long chronology” posits the first main arrivals at ~60 to 65 ka, whereas a “short chronology” proposes 47 to 51 ka. Here, we exhaustively analyze an unprecedentedly large mitogenome dataset (*n* = 2456) encompassing the full range of diversity from the indigenous populations of Australia, New Guinea, and Oceania, including a lineage related to those of New Guinea in an archaeological sample from Wallacea. We assess these lineages in the context of variation from Southeast Asia and a reevaluation of the mitogenome mutation rate, alongside genome-wide and Y-chromosome variation, and archaeological and climatological evidence. In contrast to recent recombinational dating approaches, we find support for the long chronology, suggesting settlement by ~60 ka via at least two distinct routes into Sahul.

## Teaser

Genome data provide support for the first settlement of Australia and New Guinea via at least two routes by 60,000 years ago.

## INTRODUCTION

The first settlement of Sahul involved substantial sea crossings and was, by implication, also the first definite use of watercraft by *Homo sapiens* (*1*–*4*), but when and how this was achieved remains controversial. In recent years, there has been some high-profile archaeological and scientific dating support for a so-called “long chronology” [≥~60,000 years ago (60 ka)] (*5*–*8*), but these have been contested by some archaeologists who prefer a “short chronology” (<~50 ka), often citing genetic evidence to buttress their case (*9*). They have been backed by most genetic researchers, working with both mitochondrial DNA (mtDNA) (*10*–*15*) and Y-chromosome data (*16*), and claim support from inferences concerning archaic *Homo* introgression, on the grounds that it occurred too recently to be consistent with the longer chronology (*17*, *18*).

Some researchers, using whole-genome data, have opted for an earlier arrival, such as ~51 to 72 ka (*19*) or 62 to 75 ka (*20*), but these analyses have been shown to be problematic (*21*). Others have declined to propose a date while attempting to model dispersal routes from Southeast Asia (*22*). Recognizing the limitations of genetic dating, with its reliance on mutation rate estimations, few geneticists have been dogmatic about their inferred chronologies. Up until now, the most comprehensive mitochondrial studies, in particular, have focused mainly on either southern Sahul—Australia/Tasmania (*12*, *14*)—or northern Sahul—New Guinea/Near Oceania (*15*, *23*)—thus limiting their conclusions about dispersal routes into the continent and the settlement of Sahul as a whole, although Nagle *et al.* (*13*), for example, have cautiously suggested two dispersal routes—one more northerly route into what is now New Guinea and one more southerly into what is now Australia—on the basis of mitogenome patterns. The only exception focused on more recent demographic processes, in the Late Glacial and the Late Holocene (*24*). [We define Late Glacial as post-LGM (Last Glacial Maximum, ~29 to 19 ka) but pre-Holocene global warming period, ~19 to 11.7 ka.] The same has been broadly true for genome-wide studies; for example, the recent analyses of Brucato *et al.* (*22*) focused on the north, with insufficient Australian data for them to detect a second southern-route dispersal.

Questions also remain concerning settlement to the east of Sahul. Oceania has usually been considered separately, yet genetic data indicate close links with Sahul (*25*). Archaeologists traditionally carve Oceania into two. Remote Oceania—including Vanuatu, as well as Fiji, Micronesia, and Polynesia—is assumed to have been first settled during the late Holocene by Austronesian speakers, with ancient DNA (aDNA) evidence adduced in support (*26*–*29*). Near Oceania—the Bismarck Archipelago and the Solomon Islands—is instead assumed to have been settled further back in the Pleistocene, but with archaeological dates considerably more recent than for Sahul itself (*30*).

More broadly, settlement of Sahul and Oceania is directly related to debates about the timing and route of the dispersal of *H. sapiens* out of Africa. Recent analyses of complete human genomes at high coverage (*21*, *31*, *32*) overwhelmingly support arguments based on uniparental marker systems for a single dispersal (*33*, *34*), plausibly along the southern coastal route (*1*, *35*, *36*), although the latter remains contentious (*37*). When this occurred is also still debated, with most estimates ranging between 50 and 70 ka (*28*, *31*), but recently claims enlisting both archaeological and genetic support for much earlier dispersals have attracted attention (*38*). On the other hand, recent estimates using recombinational dating, based on introgressed Neanderthal segments in Upper Paleolithic modern humans, imply dates as low as ~40 ka (*39*, *40*). This would imply that fossil and archaeological evidence for early modern humans before that date was due to an earlier wave of dispersal—a radical reevaluation of the evidence, especially given the novelty of the analytical approach.

Although we describe a previously unidentified prehistoric genome here, aDNA recovery from southern Asia and Sahul to date is poor and there are few prehistoric genomes available (*41*–*43*). When direct evidence from aDNA is lacking, the molecular clock, in combination with the nesting relationships within the mtDNA tree, remains an invaluable tool for estimating the timing of human dispersals (*33*). Here, we analyze a substantial mitogenome dataset from the region (*n* = 2456, including 700 new sequences from Sahul and 245 from the western Pacific) that combines a large database from both New Guinea and Australia. Alongside a careful evaluation of alternative mutation rate estimates, this database increases the precision with which we can estimate founder times, lending genetic support to the long chronology and at least two dispersal routes into Sahul. We also draw Oceania together with Sahul, finding that Near Oceania was first settled at around the same time as Sahul, followed by intense ongoing exchange. Last, we examine these data in the context of available genome-wide and Y-chromosome variation from the region, and archaeological and climatological evidence, to reevaluate and reject the hypothesis that modern human out-of-Africa dispersals before ~75 ka contributed substantially to the genetic pool of non-Africans.

## RESULTS

**Fig. 1.**
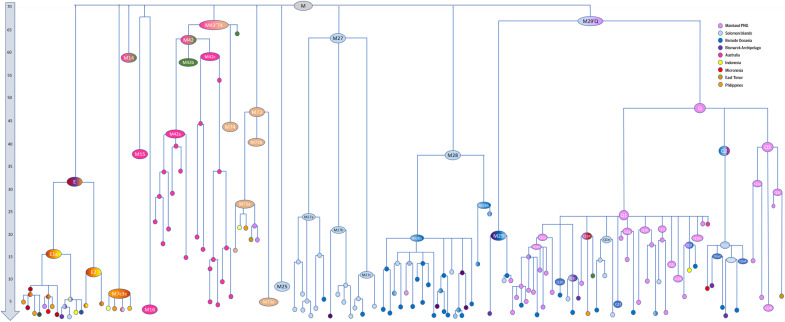
Schematic representation of the maximum-parsimony phylogenetic tree for haplogroup M. The length of the branches is proportional to the coalescence age of the corresponding node. Colors correspond to the geographic origin of the samples encompassed. Timescale is in thousand years ago. Full trees are presented in data S1 (Excel file).

**Fig. 2.**
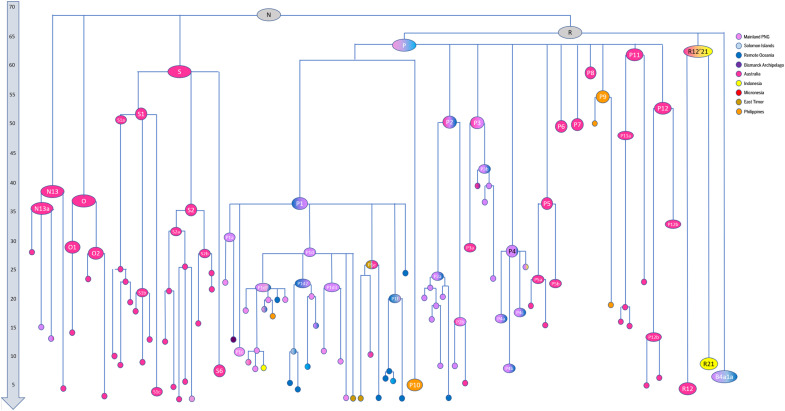
Schematic representation of the maximum-parsimony phylogenetic tree for haplogroup N. The length of the branches is proportional to the coalescence age of the corresponding node. Colors correspond to the geographic origin of the samples encompassed. Timescale is in thousand years ago. Full trees are presented in data S1 (Excel file).

**Fig. 3.**
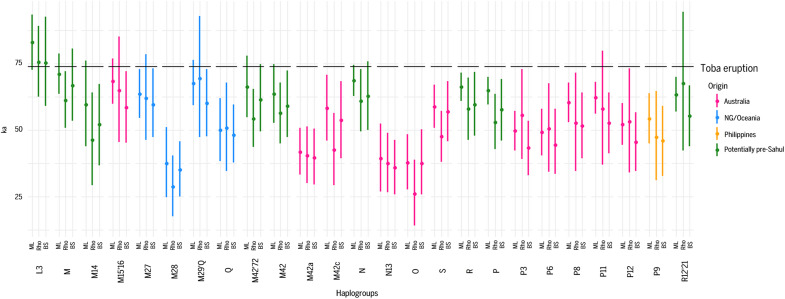
Age ranges (95% CIs) for the major founder lineages in Sahul and their ancestors. We use three different approaches, left to right: ML, ρ, and BI from BEAST with skyline assumption. Data from table S3.

We constructed trees from 2456 mitochondrial genomes (mitogenomes) (Figs. 1 and 2, data S1, and tables S1 and S2) and estimated haplogroup ages using four different methods [maximum likelihood (ML), BEAST skyline, BEAST constant size, and ρ)], including one method (BEAST skyline) with two mutation rates (*33*, *44*) (see table S3). We summarize the results from three methods (ML, BEAST skyline, and ρ) in Fig. 3, which suggests that, while the estimates vary considerably, there is no evidence for any systematic bias. When we cite approximate ages below, the range indicates the overlap between confidence intervals in the three best estimates (*33*).

### The chronology of dispersals from Africa to Sunda

The results include estimates for the age of haplogroup L3, the African ancestor of all non-African mitogenomes. The errors on these estimates overlap in the region of 73 to 89 ka (Fig. 3 and table S3). This value places an upper bound on the timing of the dispersal out of Africa for all modern mitogenomes and robustly precludes any contribution to the modern non-African mitochondrial pool from earlier dispersals before 90 ka, as proposed by some researchers (*38*, *45*).

Lower bounds on the dispersal are provided by the estimates from the three non-African founder haplogroups, M, N, and R—which encompass all ancient non-African mitochondrial variation and are distributed throughout Eurasia and Australasia—as well as some of their informatively distributed subclades. Overall, haplogroup M dates to 67 to 72 ka, haplogroup N dates to 63 to 73 ka, and haplogroup R (which derives from haplogroup N) dates to 61 to 70 ka. Several of their subclades are distributed across South and/or Southeast Asia and Sahul, providing estimates of the age of common ancestry for lineages shared across these regions. Estimates for M42′74 (the existence of which is supported by our new data) and M42, which are distributed across South Asia, Southeast Asia, and Australia, date to 55 to 66 and 53 to 68 ka, respectively. Haplogroup R12′21, a small relict clade that includes both R21 in Mainland Southeast Asia (MSEA) Aboriginal groups and R12 in Aboriginal Australians, dates to 57 to 67 ka. Also within haplogroup R, haplogroup P, which spans Sahul and Aboriginal groups in northern Island Southeast Asia (ISEA), dates rather precisely to 60 to 63 ka. Moreover, Bayesian skyline plots for Eastern New Guinea and Pacific mitogenomes support this picture (fig. S1), with an initial major rapid expansion at 60 to 64 ka.

These age estimates suggest that modern mitogenomes were likely already present outside Africa before 65 ka and reached the Sunda region by ~60 ka, supporting the long chronology for the settlement of Sahul. However, there is a notable geographical disjunction between different mitogenome lineages, both dating to ~60 ka—corresponding roughly, but not entirely, to the phylogenetic distinction between haplogroups M and N—that implies two settlements from distinct sources. They both ultimately trace back to South Asia >60 ka but, more proximally, one entered Sahul from southern Sunda (via MSEA) and one from northern Sunda (via the Philippine archipelago, in ISEA).

### The chronology of the early settlement of northern Sahul

Haplogroup M in Sahul is mainly found to the north, in New Guinea (and Near Oceania), with three major, basal haplogroups diverging from the root of haplogroup M: M27, M28, and M29′Q. Australian clades within M are fewer and less common (*13*): M14, M15′16, M42a, and M42c (Fig. 1, data S1, and table S5).

M29′Q dates to 59 to 73 ka, and diverges into two main clades, M29 (dating to 12 to 21 ka) and Q (dating to 48 to 60 ka). Their restriction to northern Sahul/Near Oceania plausibly suggests an origin within this region. The age of the subclades reflects effective population size and genetic drift rather than the age of the origin of the clusters and are thus minimum ages, with the maximum age indicated by the age of the nesting cluster, M29′Q. Both arose in New Guinea, with recent Holocene dispersals of M29 lineages to the Solomons and Vanuatu. Similarly, whereas Q1 and Q3 are largely restricted to New Guinea (with Q1 moving to Remote Oceania much later), Q2 lineages (35 to 44 ka) are found across Eastern New Guinea, the Bismarcks, and Solomons (and Remote Oceania). Notably, the earliest split within Q2 is between Near Oceania and a single Australian basal Q2 lineage, with Q2a (15 to 21 ka) dispersed across Near Oceania. This overall pattern for both M29 and Q2 again implies a common ancestry in Sahul ~60 ka for lineages in Near Oceania.

On the other hand, while M27a, M27b, and M27c diverged from each other 54 to 73 ka, and coalesce, respectively, ~16 to 26, 15 to 25, and 11 to 15 ka, they are largely restricted to the Solomons. While there is a minor but deep clade in M27b, pointing to a possible LGM origin in New Guinea for this subclade and Late Glacial dispersal to the Solomons, this distribution of M27a and M27c suggests a possible much earlier arrival in Near Oceania. A similar conclusion might be drawn from M25, a small clade largely restricted to the Solomon Islands. Although it lacks diversity (dating to only 5 to 12 ka), it is also basal to haplogroup M, and therefore, given its absence from New Guinea, potentially a relict of a much earlier dispersal east. Earlier arrivals (by ~45 ka for the Bismarcks and ~35 ka for the Solomons) are attested by archaeological evidence (*30*); the mitogenome patterns suggest that the earlier settlers may have been augmented by Late Glacial expansions from New Guinea of M29′Q and M28.

Within haplogroup N’s major subclade, haplogroup R, the most important ancient Sahul founder is haplogroup P, which dates to 60 to 63 ka and comprises 12 basal (or probably basal) subclades. Two of these, P9 (45 to 59 ka) and P10 (2 to 9 ka), are restricted to Philippine Aboriginal groups. Although their age estimates are lower than those of several Sahul subclades, their diversity has likely been reduced by drift—very severely in the case of P10—reflecting the small population size of these Philippine Aboriginal groups. This wide distribution may nevertheless suggest that the overall age of haplogroup P predates its presence in Sahul and might indicate the timing of its arrival from mainland eastern Asia. P1 dates to 25 to 31 ka and is seen mainly in New Guinea, as is P4 (20 to 29 ka). Conversely, P5, P6, P7, P8, P11, and P12 are all Australian and, except for P5 (27 to 39 ka), all date to at least ~50 ka.

P2, dating to 40 to 51 ka, is slightly more complex. P2a (19 to 31 ka) is mainly restricted to New Guinea, with a minor young subclade found in Vanuatu, but the two subclades of P2b are partitioned between New Guinea (7 to 12 ka) and Australia (0.5 to 6 ka)—overall suggesting a recent movement from New Guinea to Australia. Similarly, within P3 (42 to 53 ka), P3a (22 to 32 ka) and P3b1 (20 to 30 ka) are mainly restricted to Australia, but P3b2 (33 to 44 ka) is from New Guinea. This suggests the reverse process, northward from Australia to New Guinea, but much earlier than the recent movement southward of P2b.

### The chronology of the early settlement of southern Sahul

By contrast with New Guinea, which includes diverse lineages belonging to both haplogroups M and R, almost 45% of the mitogenome lineages in Aboriginal Australians are basal to haplogroup N—mainly haplogroups S and O (table S5). Aside from the ubiquitous haplogroup P, the minor R12 is the only important haplogroup R subclade. As mentioned above, haplogroup R12′21 is a minor clade dating to ~56 ka, which includes both R21, found widely in Aboriginal groups in MSEA (*36*, *46*, *47*), and R12, found in several Aboriginal Australians, supporting a southern route into Sahul.

Haplogroups S (51 to 57 ka) and O (28 to 39 ka) are both wholly restricted to Australia, apart from a single basal subclade of S2a2 (dated to 16 to 27 ka), which migrated to New Guinea from Australia around the LGM/Late Glacial or later. Their almost complete absence from both New Guinea and Near Oceania (despite the large volume of data now available) is in notable contrast to the distribution of haplogroup P. This disparity is despite, for haplogroup S at least, being extremely ancient and indeed close to the age of haplogroup P. We cannot entirely rule out that haplogroup S and O lineages were present earlier in northern Sahul and simply diversified after the separation of Australian and New Guinean lineages, or even that they were lost by drift from New Guinean ancestral lineages during the separation process. Nevertheless, the simplest explanation for this pattern suggests that, like R12, they too may be candidates for a distinct, southern route migration into Sahul.

Aside from P, haplogroup S is the next most predominant Aboriginal Australian haplogroup (at 26% in our sample), as well as one of the most diverse. It divides into two main clades, S1 (43 to 58 ka) and S2 (26 to 45 ka), plus the minor S6 (4 to 14 ka) and S3, S4 and S5. The deeper S1 lineages appear to be more northern than the derived subclades, and the “orphan” S3 and S4 lineages are also from the north. We must emphasize that the present distribution of Aboriginal Australians often does not reflect their ancestral homelands; nevertheless, the Papuan S2a2 subclade indicates that S2 may also have been present in the north by around the LGM, lending additional support to this hypothesis.

Haplogroup O separates into O1 (20 to 38 ka) and O2 (21 to 30 ka). In both cases, basal clades/samples are mainly found in northern Australia whereas the more derived ones are found in the south, suggesting ancient dispersals from north to south for this haplogroup, as well as haplogroup S [cf. (*14*)]. Along with the smaller haplogroup N13 (27 to 46 ka), S and O comprise the only basal haplogroup N lineages indigenous to Sahul.

The minor Australian clades belonging to haplogroup M include M14, M15′16, M42a, and M42c. Both the very rare M14 and M15′16 are reconstructed as basal to haplogroup M. M14 dates to 44 to 64 ka; of the mitogenomes available from this haplogroup, one is Australian and one is from Saudi Arabia. They diverge very early, and it may be that this is a trace of the southern route from Africa via South Asia to Australia, but the data are too sparse to draw firm conclusions. M15′16 dates to 60 to 72 ka and includes four Australian individuals, two in each haplogroup, linked only by a single fast-mutating control-region position; M15 and M16 are therefore likely separate basal haplogroup M clades that arrived with the first settlers but, again, have drifted toward extinction over time.

The putative haplogroup M42′74—as per PhyloTree (*48*), Build 17—is shared between South Asia, Southeast Asia, and Australia, but not New Guinea, and would date to 55 to 66 ka. It has long been considered a possible trace of the southern dispersal route but, despite being the most parsimonious reconstruction, has also been sometimes dismissed (*13*) due to the existence of the clade unifying M42 and M74 depending on a single, rather fast-evolving position. However, the putative clade now includes a third basal subclade, seen in a single lineage from India, lending additional weight to the reconstruction.

M42a and M42c are both entirely restricted to and widespread in Aboriginal Australians (making up almost 20% of our sample), whereas their non-Sahul sister clade, M42b, is almost entirely restricted to India (*49*). M42a and M42c date to 33 to 50 ka and 46 to 56 ka, respectively, and M42b dates to 47 to 59 ka; under this reconstruction, the three subclades are basal and M42 itself dates to 53 to 68 ka, placing an upper bound on the divergence of the three subclades. Meanwhile, M74, if it can indeed be considered part of the picture, dates to 33 to 49 ka and has evolved largely in MSEA, with more recent dispersals (since the LGM) both to ISEA and India. If it were genuinely part of the same phylogenetic grouping, it would add weight to the suggestion that M42′74 is a marker of not only the southern coastal route out of Africa (*35*, *36*) but also a southern route ~60 ka into Sahul itself.

### Northern and southern routes into Sahul

In conclusion, we can heuristically attribute the ancestral lineages of M25, M27, M28, and M29′Q (because of their northern distribution) and haplogroup P (because of its links to northern ISEA), plus the single New Guinean orphan lineage R14 (*11*) to a northern route of entry into Sahul. We can attribute the ancestral lineages of M42a, M42c, and R12 (because of their links to South Asia/MSEA); S, O, M14, M15, and M16 (because of their uniquely southern distribution); and N13 (because of the nesting of northern New Guinean within southern Australian lineages) to a southern route. On these grounds, only the intrusive Late Glacial/postglacial lineages such as haplogroups E, M7c3c, M73a2, and some N13 and S2 lineages in New Guinea (7% of the total sample) and haplogroup Q lineages in Australia (3% of the total) are excluded from the Pleistocene founders.

**Fig. 4.**
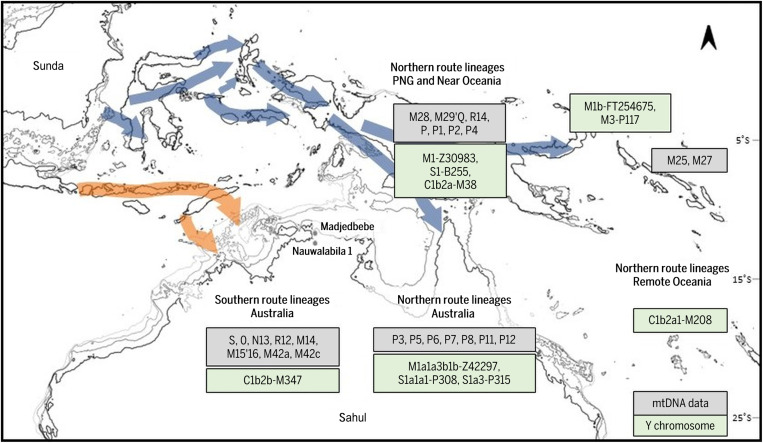
Map showing continental shelves of Sunda, Sahul, and the Western Pacific. Coastal contours at 0, −50, −80, and −120 m below sea level indicate the changing extent of landmass with fluctuating sea levels. Potential migration routes are indicated and the likely marker lineages for mtDNA and Y chromosome are labeled. ESRI, ArcGIS, GEBCO Compilation Group (2020) GEBCO 2020 Grid (doi: 10.5285/a29c5465-b138-234d-e053-6c86abc040b9)

If we make these assumptions (Fig. 4 and table S5)—with the strong caveat that our samples are unlikely to be wholly representative of variation in the present-day regions, let alone the distributions of lineages in the distant past—we can heuristically estimate that (i) 100% of nonintrusive lineages from New Guinea, the Bismarcks, and the Solomons derive from the northern route and (ii) ~36% of nonintrusive lineages in Australia also result from the northern entry, with the remaining 64% deriving from ancestors who took the southern route. Overall, then, most of the extant lineages in ancient Sahul and Near Oceania descend from ancestors who arrived via the northern route, by way of the northern part of the now submerged Sunda continent and northern Wallacea (*46*), at ~60 ka. However, a minority of lineages overall (but around two-thirds of those in Australia) arrived via a southern route, through southern Sunda.

### Multiple postglacial dispersals east and west

Our highly resolved phylogeographic portrait also reveals a complex mosaic of Late Glacial and postglacial movements between Southeast Asia, Sahul, and the Pacific (see the Supplementary Materials for details). These involved not only lineages from haplogroups E and M7c from ISEA, during the Holocene (*46*, *50*), but also P4 from Eastern New Guinea in the reverse direction, and M73 from MSEA in the Late Glacial. M73a2, dating to 15 to 23 ka, is found within the eastern New Guinea highlands, with a sister lineage in Timor and nesting lineages in ISEA. These observations suggest movements eastward into Sahul at the time that Late Glacial sea-level rise disrupted the Sunda continent.

Haplogroup Q spread widely from its New Guinea homeland after the LGM. The evidence for Late Glacial (Q2a4, dating to 10 to 16 ka) and more recent dispersals further east perhaps reflects the intensification of exchange seen in the archaeological record, for example, in the emergence of obsidian exchange networks from the Terminal Pleistocene onward (*51*, *52*). Haplogroup Q1l (9 to 21 ka) dispersed both east and west during the late Holocene: While one subclade is seen in the eastern New Guinea highlands and north coast, another small subclade is seen in Vanuatu, and a distinct lineage is seen in the aDNA sample reported here from Gua Talimbue, Sulawesi, radiocarbon dated to the Iron Age, 256 to 393 cal. AD (1710 ± 20 BP: SANU 40418) (*53*). Notably, this individual completely lacked New Guinean ancestry in the rest of her genome (fig. S8). In this respect, she is unique among other reported ancient Sulawesi genomes, of which two are much more recent and one pre-Neolithic (*41*, *42*)—but all of which lack New Guinean mtDNAs.

While there are few members of haplogroup Q seen in modern Wallacea in our dataset, a putative nesting clade encompassing Q1j (6 to 18 ka), Q1k (6 to 17 ka), and Q1l (the former being largely restricted to New Guinea) is also seen in individuals from both southern Borneo and the Malay Peninsula. Haplogroup Q3 (30 to 39 ka), which is otherwise restricted to New Guinea, also includes a small basal subclade of Q3c (20 to 35 ka) present in both Timor and the Malay Peninsula. Haplogroup Q1 lineages further afield mainly reflect the role of New Guinea in the spread of the Lapita culture and Austronesian languages, but they also spread as far north as Taiwan (*54*) and even to Madagascar (*55*). One is seen in an Aboriginal Australian with Torres Strait (and therefore likely ultimately New Guinean) ancestry. The same explanation probably applies to the Q1* lineages seen in two Aboriginal Australians from northern Queensland, dating to roughly the last 100 years (*12*).

### Testing the mtDNA picture with Y-chromosomal evidence: Chronology

There are substantial published Y-chromosome SNP data available from both New Guinea and Australia that have not been either fully analyzed or integrated into other datasets. We therefore took advantage of these data to explore the extent to which they might support or challenge the models presented here. We constructed a Y-chromosome SNP tree from the Eastern New Guinea data of Bergström *et al.* (*23*) (fig. S2), to complement the geographic profile and whole Y-chromosome tree presented there, as well as the whole Y-chromosome trees presented in Bergström *et al.* (*16*) and Haber *et al.* (*56*). We conclude that—contrary to the way it has often been interpreted—the Y-chromosome evidence is consistent with both the model of the long chronology and with at least two routes from Sunda into Sahul.

First, we consider the out-of-Africa dispersal itself. The situation differs slightly from that for mtDNA, where there was a single non-African founder lineage, L3 (*36*, *57*). For the Y chromosome, it has been commonly assumed either (i) that just one Y-chromosome founder lineage, CT-M168, left Africa (*34*), followed by back-migrations of haplogroup E-M96; or (ii) that there were three founders, haplogroups D-M174, C-M130, and F-M89, which have a common ancestor within Africa dating to ~77 ka (*34*, *56*), with the latter two dating to ~53 to 54 ka (*56*). Although it is now clear, from the existence of the minor D0 haplogroup, that D-M174, and therefore DE-M145, arose within Africa, with non-African lineages diverging from ~71 (63 to 81) ka, Haber *et al.* (*56*) also consider but then dismiss a third option, that the founders were C-M130, DE-M145, and F-M89, with haplogroups D0 and E-M96 migrating back into Africa.

However, a fourth model, in which DE-M145 diverged within Africa, and CF-P143 diverged outside Africa, also fits the evidence. In this case, the major founders would be DxD0, which separated from D0 ~71 (63 to 81) ka and CF-P143, which separated from DE-M145 ~76 (65 to 86) ka (*56*). If we accept that this can explain the current Y-chromosome phylogeography, so that haplogroups C and F diverged outside Africa, the evidence is clearly compatible with an out-of-Africa dispersal before ~65 ka, in agreement with the mtDNA evidence.

There are three major Y-chromosome haplogroups in the indigenous populations of Sahul, within C, S, and M (*16*). Note that these Sahul clusters are each highly derived within the overall non-African part of the Y-chromosome tree, because they differ in this respect from the female lineages (which are mostly basal to the three non-African M, N, and R haplogroups). It has sometimes been argued that the ancestors of Sahul populations were distinct from the main wave of out-of-Africa dispersal (*20*, *31*) but, as for mtDNA, the Y-chromosome data clearly connect Sahul to other East Eurasian populations, in line with more recent autosomal results (*21*).

Because of this tight nesting structure, the ages of the Sahul-specific clusters are heavily (and very usefully) constrained by the ages of nesting clades, so that the Sahul/Oceania-specific C1b2-B477 lineages are nested by C1b-F1370, and sibling to C1b1-K281 (which fall into distinct subclades in South Asia and Southeast Asia). Similarly, the age of K2b1-P399, which nests S-B254 (aka K2b1a) and M-P256 (aka K2b1b) (ISOGG 2019: https://isogg.org/tree/index.html), is constrained by the age of K2b-M1221, which also nests haplogroup P-P295 (aka K2b2), which (in the forms of haplogroups R and Q) is widespread in Eurasia and pre-Columbian America. Therefore, these ages provide valuable upper bounds for the settlement of Sahul.

For the upper bounds, using the Bayesian calibration of Fu *et al.* (*58*), Bergström *et al.* (*16*) obtained an age of 54.1 (47.8 to 61.4) ka for C1b-F1370 and 54.3 (48.0 to 61.6) ka for K2b-M1221. Their estimates for the Sahul-specific lineages themselves (lower bounds for the age of settlement) are 53.3 (47.1 to 60.5) ka*.* These ages may themselves be underestimates, as discussed below, but even if this were not the case, although they concluded that settlement most likely occurred at ~50 ka (*16*), their age ranges are clearly compatible with the long chronology, with initial settlement at ~60 ka.

### Testing the mtDNA picture with Y-chromosomal evidence: Dispersal routes

Y-chromosome phylogeographic distributions do not map precisely onto mtDNA distributions, but there is nevertheless distinctive patterning. Early branching lineages of Y-chromosome haplogroup S-B254 are seen in Indonesia and the Philippines (S3-P336 and S4-BY22870), including the Aboriginal Aeta people of Luzon (S2-B273), as with mtDNA haplogroup P. S1a-Z41335 lineages are interleaved between north and south, with the northern lineages shared across Wallacea, New Guinea, and Near Oceania, again calling to mind the distribution of mtDNA haplogroup P and suggesting a northern route into Sahul. The sister clade of Y-chromosome haplogroup S, haplogroup M-P256, by contrast, is largely restricted to the north, with signs of a recent Holocene southern dispersal into Torres Strait islanders (*16*). M2-M353 and M3-P117 are in New Guinea and the Bismarcks, respectively, and M1-Z30983 is mainly in New Guinea, with the rare M1b-FT254675 seen in Wallacea and the Bismarcks. Aboriginal Australians belong to the highly derived M1a3b1b-Z42297 subclade. Its widespread but northern distribution resembles that of the mtDNA M29′Q lineages, again pointing toward a northern-route ancestry.

The third major founding Y-chromosome lineage, haplogroup C1b2-B477, is a sibling clade to C1b1-K281, which has distinct subclades in South Asia and MSEA, possibly indicating a southern route into Sahul. However, unlike the inferred southern-route founder lineages in the mtDNA, C1b2-B477 is Sahul-wide, with C1b2b-M347 restricted to Aboriginal Australians and C1b2a-M38 widespread across the Philippines, Wallacea, New Guinea, and the Bismarck Archipelago, and it also disperses, as the derived C1b2a1-M208, further east into Remote Oceania (*59*, *60*). The Holocene history of C1b2a-M38 is similar in some respects (but see below) to that of the so-called mtDNA “Polynesian motif” (B4a1a1a) (*61*), with the age of C1b2a1-M208 rather similar at ~9.4 ka (YFull). However, it has a much deeper ancestry within the western Pacific, splitting from its sibling Aboriginal Australian subclade, C1b2b-M347, around the time of first settlement.

Thus, while there is no absolute one-to-one overlap of mtDNA and Y-chromosome founder lineages, likely reflecting the different impact of genetic drift on male and female lineages during the early settlement—as is well documented for more recent settlement processes (*34*)—the Y-chromosome evidence can nevertheless be regarded as consistent with the dual-source model.

### Testing the mtDNA picture with genome-wide evidence

We carried out PCA (principal component analysis) and ADMIXTURE analyses on published genome-wide data from across Southeast Asia and the Pacific (table S4). It is worth mentioning at this point that clustering analyses such as these rarely assign unique clusters appropriately to small, divergent samples in a dataset. Rather, they tend to emerge as a mixture of other, better represented populations. For this reason, we ran two sets of analyses to counter this effect. We focus mainly on the first analysis but augment it with details from the second where differences in the splits driven by sample size affected the outcome. We also analyzed the Aboriginal Australian samples with a European and Indian dataset (fig. S5), to check the impact of recent European admixture, and we also ran PCAs with the two datasets, for comparison (figs. S6 and S7). The datasets we used are displayed in table S4 and fig. S11.

**Fig. 5.**
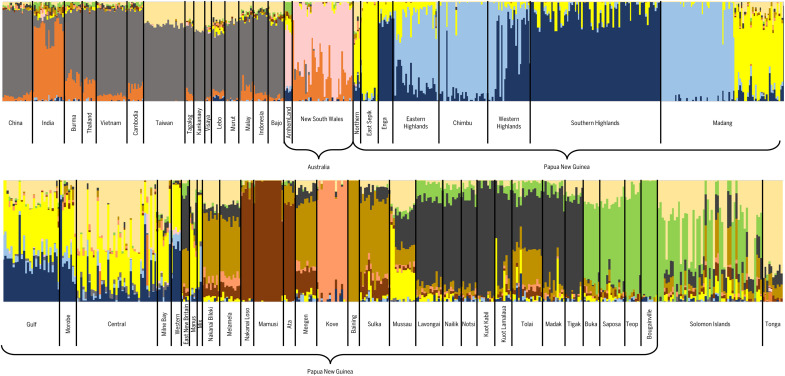
ADMIXTURE analysis 1 plot. This analysis includes more Aboriginal Australians, compared to analysis 2 (see table S5) at *K* = 12, using 37,216 SNPs. *K =* 2 to *K =* 15 are shown in table S4.

We labeled the two sets of analyses as “analysis 1” (including more Aboriginal Australians: Fig. 5 and fig. S3) and “analysis 2” (including more from ISEA and Pacific islands: fig. S4). The two ADMIXTURE analyses gave broadly similar results, with the first differing mainly by resolving the Aboriginal Australians more clearly, and the second by resolving the Polynesians more clearly, due to there being more of each population in the respective datasets.

With due caution, we can interpret the order of the earlier splits, at least, as a relative chronological succession. In this light, as described in detail in the Supplementary Materials, the most notable feature of the ADMIXTURE analysis is the order of the earliest splits within Sahul/Oceania. Following the first split, between Sahul/Oceania and East Eurasia, the next separates New Guinea from the islands of Near Oceania (the Bismarcks and Solomons), followed by the next (in analysis 1) separating Aboriginal Australians (with a component partly shared with Indians: see the Supplementary Materials). The next is within Near Oceania, with New Britain on one side and New Ireland plus the Solomons on the other, and the next is within New Guinea itself. This is followed by a split within the Eurasian fraction that separates an “Austronesian” component, which disperses across ISEA and the western Pacific, including coastal parts of New Guinea, and further splits subdivide regions within Near Oceania.

We also included in this analysis our Iron Age sample from Sulawesi, which carries the Papuan mitochondrial Q1l lineage, alongside 17 further genomes from ancient Wallacea, including a pre-Neolithic sample from Sulawesi (*41*, *42*) (fig. S8). Notably, despite its clear Papuan mtDNA ancestry, the Iron Age sample was the only archaeological sample from Wallacea to carry no discernible Oceanian autosomal ancestry. This result is confirmed by PCA (fig. S9). This indicates both that ancestry from New Guinea, while ancient (*41*), was not ubiquitous in prehistoric Wallacea, but possibly (at least, since the spread of the Neolithic) restricted to coastal regions, and that sufficient time had elapsed for the ancestry represented by the maternal lineage to have been “washed out” of the autosomal genome in this individual.

## DISCUSSION

Since the 1950s, with the advent of radiometric and trapped-charge dating, Australia and New Guinea have swung markedly from being regarded as one of the most recently settled parts of the world to one of the most ancient outside Africa, even if the precise timing has been difficult to establish. This has had major implications for the region’s understanding of itself and its place in the world, with political and social ramifications that continue to unfold (*62*). Archaeogenetics has played an important role in this process by demonstrating that, almost uniquely outside Africa, most of the ancestry of the modern Aboriginal people of Australia and New Guinea can be traced to the earliest settlement. In terms of uniparental markers, there is a suite of mtDNA and Y-chromosome lineages that are unique to the continent, which evolved from clearly defined ancestors within the continent, so that their ages provide lower bounds for their time of arrival. This means that a sufficiently comprehensive analysis can contribute substantially to the ongoing debate on the chronology of settlement.

In this study, we have sequenced 973 mitogenomes from across contemporary Australia, New Guinea, Oceania, and Southeast Asia, and analyzed them together with 1483 published mitogenomes from the region. Whereas in some parts of the world, notably Europe, aDNA has become the most important strand of genetic evidence for mapping dispersals and settlement, this is not yet the case for Sahul. In our study, the only ancient “Sahulian” mitogenome we were able to retrieve was a Q1 lineage from Sulawesi dating back to only ~1.7 ka, curiously alongside an autosomal genome lacking any Sahulian ancestry. While informative about subsequent dispersals, such results cannot tell us much about the earliest stages of settlement of the continent. We must therefore rely largely upon inferences from contemporary variation, but, as we show here, with sufficient data and care with the molecular clock analyses, these can be highly informative.

### Archaeological estimates for first arrivals in Sunda and Sahul 

Radiometric and trapped-charge dates from Sunda and Wallacea are few. Currently, the oldest firm dates for Sunda are 63 to 73 ka from Sumatra, based on U-series and ESR dates for modern human teeth, and TL dates from elsewhere in the cave (*6*); and 67 to 73 ka based on combined U-series–ESR of a modern human cranial fragment at Tam Pà Ling in Laos. Other dates from Borneo and the Malay Peninsula indicate ages >40 ka with no clear upper bound; the same is true for Wallacea, with minimum occupation ages of ~43 ka for Timor and ~53.5 ka for Sulawesi. For New Guinea, there are similarly few dates: Occupation of the Huon Peninsula northeastern highlands dates to at least 40 ka, and possibly 47 to 61 ka, with ages of 40 to 48 ka for the southeastern highlands.

There are more data from Australia, with the oldest known sites located in the Northern Territory. Roberts and colleagues combined OSL (optically stimulated luminescence) and radiocarbon to estimate an occupation age of 53 to 60 ka at Nauwalabila I. More recently, Clarkson *et al.* (*5*) estimated an age of 53 to 65 ka at Madjedbebe. The lowest occupation horizon is bracketed by multiple OSL dates, replicated across four excavated sections, but there are signs of vertical displacement of artifacts, and an age range of 53 to 60 ka, similar to Nauwalabila I, is likely. A third rock shelter from the Northern Territory, Nwarla Gabarnmang, has a similar archaeological record and a proposed age of 46 to 52 ka, but as the radiocarbon dates do not extend to the base of the artifact-bearing layers, the age of first occupation must be older than 47 to 49 ka and thus potentially contemporary with those of Madjedbebe or Nauwalabila I, in the 53 to 60 ka range. The other early dated site is an open-air deposit from Minjiwarra, suggesting earliest occupation at 47 to 51 ka.

In Western Australia, numerous sites are dated to 45 to 51 ka, but some of these dates used radiocarbon and could therefore be minimum ages. In South Australia, the rock shelter sequence at Warratyi, based on the presence of pigments, has been dated by OSL and radiocarbon to ~49 ka, with artifacts only appearing after 40 ka. In New South Wales, the Lake Mungo burials represent the oldest human skeletal remains in Australia, but the earlier ESR estimate of ~63 ka has been revised using OSL to ~40 ka, with occupation bracketed by OSL to 45 to 50 ka. For details and references, see the Supplementary Materials.

### Reevaluating the mtDNA mutation rate

The mutation rate used for human mitogenome evolution is critical to testing between the long and short chronologies. We have estimated ages with two main approaches: the Soares *et al.* (*33*) rate that includes an explicit correction for the known impact of purifying selection, and the Fu *et al.* (*44*) rate that draws upon Bayesian inference and radiocarbon-dated ancient mitogenomes. When the two are compared (see table S3) it is clear that, while for a range of distinct methodologies the Soares rate supports the long chronology, the Fu rate estimates using BEAST and assuming sudden demographic expansion are more consistent with the short chronology—although note that these assumptions and methodology deliberately minimize the ages, and that other assumptions and methodologies, even with the Fu rate, raise the estimates into the long chronology range. Researchers have equivocated on which rate to apply (*12*), with some favoring the latter (*14*, *15*), so it is critical to understand better whether there are sound grounds to prefer one over the other.

The Bayesian approach has some problems that have received little attention. In practice, it gives extremely variable outputs, such that any given rate is hard to replicate. The outcome depends on precisely which samples are included in the calibration; even with similar sets of samples, the estimated rate can be very different, and biases appear toward the ages of the predominant samples used in the calibration. Moreover, in the cases of ages in the range 40 to 80 ka, there are few calibration points. The oldest in Fu *et al.* (*44*) is from Tianyuan, in northern China, dating to 39.5 ka. In addition to age bias, there is also a profound geographical and phylogenetic bias: Only 2 out of 12 samples used were non-European, and only Tianyuan belongs to haplogroup N. Moreover, one sample (the Iceman) was inadvertently uncalibrated, reducing its age by >12%.

Although subsequent calibration attempts have been made with more samples, these have exacerbated the biases. For example, in Posth *et al.* (*63*), all 35 samples were European, and while 12 were 20 to 30 ka, none exceeded 30 ka. Only three belonged to haplogroup M (and those to a single, extinct subclade of M), and the comparable estimates seem to be younger still, with an out-of-Africa dispersal at ~44 to 55 ka (*63*).

Furthermore, the divergence times estimated depend very much on the assumed demographic model; while the rapid growth model substantially reduces the estimates compared to those obtained from the Soares rate, the constant-size model elevates it [table 1 in (*63*); see also our table S3]. In practice, of course, the history of these populations is an extremely complex mixture of both models and other factors. Last, we note that the Fu and similar rates, analyzed with the growth assumption in BEAST, are incompatible with the most recent scientific dating for the settlement of Sunda and Sahul, which indicate that *H. sapiens* had arrived by at least ~50 to 60 ka [(*5*) etc.; see above]. Assuming that these settlers were part of the same dispersal as all mitogenomes in people alive today, the ancestry of haplogroups M and N should extend back at least 60,000 years, as the Soares rate suggests. The latter is also authenticated at the near end of the scale (*61*) (see the Supplementary Materials), and the lack of a selection correction in the Fu rate combined with the lack of suitable calibration points and resulting biases suggests strongly that applying the Soares rate is more reliable at higher time depths and, therefore, more suitable for dating the settlement of Eurasia and Sahul.

### Support for the long versus short chronology

Results using the method of recombination dating have been used to propose a very recent split time for non-Africans and, by implication, a chronology for Sahul that is little more than 40,000 years (*39*, *40*). These analyses would require a wholesale revision of the way the archaeological dates were interpreted and imply that numerous modern human remains and material culture between 60 and 40 ka were the products of people who left no descendants. Our results suggest that such a radical conclusion may be premature.

Our new analyses suggest, rather, that the main dispersals of *H. sapiens* out of Africa took place between 73 to 89 ka (our estimated age range for the ancestral African haplogroup L3) and 63 to 72 ka, the age range for haplogroups M and N. If we combine the age ranges for M and N, we can narrow the latter estimate to 67 to 69 ka. This would be our best estimate of the emergence of the non-African lineages from the L3 ancestors in the vicinity of the Gulf, soon after leaving East Africa. This date range (*31*) supports the suggestion that the out-of-Africa dispersals took place in the wake of the “nuclear winter” triggered by the Mount Toba eruption in Sumatra, ~74 ka, as previously suggested by analyses of South Asian data (*35*, *36*), and not before the eruption, as proposed by some (*38*, *45*).

Despite this renewed support for a relatively recent out-of-Africa dispersal, our age estimates for the arrival in Sahul are substantially earlier than those of the short chronology, whose proponents argue for an arrival after ~50 ka. These estimates rather indicate that modern mitogenomes likely reached the Sunda region by ~60 ka, supporting the long chronology for the settlement of Sahul following a relatively rapid dispersal from Africa.

On the other hand, as suggested above, this outcome is consistent with the balance of the radiometric and luminescence dating results for both Sunda and Sahul. In Sunda, although there are few early dates, the oldest confirmed sites are Lida Ajer in Sumatra, dating to 63 to 73 ka (*6*) and Tam Pà Ling in Laos, at 67 to 73 ka (*7*). Unlike the interpretation of the latter authors, our results would encompass these remains within the main out-of-Africa dispersal, while suggesting that the somewhat earlier tibia fragment, at 68 to 86 ka and possibly older, might not be anatomically modern, as the authors assume.

In Sahul, the oldest ages are from the Northern Territory in Australia, which date to 53 to 60 ka (or, less conservatively, 53 to 65 ka) (*5*), and with dates widespread in the habitable areas of the continent, in a wide variety of environments, by 45 to 50 ka (*30*). This suggests a potential arrival during the glacial low stands of Marine Isotope Stage (MIS) 4, 57 to 71 ka, and expansions within Sahul following the onset of warming in MIS 3 (*64*).

Refining this picture, O’Connor and Hiscock (*30*) argued that the most likely window for arrival was 62 to 59 ka, given that seasonal winds from the direction of Timor (via the southern “Banda Arc” island chain) would have been favorable before ~58 ka, when there is a potential reduction in the northwest monsoon. The rising sea levels at the time (rather than the low stand often assumed to be more favorable) would then have encouraged the development of more sophisticated watercraft. Kuijjer *et al.* (*3*) meanwhile argued that, given both the reduced distance and decrease in tidal strength during the low stand, crossings may have been easier at 65 ka than at 50 ka. A time window of 59 to 65 ka appears to fit very well with a synthesis of the climatological, archaeological, and the genetic chronologies presented here (Fig. 3)—although, given our estimate of 60 to 63 ka of mtDNA haplogroup P (which nests Sahulian lineages and thus provides an upper bound on settlement time), we regard an arrival much earlier than ~60 ka as very unlikely.

### At least two dispersal routes into Sahul

The Pleistocene dispersal into Sahul, via the island world of Wallacea, involved several days of voyaging, potentially crossing ~100 km of open water (*4*, *65*), but—like the timing—the route or routes taken by the early voyagers have been intensively debated for decades without having been resolved. Ancient Sundaland, now mainly submerged due to modern sea-levels, has long been considered the most likely source for dispersal and has recently received support from genome-wide analyses (*42*). The debate about dispersal routes from Sunda into Sahul goes back to Birdsell (*66*), who proposed a northern route to New Guinea via Sulawesi and the Moluccas, and a southern route to Australia via Flores and Timor (Fig. 4). The southern route was favored by Birdsell himself, as well as by some recent modelers (*67*) (who also propose a secondary northern entry), whereas others have considered the northern route more likely (*4*, *68*). Recent reconstructions of likely island-to-island intervisibility during the glacial maximum of MIS4 at 60 to 70 ka, suggest that both routes into Sahul were plausible at this time (*69*, *70*). Simulation work has estimated the number of founders (in the hundreds) and suggested that the dispersal was very likely both deliberate and rapid (*4*).

Nevertheless, reconstructing dispersal routes from genetic data has proved challenging (*12*, *15*). This is partly because, as part of an almost continuous process of dispersal from Eastern Africa to Oceania (*35*, *67*), the divergence between Sunda and Sahul was both ancient and rapid. This led to mitogenome lineages sharing very few variants in common that can now provide the basis for phylogenetic nesting analyses. This contrasts with the Americas, for example, where tens of thousands of years separated the first settlers in northeast Asia and the dispersals into the New World, and nesting relationships among uniparental lineages are very clear (*71*).

However, many analyses have also suffered from lack of a sufficiently comprehensive dataset encompassing the whole of Sahul. For example, Brucato *et al.* (*22*) recently used *f3* and *f4* statistics and qpGraph analyses of genome-wide data to propose that settlement was via the northern route but lacked Australian data that might have allowed them to detect a second southern-route dispersal. Moreover, the interpretation of genome-wide analyses, which lack the polarity imposed by gene-tree analyses of the uniparental markers, remains very problematic. For example, their proposal that ADMIXTURE and TREEMIX analyses indicate the earliest settlement in southeast New Guinea overlooked the fact that mitogenome analyses have suggested that extant patterns most likely represent traces of more recent, post-LGM dispersals in the opposite direction (*15*, *72*).

The size of our dataset and strong representation of both New Guinean and Australian lineages allow us to tentatively disentangle this long-running question. First, we reemphasize the point that it is clear from the mitogenome data that New Guinean and Australian ancestry diverged at a very early stage in the settlement process (*12*). This is contrary to a suggestion based on whole genome sequences (*31*), where the reduced divergence was most likely an effect of later gene flow, for which we see distinct signals in the mtDNA data.

Our data show that the three Eurasian mtDNA founders—haplogroups M, N, and R—are well represented in Sahul, but with profoundly different geographic distributions. Haplogroup M includes two-thirds of lineages in northern Sahul, with some of its oldest lineages found in the Solomon Islands and the Bismarck Archipelago, and these probably represent the first settlers arriving in northern Sahul from northern Sunda, ~60 ka.

An ancient separation of northern Sahul (New Guinea and Near Oceania) from southern Sahul may also be supported by the order of splitting in the genome-wide clustering analyses (although see the Supplementary Materials). The north coast of New Guinea lies on the edge of the Australian tectonic plate, is mostly steep, and was likely extremely forbidding to settlement during the late Pleistocene (*73*), although more archaeological fieldwork is needed to test this hypothesis. In terms of migrations, however, while a rapid coast-hugging route around the north of the continent of Sahul has not, to our knowledge, been suggested in the past, these results suggest that some of the earliest northern-route pioneers may indeed have spread around the northern coast and rapidly onward into the Bismarcks and Solomons. This may explain the extremely ancient split we see in both the mtDNA and genome-wide data.

By contrast, haplogroup N in Sahul encompasses 80% of Australian sequences. The major clade in both regions, haplogroup P (nested within R, and R within N), was evidently introduced via the northern route into Sahul but dispersed very early on, and further than the main haplogroup M lineages, into Australia and Tasmania (*74*). However, the uniquely Australian haplogroup N lineages are all basal to N, except for the extremely minor R12 lineage. This indeed indicates a phylogenetic patterning to the north–south division within the three major founders—with M and R predominating in the north (and across Sahul as a whole), but N predominating in the south.

The northern route—possibly originating from several distinct geographic sources, given the distinct patterning of P and M—therefore seems to have been the predominant influence across Sahul. It is even conceivable that the proposed southern route may simply be an artifact of genetic drift among southern lineages. We consider this unlikely, however, given the higher drift before 25 ka in the north than the south (which we infer based on fewer coalescences in the tree: Figs. 1 to 3).

The largest distinct Australian lineage is haplogroup S, which encompasses two widespread major derived subclades, alongside four minor derived subclades with long basal branches, reflecting long-term isolation and small population sizes after the first arrivals. Papuan subclades nested within Australian clades, like S2a2 (and N13a), show that people living in northern Australia migrated northward as well as southward after the LGM, but the lack of any sign of an ancient presence in the north suggests a distinct southern route into Sahul. Evidence for the peopling of northern Australia from southern Sunda (via Timor), followed by later migrations toward the southern and western part of the country (*14*), is also supported by the phylogeography of haplogroup O, with basal clades mostly in the north and derived clades in the south and west.

These conclusions remain tentative, because we have allocated many of the indigenous Sahul lineages to one route or the other based largely on their current distribution, rather than on the much clearer indications that would be given by phylogenetic nesting patterns (where a subclade localized to a sink region is nested within a clade otherwise localized in a clear source). However, there are two markers whose nesting patterns do delineate the two routes more clearly.

First, haplogroup P—present at similar levels of 30 to 40% in Aboriginal Australians (excluding Eurasian lineages) and Papua New Guineans—exhibits a likely ancestral trail, signaled by basal lineages present especially in the Aboriginal Aeta people from central Luzon, but also elsewhere in the Philippines at low levels (*75*, *76*), suggesting ancestry somewhere along the northern route (*15*, *75*). The connection of Sahul populations to Aboriginal Philippine groups is reinforced by the presence of distinctively high levels of Denisovan-related archaic-related introgression in both, possibly relating to interbreeding with *Homo luzonensis*, in the Philippines, and *Homo floresiensis*, in Wallacea, or even within Sahul itself (*17*, *77*, *78*). Second, our support for the existence of the clade M42′74 further substantiates both a southern route out of Africa (with M42a and M42c restricted to Australia and M42b restricted to India) (*35*, *36*) and a route to Sahul through MSEA (where M74 is found), lending weight to a southern dispersal.

Thus, the mitogenome evidence reported here further shifts the balance of the debate toward the likelihood of at least two distinct dispersal routes into Sahul, both northern and southern, with a common ancestry in Sundaland, supporting separate analyses of primarily Australian (*12*) and New Guinean (*15*) lineages. Although it may be impossible to pinpoint the precise landfall points with genetic data, modeling of exposed MIS4 island chains suggests that the likely arrival points were the Bird’s Head peninsula of West New Guinea and the northwest Sahul shelf (*3*, *70*, *79*). We suggest, on grounds of the very early splits and divergences within Near Oceania, that the earliest arrivals were in the Bird’s Head, with the northern-route settlers further splitting there to spread both north and south.

Using genealogical information to reconstruct pre-European contact phylogeographic patterns for lineages sampled in the early 20th century, Tobler *et al.* (*14*) proposed a detailed coastal colonization model for Australia, involving haplogroups P4b1, P5, S, and M42a moving south around the east coast and haplogroup O traveling south around the west coast. Although this model does not partition the Australian lineages in quite the same way as our dual entry model, we do not feel that this compromises either suggestion, given the likely complexity of the early dispersal processes. In the future, whole-genome sequence data from the region should allow us to test these models and to elaborate them in much greater detail.

Our analyses, stemming from a large new dataset analyzed alongside our published data and a critical evaluation and deployment of the mtDNA molecular clock, support the long chronology for the settlement of Sahul, plausibly via at least two dispersal routes, one from ISEA (northern Sunda/Asian mainland) and one from MSEA (southern Sunda), with genetically distinct groups of migrants both arriving in Sahul at a similar time, ~60 ka. Both routes trace back to South Asia and ultimately to Eastern Africa, ~75 ka. This timescale is contrary to recent estimates based on recombinational dating for the out-of-Africa dispersal after 50 ka but meshes well with the fossil and archaeological record. We have addressed and refined a Western science narrative that supports the peopling of Sahul in deep time but acknowledges and respects the ontological perspective that many Indigenous people hold: “We have always been here” (*62*).

## MATERIALS AND METHODS

### Sampling

DNA samples from Eastern New Guinea, Papua New Guinea (PNG) were collected with individual-level consent by S. Oppenheimer and G. Koki, and are under the custodianship of W. Pomat and M. Laman: see Bergström *et al.* (*23*). Samples from Vanuatu were originally collected with individual-level consent in multiple local substudies and updated approval for sample use has been received from the Vanuatu Cultural Centre. These, and other samples from the Solomon Islands, Philippines, Indonesia, and Brunei were collected as part of multiple collaborative projects performed between 1980 and 2000 with individual-level consent, coordinated by J. Clegg and others, and now archived as part of the Oceanian Genome Variation Project (OGVP), with approval for use by the Oxford Tropical Research Ethics Committee. All OGVP samples are now fully anonymized with the exception of island location of sampling. Samples from Australia were collected with individual named consent by R. J. Mitchell and L. Williams: see Nagle *et al.* (*12*, *13*). Previously unpublished University of Huddersfield mitogenome data from Southeast Asia include Malaysian samples collected by Zafarina Zainuddin and Ken Khong Eng, with individual named consent approved by Human Research Ethics Committee, Universiti Sains Malaysia and the University of Leeds Faculty of Biological Sciences Research Ethics Committee. DNA sequencing was approved by the Ethics Committees of the University of Leeds, Faculty of Biological Sciences (Southeast Asian samples) and University of Huddersfield, School of Applied Sciences (Sahul and Pacific samples).

We used published genome-wide data spanning China to the Pacific (summarized in table S4). If not already done, the data were converted to PLINK format to render them suitable for the analyses performed here. We used Affy2vcf bcftools plugin to convert Australian samples to variant call format (VCF) and then to PLINK. Published Y-chromosome SNP data were from Bergström *et al.* (*23*).

### Mitogenome sequencing 

We amplified mitogenomes in two long-range PCR fragments following a modified version of the published protocol. We designed a new reverse primer for the second fragment (6345 REV: 5′-AGATGGTTAGGTCTACGGAGGC-3′) to overcome poor performance in some PCR reactions. We also halved the volumes of the Go*Taq* LongPCR Master Mix (Promega), which allowed us to double the number of reactions we could perform with each kit. Some of the DNA samples were of poor quality and we therefore amplified shorter fragments from these, splitting each long-range original fragment with two internal primers to obtain four overlapping amplicons. We used 10,175 REV (5′-GCACTCGTAAGGGGTGGAT-3′) and 9815 FOR (5′-CCACGGACTTCACGTCATTA-3′) for fragment 1 (now 1a and 1b) and 1677 REV (5′-GTTTAGCTCAGAGCGGTCAAGT-3′) and 1404 FOR (5′-ACTTAAGGGTCGAAGGTGGATT-3′) for fragment 2 (now 2a and 2b).

We then processed the amplicons to obtain FASTQ and FASTA files. We manually checked all doubtful positions, as well as heteroplasmies, with Geneious 6.1; we consider a position heteroplasmic when each allele’s frequency is between 31 and 70%. After quality checks, we obtained a total of 1158 complete mitogenome sequences: 78 from Australia, 728 from Eastern New Guinea in PNG, 28 from the Solomon Islands, and 324 from Vanuatu. After haplogroup classification, we excluded 213 not relevant to the current research (either haplogroup B or European lineages). The total dataset analyzed included 973 new sequences: 635 from PNG, 65 from Australia, 16 from the Bismarcks and Solomons, and 229 from Vanuatu, and we also included 28 from MSEA and ISEA (table S1). For details of references, see the Supplementary Materials.

### Phylogenetic analyses and molecular clock dating

A preliminary haplogroup classification with Haplogrep 2 was confirmed manually following the classification of PhyloTree Build 17 (*48*) and later papers updating the phylogeny (*13*). To build the most up-to-date version of the phylogeny, we searched the literature (i) for available mitogenomes from Sahul and (ii) for all the published mitochondrial sequences belonging to any lineage with more than three samples from Sahul. We excluded from this search haplogroup B because it has been already extensively studied (*61*) and falls outside of the scope of this study, as it was not involved in the first peopling of Sahul. For the analyses, we divided the collected sequences into the two macro-haplogroups M and N, encompassing a total of 1699 and 757 mitogenomes, respectively (table S1).

We built maximum-parsimony trees with the aid of mtPhyl using the rCRS as a reference sequence. A new haplogroup label was assigned following the established nomenclature only when the candidate haplogroup encompassed at least five samples and two haplotypes and was not defined by a control region mutation alone. We disregarded the unreliable indels at nps 309, 315, 515 to 522, and 16,193 and hotspots at nps 16,182, 16,183, and 16,519 when identifying clades and excluded them from the trees.

We used ModelGenerator v0.85 to select the optimal nucleotide substitution model, which, according to the Bayesian information criterion (BIC) test, is TN93 + I + G6. We estimated coalescence times using ML and Bayesian approaches, as well as the ρ (rho) statistics (average distance of the haplotypes of a clade from the respective root haplotype) accompanied by a heuristic estimate of the standard error (σ) calculated from an estimate of the genealogy. We used PAML v4.9j to obtain ML estimates, assuming the TN93 mutation model with γ-distributed rates (approximated by a discrete distribution with 32 categories) and two partitions: coding region (from nps 577 to 16,023) and control region (from nps 16,024 to 576). We performed these calculations considering all substitutions except those at np 16,519 and the 16,182C and 16,183C. We converted mutational distances (both ML and ρ) into years using the substitution rate of about one mutation every 3624 years for the entire mitogenome and correcting for purifying selection using the calculator provided by Soares *et al.* (*33*). We used BEAST v1.10.4 for the Bayesian age estimates and the Bayesian skyline plots. We ran the analyses under the TN93 substitution model (gamma-distributed rates plus invariant sites) with a strict clock and a Bayesian skyline tree model. The chain length was established at 50,000,000 iterations, with samples drawn every 5000 Markov chain Monte Carlo steps, after a discarded burn-in of 5,000,000 steps.

### Genome-wide analyses

We retrieved genome-wide data from the literature comprising nearly 100 populations from Southeast Asia and the Pacific (table S4). We note that the genome-wide data from Malaspinas *et al.* (*31*) have not been made publicly available and, thus, could not be included. We downloaded data in VCF files and converted them to a binary pedigree (BED) format with VCFtools software. We used PLINK1.9 to filter the data, ensuring that all samples had the same number of SNPs. We excluded individuals with more than 10% missing genotypes and excluded SNPs with MAF (minor allele frequency) <0.01 and with a 5% missing genotype rate. We also excluded markers that failed the Hardy-Weinberg test at the default threshold. The files resulting from the extraction process comprised 1368 individuals, and we converted them into standard pedigree format (PED) to perform ADMIXTURE, sNMF, and PCA. After filtering, we used ADMIXTURE 1.23 software to cluster the observed genotypes into *K* ancestral proportions, with all parameters set to default and the values of *K* between 2 and 15. The software also calculated CV errors for each value of *K*. We also performed PCA using PLINK1.9, set to output 10 PC vectors, to display relations between individual genomes.

## Supplementary Materials

### The PDF file includes:

Supplementary Text Figs. S1 to S10 Tables S4 to S6 Legends for data S1 and tables S1 to S3 References
