# Supplementary material for: Genomic evidence supports the “long chronology” for the peopling of Sahul
Source: Sci Adv. 2025 Nov 28;11(48):eady9493. doi: 10.1126/sciadv.ady9493 (PMC12662211; doi:10.1126/sciadv.ady9493)
Supplement: Supplementary file 1 — Supplementary Text Figs. S1 to S10 Tables S4 to S6 Legends for data S1 and tables S1 to S3 References [file sciadv.ady9493_sm.pdf]

Science Advances
AAAS

Supplementary Materials for

Genomic evidence supports the "long chronology" for the peopling of Sahul

Francesca Gandini *et al.*

Corresponding author: Helen Farr, r.h.farr@soton.ac.uk; Martin B. Richards, m.b.richards@hud.ac.uk

Sci. Adv. **11**, eady9493 (2025)
DOI: 10.1126/sciadv.ady9493

The PDF file includes:

Supplementary Text
Figs. S1 to S10
Tables S4 to S6
Legends for data S1 and tables S1 to S3
References

Other Supplementary Material for this manuscript includes the following:

Data S1 and tables S1 to S3

Details of Methodology references

DNA amplification protocol (*82*); FASTQ and FASTA data processing (*83*); haplogroup classification with Haplogrep 2 (*84*); use of mtPhyl using the rCRS as reference sequence (*85*); use of ModelGenerator v0.85 (*86*) to select optimal substitution model; ρ (rho) dating (*87*) and standard error (σ) (*88*); PAML v4.9j (*89*) for ML estimates; BEAST v1.10.4 (*90*) for Bayesian age estimates and skyline plots; VCF files converted to BED format with VCFtools software (*91*); ADMIXTURE 1.23 (*92*) used to estimate ancestral proportions and EIGENSOFT for PCA (*93*).

DNA analysis from ancient remains

The findings of an early attempt to extract aDNA from an ancient site in Australia, whilst still discussed today, have been dismissed by further analyses (*94*). In a comprehensive reanalysis, Heupink et al. (*95*) have clearly shown the results to be due to a combination of contamination and artefacts, likely generated by mitochondrial nuclear inserts, and that the only genuine sequences recovered were from a late Holocene individual belonging to haplogroup S2. Other "ancient" Australian sequences date only from the past few hundred years.

We sampled two individuals from Sulawesi: FG005, a molar from pre-Neolithic (dated contextually to 1500–8000 BC) Gua Mo'o hono, and FG006, a mandible radiocarbon dated to Iron Age Gua Talimbue (1710 ± 20 BP: SANU 40418) (*53*). We carried out the laboratory preparation at the Ancient DNA Facility in the University of Huddersfield under clean-room conditions. This facility has positive air pressure and is physically separated from all other molecular biology labs dealing with modern sources of DNA. Laboratory researchers wore hairnets, face masks, full body suits and two pairs of gloves throughout the drilling, extraction, and library preparation processes. We frequently cleaned all tools and surfaces with bleach and LookOut® DNA Erase (Sigma-Aldrich), as well as through exposure to UV light. Prior to processing, we photographed the samples and then decontaminated their surfaces by UV radiation for 30 minutes on each side, followed by cleaning with 5μm aluminium oxide powder using a compressed air abrasive system. Using a diamond-tipped circular saw, we cut the root from the tooth and excised a portion from the mandible. We obtained bone powder by crushing these sections in a Mixer Mill (Retsch MM400) for 30 seconds at 30Hz/second. We extracted DNA from ~150 mg of the powder (*96*) with modifications (*97*). We included blank controls throughout the extractions, library preparation and amplification reactions to monitor for possible modern DNA contamination.

We constructed next-generation sequencing libraries from the aDNA extracts using published methods (*98*), with modifications (*83*). We quantified all amplification reactions using a Qubit® ds-DNA High Sensitivity assay kit on a Qubit® 3.0 Fluorometer and additionally checked the quality of each library using an Agilent 2100 Bioanalyzer High Sensitivity DNA kit, prior to pooling equimolarly for next-generation sequencing purposes. We screened the samples on an Illumina MiSeq in the Bradley Lab at Trinity College Dublin to determine the endogenous DNA content. For the sample with the higher endogenous content (FG005, at 0.40%), we sent three dual-indexed libraries for 100-bp (base-pair) single-end sequencing on a Hi-Seq4000 (Macrogen, South Korea), but we obtained no useful data. We therefore also prepared double-stranded UDG-treated libraries, which were enriched at the Reich Lab, Harvard Medical School, through capture of 1240K SNPs via in-solution probes (*99*).

Although the pre-Neolithic FG005 was classified as male and could be seen to include the variant at np 489, suggesting mtDNA haplogroup M, no other variants were scorable, and the damage assessment suggested contamination (confirmed by PCA). We were therefore unable to extract useful data from this sample. The first millennium FG006, which had only 0.12% endogenous DNA, appeared authentic, based on DNA damage patterns. It was classified as female and yielded data from 45,000 genome-wide SNPs and a meaningful mitogenome sequence: a novel basal sequence within haplogroup Q1l with private transitions at nps 739, 8844, 11113, 15638 and 16154 (discussed further below, as well as in the main text).

Provenance. The Gua Mo'o hono remains were excavated in 2012, and the Gua Talimbue remains in 2013 in the Towuti–Routa region of South Sulawesi by Sue O'Connor and David Bulbeck under Indonesian research Visa 278/SIP/FRP/SM/VII/2013, and Visa 315 and described and dated as described above (*53*). The remains are currently stored at the Ancient DNA Facility, University of Huddersfield School of Applied Sciences, with plans to repatriate them to Indonesia in the near future.

An ancient genomic snapshot of Iron Age Wallacea

We performed an ADMIXTURE analysis of FG006, alongside 17 other ancient samples from Wallacea (a pre-Neolithic sample from Sulawesi, ~7200 BP) (*41*), two samples from coastal Sulawesi dating to 250 BP, one from Alor dating to 450 BP, three from Flores dating to 730–2600 BP and ten from the North Moluccas dating to 950–2150 BP (*42*)), as well as ancient genomes from Vanuatu (*27, 28, 29, 100*) and modern dataset 2 (Fig. S8). The lowest cross-validation error value was for K=7.

As we move from K=2 to K=7, we see the deep Eurasian ancestry component (initially orange) partitioning between Taiwanese/Philippine (pink) and Sunda (grey) components at K=7, and the deep Oceanic fraction (dark blue) into various insular fractions (dark blue, pale blue and green) to the east of New Guinea at K=5. Also east of New Guinea, the Eurasian component diverges into a distinct fraction (pale yellow) at K=6 that is spread thinly across Near Oceania but maximised in ancient (~3 ka) Vanuatu and modern Tonga, suggesting an association with the Austronesian expansion in Remote Oceania. This diverges from the East Eurasian grey fraction at K=6, before the latter diverges into distinct pink Taiwan/Philippine *versus* grey Sunda fractions at K=7.

The pink Taiwanese/Philippine fraction is ubiquitous in the later Holocene samples; in pre-Neolithic Sulawesi (7.2–7.3 ka), it is so minor that it may just be noise, and it is also very low in the most ancient Flores sample, in the south. It is the biggest fraction across the Sulawesi and northern (Moluccas) samples, at ~50%. This may therefore have spread primarily in the north, representing Austronesian expansion into northern Wallacea – it reaches higher levels in the south (up to around 25%) only within the last 1000 years. It is present at ~25% in MSEA and China, but does not spread eastwards beyond Southeast Asia, except minimally into New Guinea. This distribution is very similar to that of mtDNA haplogroup M7c3c, proposed as a marker for Austronesian expansion in Southeast Asia (*50*).

The yellow Remote Pacific fraction is also rather ubiquitous in prehistoric Wallacea, except in pre-Neolithic, Toalean Sulawesi, and it is similarly highest in the north, very low in the south,

and intermediate in Sulawesi. Again by analogy with the mitochondrial B4a1a (*61*), this fraction may have arisen from the grey component in the Bismarcks/northern New Guinea, spilling back from there into Eastern Indonesia in the late Holocene.

Conversely, in the south and Sulawesi, but much less so in the north, there is also the grey Sunda fraction. Although Oliveira et al. (*42*) argued for a farming expansion from MSEA, it is present in pre-Neolithic Sulawesi at ~10%. The largest fraction, in Flores (at ~80%), is associated with mtDNA M17a, a very rare lineage seen in modern Malaysian Kensui Semang as well as a few individuals from Thailand, Vietnam and Indonesia, also reflecting an ancient Sunda distribution. Rather strikingly, the dark blue Papuan fraction is also present throughout prehistoric Wallacea – *except* in our Iron Age Sulawesi sample, which carries Papuan Q1 mtDNA (although it is also at very low levels of ~5–10% in the two 250 BP Sulawesi samples). This absence may reflect the fact that Gua Talimbue lies deep inland, whereas the two more recent Sulawesi burials are from a site on the east coast. This fraction is also present (~25%) in pre-Neolithic south Sulawesi (notably, inland), supporting the view based on modern mtDNA variation that interactions between Wallacea and Papua were very ancient (*72*). However, in the Lesser Sundas and the Moluccas, whilst present throughout, it increases over time, suggesting continuous input from the east.

Overall, our exploratory ADMIXTURE analysis of the pre-Neolithic Sulawesi sample indicates Eurasian ancestry (likely South Eurasian) at ~40%, ancient Papuan/Oceanic ancestry at slightly more than 50%, and a small fraction (~10%) of ancient Sunda ancestry (see also (*41*)). These fractions seem likely to be genuine ancestral sources – for example, the South Asian fraction was also inferred by Carlhoff et al. (*41*), and the sample is not especially ancient (~7.25 ka) – supporting the use of ADMIXTURE, which in fact yields a slightly more detailed, if less quantitative, picture of the ancestry of the samples.

Our new 1600 BP Iron Age sample comprises three ancestral sources: around half Taiwanese pink, a third Sunda grey, and ~17% Remote Oceanic pale yellow. We provisionally interpret these as comprising late Holocene ancestry from the Austronesian expansion, ancient ancestry from pre-Holocene Sunda, and more recent backflow from the Remote Pacific, respectively. Despite the Papuan maternal lineage, there is no Papuan autosomal contribution evident, indicating that the Papuan input was some time in the past, and/or rather minimal, and aside from the minor Sunda component, there is almost complete discontinuity with respect to the pre-Neolithic Sulawesi sample (which itself carries the putative pan-Afro-Eurasian mitochondrial haplogroup M1’20’51).

More generally, Wallacean aDNA suggests that the Taiwanese/Austronesian expansion was *via* northern Eastern Indonesia, with a major impact in the Moluccas, which in recent millennia otherwise mainly show just Papuan and Oceanic spread from the east, with little ancient substrate. The mtDNAs seen here are the Papuan Q, Southeast Asian E1a, the “Polynesian motif” lineages B4a1a, B4a1a1, and the very minor, largely Sunda haplogroup M73a.
In the south of Wallacea, there is much more ancient Sunda survival, albeit eroded from ~90% 2600 years ago to only ~20% in the last millennium in the Lesser Sundas (but 50–80% in much of present-day Indonesia, Malaysia and parts of the Philippines). Here, the associated mtDNA lineages are B4a1a1, E1a, M17, and the largely Chinese/MSEA M7b1.

Details of mutation rate re-evaluation

Fu et al. (*44*), using the constant size model and a relaxed clock, obtained a Bayesian estimate of whole mtDNA, which gave a rate of 2.67 x 10^{-8} [2.16-3.16 x 10^{-8}] per site/per year and led them to estimate a coalescence time to the most recent common ancestor for modern human mitogenomes of 157,000 [120,000–197,000] years. Our best estimate of the coalescent time with the Soares rate, using various approaches (*33, 101, 102*), is ~180–190 ka (*e.g.,* using ML and variation within modern Eurasians, 192 (152–234) ka (*33*); using ML and variation within modern Africans, 179 (156–202) ka; using BEAST and incorporating archaic variation, 191 (142–232) ka (*101*). For this age range, we would expect a rate of ~2.2 x 10^{-8} for this time interval, but faster nearer the tips of the tree due to selection. (Their linear regression approach gave 1.92 x 10^{-8} mutations/site/year (1.16–2.68 x 10^{-8}, 95% CI) for the whole mtDNA; this is 0.72x the Bayesian Inference rate and would be too slow.) The Fu et al. (*44*) BI rate is close to the similarly estimated and even faster rate of 2.74 x 10^{-8} [2.44–3.01 x 10^{-8}] mutations/site/year of Posth et al. (*63*), estimated from 66 ancient samples with a range of ages up ~45 ka, but they were mostly Europeans and mostly belonged within a single haplogroup, haplogroup U, as well as a range of modern samples to try to rectify the potential biases.

Note, too, that the rate selected (using the skyline, relaxed rate model) was the fastest of a range of rates estimated under different assumed models, some of which were almost a quarter slower (Posth et al. (*63*), Table 1). Fu et al. (*58*) also re-estimated the rate using the aDNA sample from Ust'-Ishim in Siberia, dating to 45 ka, along with modern samples. With the Ust'-Ishim calibration point, which is closer to the appropriate range for calibrating for the settlement of Sahul, the rate estimated for mtDNA was 2.53 x 10^{-8} substitutions per site per year (95% highest posterior density = 1.76 x 10^{-8}–3.23 x 10^{-8}), which should increase estimates by 1.055 x (only adding ~3000 years in the ~50–60 ka range; even so this would raise the top of the CI for haplogroup P, for example, from 58.4 to 61.7 ka, and for haplogroup S from 57.8 to 60.9 ka – *i.e.,* into the range of the long chronology).

Although it is extremely difficult to find calibration points in the time range 40–80 ka, we are, at least, able to test the Soares rate by comparison with the well dated settlement of the Remote Pacific. It is accepted that the B4a1a1 mtDNA lineage dispersed from ISEA/Near Oceania into the Remote Pacific, initially with the Lapita complex, from ~3 ka (*50, 61*). In Vanuatu, Lapita is dated to 3.0–3.2 ka (*103-105*), and mtDNA haplogroup B4a1a1 is seen amongst the earliest Lapita settlements (*29*), as well as in the earliest settlements in Tonga (*27*).

We therefore re-estimated the ages of B4a1a1 and its immediate descendant, B4a1a1a (*61*) in the Pacific using the haplogroup B "Polynesian motif" sequences from the present project and previous studies (*106*). For an overall founder age estimation in western Remote Oceania, we included data from Vanuatu (n = 103), Fiji (n = 45), Tuvalu (n = 43), Tonga (n = 47) and Samoa (n = 45), with a total n = 283. If we assume that the two founders, B4a1a1 and B4a1a1a, dispersed together into Remote Oceania in a single pulse, the combined founder age should estimate the age of settlement of the westernmost islands, Vanuatu, since this is the point at which new variants begin accumulating along the founder lineages (*107*). Vanuatu's settlement has recently been estimated from Bayesian analysis of radiocarbon evidence at 3000–3200 years ago (*103*), and our founder estimate using the Soares et al. rate was 3200 [2700; 3750] years.

With the Fu et al. rate the estimate was 2800 [2350; 3250]. Thus, while at this timescale the rates do not give very different results, and the CIs on both rates encompass the archaeological estimate, the Soares et al. rate seems to perform better, and we note again that the Soares rate is corrected throughout the tree to allow for selection. The lack of correction in the Fu rate (along with the lack of appropriate calibration points to compensate for it) suggests that the Soares rate is likely to be more accurate at higher time depths.

Soares et al. (*33*) reported that the long-term phylogenetic substitution rate for the entire mtDNA molecule was 1.67×10^{-8} ($\pm 1.48 \times 10^{-9}$) substitutions per nucleotide per year, or one mutation every 3624 years, which we then correct for the effects of purifying selection. Purifying selection has a major impact on the substitution rate in human mtDNA (*33, 108, 109*), and we consider that this is an important confounding factor when evaluating the rate from BEAST. This may be less of an issue when estimating the slower rate of Y-chromosome evolution using this approach (*58*), although it is worth considering that it may also potentially be reducing the age for the male lineage too (*34, 56*).

Detailed phylogeography of mtDNA lineages

Macrohaplogroup M

Overall haplogroup M dates to 67–72 ka (Table S3) and in Sahul is mainly found in the north, *i.e.,* New Guinea and Near Oceania, with a few less common Australian clades (Fig. 1, Data S1). Different subclades of Q1, which diverge 17–22 ka, have distinct distributions. Whilst many are found in mainland New Guinea, some occur largely further east. Some, as has been known for many years, are the result of late Holocene dispersals associated with the spread of Lapita pottery into the Remote Pacific, but older subclades are also found further east. Q1c (10–16 ka) is largely found in the Bismarcks (New Britain) and Solomons, again pointing to Late Glacial dispersals from New Guinea.

This pattern recurs within one of the other two major basal lineages found in northern Sahul, M28. M28 dates to 25–40 ka and is more widely distributed across northern coastal Eastern New Guinea, the Bismarcks (New Britain), the Solomons, Vanuatu and into Fiji and Tonga. Most samples fall in the subclade M28a (20–23 ka), with the remainder forming the sister clade M28b (25–27 ka). Seven of the eight M28a subclades and M28b1 coalesce during the Late Glacial, 11–15 ka, suggesting that Late Glacial dispersal from New Guinea might again be the best explanation.

Haplogroup M27 is possibly the oldest lineage that originated in northern Sahul. It had been previously described as restricted to Bougainville and the other main Solomon Islands (*110*) with four sequences from New Ireland (*111*). The three clades that constitute M27 (M27a, M27b and M27c) diverged from each other between 55–73 ka, while they coalesce respectively at 16–26, 15–25 and 11–15 ka, suggesting a long period of isolation without population expansion between the first peopling and ~25 ka. M27a is mainly found in Bougainville and other northern Solomon Islands, whereas M27b has a more central distribution. M27c, which is ten thousand years younger, is mainly found in Bougainville too. In addition to these samples from Near Oceania, we find that M27 also includes nine samples from Vanuatu. Before this study, only 16 mitogenomes from Vanuatu were publicly available (12 of which are B4a1a1) and therefore,

with 324 new mitogenomes, we provide an unprecedented overview of Vanuatu mtDNA variability. Two of these nine Vanuatu sequences belong to M27c1, alongside a sequence from Madak (in New Ireland, Bismarck Archipelago) and several from the Solomon Islands. The other seven samples constitute the new Vanuatu specific clade, that we have named M27a3b. Here we also modify the phylogeny for M27a3, now only defined by transitions at nps 8857 and 16264, bifurcating into M27a3a (present in Bougainville) and M27a3b (Vanuatu).

Haplogroup M28, with a coalescence age of 25–40 ka, is younger than M27 but displays a similar geographic distribution, covering islands both in Near (New Britain in the Bismarck Archipelago, Solomon Islands) and Remote Oceania (Vanuatu, Fiji, and Tonga). Most of the samples fall into the subclade M28a (20–23 ka), with the remaining 13 forming the sister clade M28b (25–27 ka). Most of the M28a subclades (M28a1 through M28a7) and M28b1 coalesce after the LGM, ~11–15 ka. M28a1 (6–11 ka) and M28a4 (10–20 ka) are only found in Vanuatu. A similar pattern is seen in haplogroup Q1b (7–15 ka).

Although this might suggest a possible early movement to Remote Oceania, almost 10,000 years before that associated with the Lapita culture, other explanations need to be considered in the face of strong evidence that Lapita populations c.3000 BP were entering a previously "pristine" environment, with many species of birds and animals that very quickly became extinct as a result of human arrival, and no evidence of earlier vegetation disturbance as a result of human activity. An alternative explanation might be simply that we have not sampled the immediate source for the Vanuatu lineages, in New Guinea/Bismarcks or the Solomon Islands, or that genetic drift has erased the source lineages over the past 3000 years.

Haplogroup M28a3 was previously defined by four transitions and encompassed seven samples from Santa Cruz; we here add a sequence from the Morobe province (northern Eastern New Guinea Lowlands) that shares the transition at np 10691, thus leaving this as marker for M28a3, with 5063C, 7145T and 16261T defining the new M28a3a. M28a4 was previously only represented by two mitogenomes from Fiji and one from Tonga; here we add 25 new mitogenomes from Vanuatu. M28a4 also includes an archaeological sample dated to 225 (± 20) years before present from Wam Bay, Epi Island (*26*). Two new sequences from Vanuatu share the 9663A with 12 from the Bismarcks (New Britain), thus redefining the subclade M28a5a. Another subclade, where sequences from Near and Remote Oceania coexist is M28a7a, which also includes an ancient sequence from the Mele–Taplins site, on Efate Island (Vanuatu), dated ~2300 BP (*29*). Another newly defined lineage specific to Vanuatu is M28a8. Finally, M28b (25–27 ka) encompasses both samples from Near and Remote Oceania, with modern and ancient sequences from Vanuatu and Futuna in M28b1 and the newly defined (*15*) M28b3 present in Baining (East New Britain in the Bismarck Archipelago) (*78*).

Another ancient lineage which possibly originated in northern Sahul is M29'Q. It encompasses 565 mitogenomes (542 of which are Q), and its two subclades, M29 and Q, diverged ~59–73 ka, thus representing the most ancient major mitochondrial lineage of those restricted to Near and Remote Oceania. The smaller M29 (12–21 ka) is mainly found on the islands in Near and Remote Oceania, with two sequences from mainland PNG. Haplogroup Q includes three subclades; Q1 coalesces around 17–22 ka, with Q2 and Q3 at 35–44 and 20–35 ka, respectively.

Q1 has several subclades originating between 17 and 22 ka, and the subsequent considerable expansion starts ~11 ka.

Most Q subclades, in particular the older ones, are only found in mainland PNG, but there are exceptions. A notable example is Q1b, which is almost exclusively found in Vanuatu, where it most likely originated, with one derived sample from New Britain in the Bismarcks, likely the result of a more recent back-migration. Remote Oceania's other specific Q1 subclades are Q1a1a1a (3–6 ka), Q1a1a4 (1–4 ka) and Q1g3 (2–5 ka), Q1i1a (2–12 ka, which disperses as far as the Cook Islands), and possibly Q1h2b (2–4 ka, which only moves as far as Vanuatu). All of these were most likely carried from New Guinea's northeast coast, where most of the nesting lineages are seen, most likely during the Austronesian dispersals, associated with the Lapita culture.

Q1g is otherwise restricted to the highlands and is likely to have originated there, 16–25 ka. The source for the Q1a1a lineages, which dates to 13–19 ka, is not clear, but interestingly we also see a Q1a1a* lineage in Indonesia, indicating movement in both directions. This is further attested by Q1d, dating to 13–21 ka; Q1d1 is largely New Guinean but Q1d2 has two subclades, one seen in Taiwan, in the indigenous Saisiat (*54*), and one in Madagascar (*55*) – thought on linguistic grounds to have been first settled from Borneo, sometime between the first millennium BC and the mid-first millennium AD (*112*)– and we also see a single Q1d* lineage in the Philippines. Movements to Australia are also evident; whilst Q1d1 is mainly seen in New Guinea, there is also one individual seen in an Aboriginal Australian from Queensland, but with Torres Strait (and therefore likely ultimately New Guinean) ancestry (*12*); and Q1* lineages are also seen in two Aboriginal Australians from northern Queensland, dating to roughly the last 100 years. Furthermore, some subclades are specific to the islands of Near Oceania. Q1c (10–16 ka) has one subclade in Bougainville (Q1c1, 4–10 ka) and one in New Britain (Q1c2, 3–9 ka), plus a small subclade dated to ~2.3 ka in Vanuatu. Q1e (13–23 ka) is found on a few islands in Near (Solomons) and Remote (Vanuatu and Fiji) Oceania, whereas Q1f (3–11 ka) is specific to Santa Cruz.

Our ancient sample from Gua Talimbue, Sulawesi (*113*), radiocarbon dated to 256–393 cal. AD (1710 ± 20 BP: SANU 40418), falls into Q1 as well; it carries the transversion 5447A which characterises the subclade Q1l. Q1l is dated to 9–21 ka, and splits in two clades. Q1l1 encompasses samples from the Eastern New Guinea highlands and one from the northern coast lowlands, whereas the other small subclade is represented by four samples from Vanuatu. The ancient Sulawesi sequence falls outside these two clades and has five private mutations.
Q2, dating to 35–44 ka, is less frequent and virtually absent from mainland PNG. A single sample from Kalumburu, on the northern coast of Western Australia (*11*), constitutes the subclade Q2b, whereas Q2a (15–19 ka) encompasses 55 samples from across Near and Remote Oceania, grouped into subclades dated to ~10–15 ka. These subclades all include samples from both Remote Oceania (Kiribati, Vanuatu, Fiji, Santa Cruz) as well as the Bismarcks (New Britain, New Ireland and Manus) and the Solomons.

On the other hand, Q3, dating to 30–39 ka, is almost exclusively restricted to Eastern New Guinea, with two interesting exceptions. Q3a (23–31 ka) probably originated in the highlands and in the next 30,000 years members of this lineage did not spread beyond the coast. On the

other hand, Q3c (20–34 ka) has been found in one sample from East Timor and another from Kedah, in the Malay Peninsula – potentially another example of bidirectional movements between New Guinea and Southeast Asia along the so-called "voyaging corridor" (*69*).

M7c3c is a subclade of the major East Asian haplogroup M7 and is restricted to Austronesian-speaking populations from Taiwan and ISEA. We have argued that it is likely to be a marker for the Austronesian dispersals out of Taiwan and through ISEA (*50, 114*). We confirm its age here as 5–7 ka, in line with the timing of the archaeological appearance of mixed rice and millet farming from China in Taiwan (*115*). Despite this, the lineage is absent from PNG and is represented in the Pacific only as a single small subclade, M7c3c2, dating to 3–4 ka, seen not in ISEA but only in the Solomons, parts of Micronesia and Tuvalu – all of which are in the same region of the western Pacific and likely in contact over the last 3000 years.

Haplogroup E originated ~25–40 ka and has two subclades, E1 and E2, dating respectively to 16–25 and 11–12 ka. It is found in several regions across ISEA, and as far afield as Madagascar, Near Oceania and Micronesia. It is likely that this haplogroup expanded in ISEA in the postglacial period and only reached Taiwan and Near Oceania within the last 8 ka (*46, 50*). The various subclades clearly have distinct trajectories within the Holocene, as the Oceanic lineages are restricted to certain subclades and entirely absent from others – especially E1a1a1 (dating to 5–14 ka) and E2b (6–8 ka), which are rather oriented towards Taiwan and MSEA.

There are, however, Oceanic individuals within subclades E1a1b (7–8 ka) and E2a (3–16 ka), where several Eastern New Guinea and Solomon Islands individuals are interleaved with a much larger number of individuals from ISEA and Guam. Guam was partly settled from the Philippines, based on linguistic evidence, and recently, genomic data from two skeletons from northern Guam have been retrieved, belonging to E2a (*116*). Haplogroup E1a2 (9–18 ka) encompasses sequences from Indonesia, the Philippines, the Malay Peninsula, Eastern New Guinea and Vanuatu. E1a2a has the greatest fraction of Pacific lineages: whilst clearly displaying an ISEA origin, it includes numerous individuals from Vanuatu and, separately, an entire subclade E1a2a (4–9 ka) restricted to Eastern New Guinea and the Solomon Islands, which likely originated *in situ*, thus testifying to early voyaging between ISEA and Near Oceania.

Attesting further to movements from west to east, a surprising newly identified Papuan lineage is haplogroup M73a2. M73 (38–47 ka) is widespread in MSEA (Thailand, Cambodia and Vietnam) where it probably originated before spreading south-eastwards to Indonesia and Malaysia. It is divided into three subclades, M73c (2–9 ka, only found in Thailand), M73b (31–44 ka, mainly found in Cambodia and Vietnam but also spanning Malaysia and Indonesia), and M73a (19–29 ka), which includes a small MSEA subclade and is also found in Indonesia, the Philippines, East Timor, and PNG. In particular, the transition in np 14502, shared between East Timor and New Guinea, defines a new clade named M73a2 which is then characterised by long branches that separate Timor, the Eastern New Guinea highlands, and the Eastern New Guinea lowlands. M73a2, dating to 15–23 ka, is found deep within New Guinea, with a nesting (or almost nesting) lineage in Timor, suggesting movement eastwards into Sahul at the time of the Late Glacial breakup of the Sunda continent.

In the most recent version of PhyloTree, Build 17 (*48*), haplogroups M42 and M74 share the transition at np 8251, thus forming the node M42'74. This link has been regarded sceptically in the past, because of the relatively high frequency with which the position mutates (22 times in the tree of Soares et al. (*33*), where the range is 0–209) (*12, 117*). With this new set of sequences (including JX462696 from India, descending directly from the M42'74 root), we argue that the most plausible reconstruction now retains the M42'74 node, whilst at the same time losing the M42a'b node. The M42'74 node is dated 55–66 ka, and the two subclades M42 and M74 are dated to 53–68 ka and 33–49 ka, respectively.

While M74 is widespread in MSEA with some younger subclades found in Indonesia and the Philippines, M42 has a striking distribution, with two basal subclades restricted to Aboriginal Australians and the third mainly found in India (*49*). With its single mutation from the root of haplogroup M, this reconstruction thus highlights the southern route out-of-Africa dispersal (*35*). The Indian subclade of M42, M42b, is dated to 47–59 ka, whereas the two Australian subclades date to 33–50 and 46–56 ka, respectively. M42a encompasses 34 samples from Queensland and New South Wales and is divided in three clades. M42c, dating to 46–56 ka, splits into two subclades, dated 39–44 and 48–54 ka, and is widespread in eastern and southern Australia (Queensland, New South Wales, and Victoria). Furthermore, although there is no public access to the sequence data for modern Aboriginal Australian genomes (*31*), they include several M42a sequences from Queensland and New South Wales, and M42c sequences from Western and South Australia. The phylogeography of haplogroup M42 thus suggests separate settling times and movements for the different clades and, given that M42a and M42c are restricted to Australia and therefore likely arose *in situ* (at a time between the ages of M42a and M42c and the ancestral M42), also suggests Australian arrivals by ~60 ka.

Macrohaplogroup N

Haplogroup N overall in Sahul is dated 63–73 ka (Table S3) whereas its major subclade, haplogroup R, dates to 60–66 ka. In general, Australian N lineages seem older than New Guinean ones (Fig. 2, Data S1), most likely due to major re-expansions much later in New Guinea (*23*), as in South Asia (*35*) but not in Australia.

Haplogroup N13, despite encompassing only four sequences from Aboriginal Australians, has been considered one of the Australian-specific haplogroups (*12*) (although see Bergstrom et al. (*23*)). We have found and sequenced ten more mitogenomes belonging to this clade, four from Queensland and six from mainland PNG, with the nesting suggesting that it spread from Australia to New Guinea. N13 is dated to 27–46 ka, and now splits into two subclades, N13b, with only two samples from Queensland, and N13a (29–42 ka) found in both present-day Australia and mainland PNG (the latter including N13a2, dating to 9–20 ka, and one nested within N13a3, which dates to 6–18 ka) and in an archaeological specimen from Mapoon radiocarbon-dated ~100 years old (*43*). The two small subclades thus penetrated New Guinea within the last 20 ka, pointing to multidirectional mobility and connection, albeit at low levels.

Haplogroup O (28–38 ka) encompasses a total of 52 samples, separated into two subclades dated to 20–38 ka (O1) and 21–30 ka (O2). In both, samples in basal clades are mainly found in the north (Queensland, Northern Territory) whereas the more derived ones are in South Australia, New South Wales, and the south of Western Australia.

Haplogroup S is the largest Aboriginal Australian haplogroup, and with a coalescence age of 51–57 ka is also one of the oldest. It is divided into two main clades, S1 (33–52 ka) and S2 (42–58 ka), plus the smaller S6 (4–14 ka), and the minor S3, S4 and S5 which are represented by only one or two sequences each. This haplogroup is mostly found in eastern Australia, with only three samples from Western Australia (although there may be sampling bias at play here). S1 is largely found in Queensland, with two small, derived subclades in Southern Australia. S2 is equally present in Queensland, New South Wales, and South Australia, and is also found in the Northern Territory and, with a small S2a2 clade, on the southern coast of Eastern New Guinea. S2a2 is dated 16–28 ka, and the basal Papuan clade suggests that Late Glacial movements from Queensland were northwards as well as southwards. S3 and S4 only comprise, respectively, two and one sequences from the Northern Territory, whereas S5 is a single mitogenome from Western Australia. S6 is found in South Australia, Victoria, and New South Wales. All these small lineages are separated from the root of haplogroup S by long branches, suggesting a very long period of drift after the ancestors of haplogroup S first arrived in the continent.

Haplogroup P, dating to 60–63 ka, is the largest haplogroup shared across Sahul (and with ISEA). It is widespread throughout Sahul (with 325 mitogenomes from Eastern New Guinea and 130 from Australia) but is also found in Remote Oceania (69 samples from Vanuatu plus some from Santa Cruz (north of Vanuatu), Fiji and Tonga), the Solomon Islands and ISEA (Philippines, Indonesia, East Timor, and the Malay Peninsula). P1 encompasses 315 samples and has a coalescence age of 31–35 ka; it clusters (albeit *via* a single, fast-evolving control-region variant at np 16176, so both subclades may in fact be basal to P) with P10, which is seen only in the Philippines. Despite its very early divergence, P10 has very little diversity, amounting to only 2–9 ka. P9, too, is uniquely found in the Philippines but dates between 45–59 ka.

P1 dates to 25–31 ka and has several subclades, some restricted to New Guinea (P1a, P1c, P1d3 and P1d4), others to Near and Remote Oceania (P1f, P1d2a) and the remaining widespread across northern Sahul, with a few samples in ISEA. P1d2a (9–12 ka) is found mainly in the islands of Near Oceania and Vanuatu and P1f (15–26 ka) is also common in Vanuatu; and various minor lineages have spread to Timor, Indonesia, and northern Australia within the last few thousand years. (The suggestion of Pedro et al. (*15*) of much earlier dispersals of P1 is not supported by the combination of singleton lineages and very small subclades seen in these regions.)

Haplogroup P2 dates to 44–50 ka and is mostly found in the New Guinean highlands but has two subclades in Vanuatu and in Queensland. P2a2a in Vanuatu dates to only 1–4 ka, but P2b has basal branches in both New Guinea and Australia and dates to 11–22 ka, so may have spread south with the Late Glacial.

P3 similarly dates to around 42–53 ka, but it likely originated in the north of Australia. P3a is wholly Australian, but P3b (36–47 ka) is mixed, indicating very ancient contacts between Australia and New Guinea. There is a pre-P3b1 subclade (defined by a transition at np 16399; 34–44 ka) with three Aboriginal Australians but one nested New Guinean mitogenome, and P3b2 (33–44 ka) that is mainly New Guinean but includes an Australian basal to P3b2a (29–40 ka).

P4 probably diverged in the New Guinean highlands from ~20–29 ka and has experienced recent gene flow to Indonesia and Vanuatu.

All the remaining subclades, P5 (27–39 ka), P11 (56–64 ka) and P12 (44–57 ka) and the more minor subclades, P6 (40–58 ka), P7 and P8, are only present in Australia, with the oldest (P11) supporting once again the arrival of the first settlers in Sahul by ~60 ka, and several others suggesting a likely divergence within Australia more than 50 ka.

Bayesian skyline plots

Bayesian skyline plots for Eastern New Guinea and Pacific mitogenomes indicated an initial major rapid expansion at 60–64 ka (Fig. S1). There is also a signal of expansion in the Bismarcks and Solomons at ~60 ka, and a similar signal even in Vanuatu, but in the latter many lineages are shared with Eastern New Guinea, so this undoubtedly reflects an echo of the expansions in Eastern New Guinea/Near Oceania rather than a unique expansion within Vanuatu. There is less overlap of lineages between mainland PNG, the Bismarcks and the Solomons, suggesting that part of the early expansion signature in the Bismarcks and the Solomons may be meaningful, but there is sufficient overlap to make such an interpretation inconclusive.

Y-chromosomal evidence estimates of out-of-Africa dispersal and Sahul settlement time

The published works we employed use a common Y-chromosome mutation rate, a Bayesian calibration using the Ust'-Ishim aDNA sample, dating to 45 ka from Siberia, of 0.76×10^{-9}/ site/year (*58*). This differs from the mtDNA estimation by scaling the number of missing mutations compared to present-day samples to the age of the sample, and yielding a rate which is slightly slower than the rate adopted by the YFull online resource (https://www.yfull.com/tree/) (*118*).

We note that the out-of-Africa dispersal model we propose for the Y chromosome, in which DE-M145 diverged within Africa and CF-P143 diverged outside Africa, in fact most closely mirrors the one assumed for mtDNA, since no pre-M or pre-N lineages have been discovered within Africa (and a pre-N lineage has been found in aDNA outside Africa, from Oase in Romania (*119*)). Similarly, to date, no pre-D(xD0) or pre-CF lineages have been seen in Africa, although, given the lack of aDNA of similar age to Oase in Africa, and the high drift on the Y chromosome, we cannot rule it out.

Haber et al. (*56*) argue for a more constrained estimate of 50.3–59.4 ka for the exit from Africa, but this was on grounds of the timing of Neanderthal DNA introgression – dated by Fu et al. (*58, 120*) to 49.9–59.4 ka, or 51.7–57.7 ka – not their analysis of Y-chromosome lineages. Teixeira and Cooper (*17*) and O'Connell et al. (*18*) have also argued against the long chronology on these grounds. Moreover, Denisovan introgression is thought to have taken place after the Neanderthal introgression, and presumably before the entry to Sahul, potentially constraining the timing still further.

But these estimates necessarily postdate the dispersal from Africa, depend on the value assumed for human generation time, and disregard uncertainty in the radiocarbon dates and autosomal

mutation rates. They may also be lower-bound estimates, if (as is thought to be the case) there were multiple episodes of Neanderthal introgression after modern humans emerged from Africa. It is not even entirely clear if they are analysing the same introgression event that resulted in the Neanderthal genome fragments present in Sahulian genomes. Jacobs et al. (*78*) estimated two episodes of distinct Denisovan introgression into the ancestors of Papuans, at 32–61 ka and 14–50 ka respectively (the latter estimate implying Denisovan presence in Sahul itself; although see the alternative interpretation of Browning et al. (*121*)), but also stressed that these were minimal estimates. Given these uncertainties, alongside the likelihood of a rapid dispersal from Africa to Sunda and Sahul, we would argue that the timing of archaic introgression cannot decisively rule out the long chronology – although we also note that estimates of Neanderthal admixture are somewhat in tension with the long chronology (see below).

Note that the ages in the online resource YFull are somewhat younger than those of Bergström et al. (*16*). For the upper bounds, C1b-F1370 dates to only ~47 [44–51] ka in YFull and K2b-M1221 dates to only ~44 [43–45] ka. Although higher than YFull, the estimates of Bergström et al. (*16*) may themselves be underestimates. For example, Helgason et al. (*122*) have shown that the Y-chromosome rate is higher when estimated from pedigree data, pointing to a possible influence of purifying selection (with slightly deleterious mutations being selected out over time), as observed for mtDNA (*33, 108*).

We constructed a Y-chromosome SNP tree from the mainland PNG data of Bergström et al. (*23*) (Fig. S2), to complement the geographic profile and whole Y-chromosome tree presented there, as well as the whole Y-chromosome trees presented in Bergström et al. (*16*) and Haber et al. (*56*). Our Y-SNP tree displays the pattern shown previously for data (typically ~10 Mb) from limited numbers of Y-chromosome full sequences: there are three major Sahul clusters, one within haplogroup C and two within haplogroup K.

The C lineages belong to the single mainly starlike C1b2a-M38 subclade with low diversity (ρ = 2.77; we cannot convert this directly to an age, due to the ascertainment process of the SNP assay). This relative lack of diversity is also evident in the full Y tree (C2 in Bergström et al. (*16*), dating to ~21 ka: Fig. S13 in Bergström et al. (*16, 23*)) and suggests heavy drift since the split from southern Sahul, perhaps reflecting the impact of Late Glacial and Neolithic expansion (*23*). The restricted distribution of the C1b2a-M38 lineages within Eastern New Guinea (mainly within the Madang province) contrasts with both the age of C1b2a-M38 and the presence of C1b2b-M347 in Aboriginal Australians (C4 in Bergström et al. (*16*)). These considerations support the idea of more recent drift, although we should be wary of the lack of male samples from the southern regions in the dataset (see also Bergström et al. (*23*), their Fig. S5).

The other major cluster includes several paraphyletic K2b1-P399* lineages at its root. The presence of these paraphyletic lineages in New Guinea, but not in Australia, might be seen as the trace of a northern-route dispersal, but the high drift on the Y chromosome makes such an inference hard to test. There are no SNPs defining an additional K2b1a subclade and no indication from full Y-chromosome trees that such a clade exists: *e.g.,* (*16*); although we note that the reconstruction of Haber et al. (*56*) for this part of the tree does not quite seem to match the detailed phylogenies proposed by ISOGG 2019 or YFull (*118*), where both haplogroups M and S are derived with respect to haplogroup K2b1-P399.

Of haplogroups S and M1, the former is fairly starlike and markedly less diverse than C1b2a-M38 (with ρ = 1.05) whereas the latter is less starlike and much more diverse (ρ = 3.52). Nevertheless, the full Y-chromosome tree indicates that all three of these major clusters arrived with the first colonisation and their differing diversities (and therefore age estimates), as with the mtDNA variation, are due to chance and differing demographic trajectories following arrival in Sahul.

Haplogroup S is also shared amongst northern and southern Sahul populations, with the full Y tree suggesting an ancient divergence between northern and southern groups. Early branching lineages are seen in Indonesia and the Philippines (S3 and S4), including within the aboriginal Aeta people of Luzon (S2), as with mtDNA haplogroup P, with S1a lineages interleaved between north and south, and with the northern lineage shared across Wallacea, New Guinea and Near Oceania, again calling to mind the distribution of mtDNA haplogroup P. On these grounds, although Y-chromosome lineages are much less amenable to deep phylogeographic reconstruction than mtDNA (due to the higher levels of drift resulting from the high variance in offspring for men compared to women), we might regard both haplogroups C and S as having arrived via the northern route into Sahul.

Unlike C1b2 and S, haplogroup M-P256 is largely restricted to the north, with signs of a recent Holocene southern dispersal into Torres Strait islanders (*16*). M2-M353 and M3-P117 are seen in New Guinea and the Bismarcks, respectively, and M1-Z30983 is mainly seen in New Guinea, with the rare M1b-FT254675 in Wallacea. Aboriginal Australian individuals belong to the highly derived M1a3b1b-Z42297 subclade. The widespread but northern distribution of haplogroup M-P256 resembles that of the mtDNA M29'Q lineages, again pointing towards a northern-route ancestry.

Recombinational dating and the out-of-Africa dispersal

Several authors have estimated that Neanderthal admixture occurred ~10% earlier than Denisovan admixture (*31, 123*), and this relative difference should be more robust to factors like generation times than absolute estimates. A settlement of Sahul ~60 ka, with at least one episode of Denisovan admixture occurring just before this time, would therefore imply Neanderthal admixture ~60 x 1.1 = 66 ka. This would be considerably earlier than some published estimates of Neanderthal admixture. For example, Moorjani et al. (*124*) estimated that the Neanderthal admixture took place ~7 ka before the Initial Upper Palaeolithic Siberian Ust'-Ishim individual lived – thus, ~49–55 ka, as Ust'-Ishim dated with radiocarbon to 46,880–43,210 cal. BP (95.4% probability). Whilst employing estimates of the human autosomal recombination rate, as well as of the generation time, these values also make assumptions about the number of Neanderthal introgressions episodes, suggesting that the level of uncertainty may be underestimated, but the discrepancy clearly deserves further investigation. Using a mutation rate rather than recombination rate estimate, albeit neglecting uncertainties in the radiocarbon date of Ust'-Ishim and the generation time, Fu et al. (*58*) had previously estimated a date of ~52–58 ka, which is also more recent than our proposal. On the other hand, we also note that an earlier date of ~66 ka would be highly consistent with Neanderthal admixture in the Gulf region shortly after an emergence from Africa ~67–69 ka, as proposed here.

The discrepancy has, however, been exacerbated by two recent estimates of the Neanderthal introgression time. Iasi et al. (*39*) analysed 59 ancient genomes sampled between ~45 and 2 ka, and 275 modern genomes, but focused on 22 individuals >20 ka for the dating – 16 between 40–24 ka and six >40 ka. Their best estimate for the Neanderthal introgression time was an extended pulse, with the mean time of gene flow of ~47.1 [46.9–47.4] ka, with a duration of around ~6.8 [2.0–10.0] ka. They estimated that all modern non-Africans post-dating 40 ka had Neanderthal segments from only this pulse, which occurred between 50.5–43.7 ka.

At the same time, Sümer et al. (*40*) estimated an even more precise age of introgression of ~47 [45.4–49.4] ka from seven pre-40 ka genomes. The remains they analysed were from two sites in central Europe with related genome composition, which they inferred to represent the deepest split amongst non-African populations. The German site (Ranis) radiocarbon dated to 42.2–47.0 ka, whereas the Czech site (Zlatý kůň) could only be dated by genetic approaches, to ~47–48 ka; one individual from each site was sequenced to high depth. They inferred that these early individuals, too, had experienced only a single, extended pulse of Neanderthal introgression, ancestral to all the Neanderthal segments across the non-African population down to the present day. Other pre-40 ka individuals, such as Oase1, may have experienced subsequent pulses, but, they argue, did not contribute their additional Neanderthal admixture to post-40 ka generations.

These two estimates date the time at which the nascent non-African population formed a single inter-breeding group, well before the splits between west Eurasia and east Eurasia/Sahul. This would imply an upper limit to the major Eurasian split of ~50 ka; the analyses suggest more likely not much more than ~45 ka. Moreover, the Denisovan admixture, estimated to take place at ~90% the time of the Neanderthal introgression, cannot have occurred much before 40 ka. As Sümer et al. (*40*) acknowledge, this means that all modern human skeletal and material culture remains outside Africa preceding 50 ka must belong to populations that left no descendants.

For Sahul, particularly if we assume that the main episode of Denisovan admixture took place before modern humans crossed into the continent, this implies that all remains prior to ~42 ka would represent groups who were not ancestral to modern Sahul populations. In other words, these results imply an enormous revision of the way in which the archaeological and paleoanthropological record are interpreted. Yet recombination dating is in its infancy and relatively untested. The extent to which variation in recombination rates can be allowed for is not known, and the critical specimens are at the limits of radiocarbon dating. Recombination is a biologically complex process, and its modelling to estimate dates is far from straightforward. Estimates critically depend on the accuracy of recombination maps (*125*), reliable haplotype phasing and sufficient haplotype diversity to allow confident detection of recombination events, all factors leading to varying levels of uncertainty in the estimates. Given these limitations, and our estimates suggesting that the mutational clocks – which have been successfully tested against known colonisation events over many years – can be reconciled with the archaeological picture, we do not think the entire framework of non-African modern human dispersals should be abandoned just yet.

Details of genome-wide patterns

As noted in the main text, we performed the two sets of analyses: analysis 1 (including more Aboriginal Australians: Fig. S3) and analysis 2 (including more Indonesians and Pacific islanders: Fig. S4). Here we describe the order of splits in the two analyses in more detail. As anticipated, the deepest split **(*K*=2)** in both analyses is between Sahul and Eurasians – although we see a small fraction of the Sahul component in India at *K*=2 and *K*=3. This likely reflects the Andamanese-related ancestry in South Asians, which is closer to ancestry in Sahul than East Asia (*32*). In addition, the Aboriginal Australians from New South Wales include a substantial European fraction indicating recent admixture. This is consistent with previous genome-wide analyses, and with analyses of uniparental markers suggesting sex bias within the last few hundred years: the great majority of female lineages are indigenous, but many male lineages indicate European descent (*13, 31, 126*).

Most strikingly, the second deepest split **(*K*=3)** in both analyses is *within* Sahul/Near Oceania – separating New Guinea from the islands of Near Oceania (the Bismarcks and Solomons). The next split **(*K*=4)** in analysis 1 separates Aboriginal Australians, albeit with a fraction shared in part with South Asians. Those from Arnhem Land and those from New South Wales share a major indigenous component, although the latter also share a European fraction that varies from around zero to 100% (Fig. S5). Although artefactually deep splits can appear due to genetic drift, this would tend to imply a small, isolated population, whereas the split here encompasses numerous populations and, in the case of Near Oceania, many islands (although note that ADMIXTURE works with total drift rather than shared drift). Therefore, given the mitogenome results, we suggest that the early emergence of this split implies that the divergence within northern Sahul may have begun soon after the first settlement, perhaps even before the split between New Guinea and Australia (although there is the additional caveat that there are more Near Oceanian than Australian data, which might lead to earlier splits). However, the link to South Asia is intriguing and may be due to ADMIXTURE modelling the Aboriginal Australians as Pleistocene South Asia + New Guinea + Oceania.

At ***K*=5**, there is a split within New Guinea itself which resolves southern and western from the eastern highland regions. The next major split, at ***K*=6,** is within Near Oceania, with New Britain on one side and New Ireland plus the Solomons on the other. This indicates a very ancient split across island Near Oceania, again pointing to very early settlement and divergence, as clearly seen in the mtDNAs.

***K*=7** isolates a minor fraction from Taiwan and ISEA, which is also seen at low levels in coastal New Guinea and throughout most of Near Oceania (except where there are signs of recent heavy drift). It reaches higher levels in the Solomons (except Bougainville), and peaks at ~80% in Tonga – and, as we can see from analysis 2 (where it emerges earlier, at *K*=4, in the context of greater sampling of ISEA variation), also Samoa and Tahiti. We note that this is the Southeast Asian cluster that appears at high frequency in the New Guinea lowlands, especially the Central province (*23*). Note that we see no sign of Macassan, *i.e.,* recent Sulawesi, influence in Australia. This component looks very much like the Holocene "out-of-Taiwan" (or Philippines) dispersal of Austronesian speakers into the western Pacific, associated with Lapita pottery, mtDNA haplogroups M7c3c, Y2, F1a4a, B4c1c (but not the "Polynesian motif", B4a1a1a) and Y-

chromosome lineages within haplogroups O1a (see Fig. S2), O2a1 and O3. We previously estimated they contributed ~20% overall to the late Holocene ISEA gene pool (*50*), very similar to what we see for this component here. Higher values of *K* for both analyses suggest a possible source in China for this component, fitting with the ancestry of mtDNA haplogroup M7c3c and the source of the Formosan Neolithic in Taiwan (*115*). Notably, though, this component is more common in the Solomons than in the Bismarcks.

***K*=8** finally separates the Australians from the similar ancestry trace in India, whereas ***K*=9** and ***K*=10** simply separate several islands experiencing recent drift within the Bismarcks.
We note in passing that our interpretation of the *K*=2 split in the ADMIXTURE analyses implies substantial Eurasian admixture in Near Oceania, which is well established. But, in addition, there seems to be a major Eurasian coastal incursion into the central Eastern New Guinea highlands, also evident in the *K*=2 analysis of Eastern New Guinea in Bergström et al. (*23*), and evidently corresponding to the high levels there of Y-chromosome haplogroup C-M130, but evidently *not* to mtDNA haplogroup B4a1a1 (see (*23*), Fig. S3–S5). This pattern of limited, recent introgression of haplogroup C into the central highlands is also seen in several groups in West New Guinea, where the lineages belong mainly to the derived C1b2a1-M208, which is also the main male lineage we see in Polynesia (*60*). This pattern implies a distinct trajectory in some respects of the male C1b2a1-M208 lineage from the female "Polynesian motif", even though both are heavily involved in the late Holocene settlement of the Remote Pacific (*61*).

Detailed evaluation of current dating evidence for *Homo sapiens'* occupation of Sunda, Wallacea and Sahul

This section reviews 25 studies reporting pre-40 ka evidence for the occupation of Sunda, Wallacea and Sahul. Each has pushed back the age for occupation in their region during the Late Pleistocene (MIS 3 and 4), with evidence typically provided by radiocarbon or luminescence dating, though U-series and electron spin resonance (ESR) have also been effectively used in some studies (Table S6).

Sunda

The oldest confirmed sites are Lida Ajer in Sumatra (*6*) and, more recently, Tam Pà Ling in Laos, at 67–73 ka, or even 68–86 ka (*8*). Direct dating of human teeth with U-series and ESR at Lida Ajer has established the age of the occupation of this cave, with U-series of flowstones bracketing the teeth-bearing breccia confirming the dating. Thermoluminescence dating of breccia from elsewhere in the cave shows close alignment, though the stratigraphic relationship is less certain. While the association that has been used to model the age-range of this deposit can be questioned, changing the stratigraphic relationship of the dates would make little difference to the resultant age estimates. This study therefore places occupation of Sumatra between 73–63 ka. At Tam Pà Ling in Laos, combined U-series-ESR dating suggested an age of 67–73 ka for a partial frontal bone, identified as belonging to anatomically modern *Homo sapiens*, and 68–86 ka for an unidentified tibial fragment (*8*). Whilst the authors assume that this extends settlement by *Homo sapiens* beyond 70 ka, implying an earlier dispersal from Africa than that carrying the ancestors of modern populations, an alternative explanation consistent with

our dating scheme would be that the frontal bone might be part of the main settlement with the tibia belonging to earlier archaic settlers.

Dates from the Lang Rongrien rockshelter, Malay Peninsula (*127*) and Niah Cave, Borneo (*128*) should only be interpreted as minimum dates for occupation, rather than absolute ages. Meanwhile, the dating of rock art has become increasingly important to the debate (*129*). The U-series dated flowstones in the Lubang Jeriji Saléh cave, Borneo (*130*) offer an age-range for occupation between 52–40 ka. However, the maximum age hinges on a single date – LJS2.5 – underlying the pigment layer, with the flowstone over this pigment dated 20.3–26.1 ka. The study assumes that date LJS2.5 is representative of all flowstones present under pigment layers, though in the other sampled sequences those flowstones were dated between 100–500 ka. The dating of this cave sequence would therefore be better considered as providing an age >40 ka.

Wallacea

Studies on flowstones within the Maros-Pankgkep caves of South Sulawesi have suffered the same issues as that of Lubang Jeriji Saléh, where only minimum ages for the cave art can be derived from the flowstones overlying the pigment layers, with Leang Timpuseng (*130*) providing the earliest limiting age of >40 ka and Leang Tedongnge an earliest limiting age of >45.5 ka (*131*).

At Leang Balangajia 1, sampling in two locations of the coralloid underlying the pigment layer produced ages of 82.1 and 72.6 ka, while the coralloid immediately overlying the pigment layer produced ages of 29–32 ka. This U-series dating suggests that the suid figure in Leang Balangajia 1 was painted sometime between 73.4–32 ka (*131*).

More recently, however, a novel laser-ablation U-series imaging approach has re-dated a hunting scene from Leang Bulu' Sipong 4 to a minimum age of 50.2 +/-2.2 ka, and dated narrative cave art at Leang Karampuang, including anthropomorphic figures and animals, to a minimum age of 53.5 +/- 2.3 ka, making it currently the oldest known evidence for representational art and visual storytelling globally (*132*).

The bone assemblage from Tabon, Palawan (*133*), gives a wide range of dates on the three U-series-dated bones. Although the oldest date quoted is for a tibia discovered in 2000, dated 47+11/–10 ka, the other two bones (from a 1962 excavation) are 31 and 16.5 ka. Earlier radiocarbon dating of this site is also very mixed, suggesting the assemblage is not stratified and so the dating should only be accepted tentatively, with many caveats.

On Tanimbar, occupying the easternmost point of southern Wallacea, new studies have been undertaken at the Elivavan, also known as Watu Eli, rockshelter (*134*). Radiocarbon dating was undertaken on marine shell recovered from the lowermost main stratigraphic layers within this site. The oldest date from the lower stratigraphic layer (Phase 1 occupation) was from marine shell (calibrated against Marine20), providing a modelled calibrated age of 42.2–40.5 ka (*95.4% probability*; Wk-56087), with marine shell from overlying spits sampling this layer providing calibrated ages between 39.3–33.6 ka.

From Timor, three rockshelter sites provide dating evidence for early occupation. At Laili (*135*), dating is based upon radiocarbon only. The dates from the base of the sequence (units 8-11) show intrusive material, with the oldest date from the base of the sequence (D-AMS-007344; 40,417±332 bp) a notable outlier. With no other supportive dating controls, or additional radiocarbon dates this old, the site is not securely dated. At Asitau Kuru (*136*), the OSL and radiocarbon dating show some agreement, though the dated occupation layer is presumed to result from a protracted period of sedimentation. No OSL dating is associated with the deepest contexts, while the basal radiocarbon-dated shells have returned a wide range of ages. This radiocarbon dating should therefore only be used to suggest minimum occupation age >43 ka. Finally, at Lene Hara (*137*), the earliest dating (cited as demonstrating a 42-ka occupation) is based upon two dates within a breccia. There is an assumption that the breccia is single phased, with the shell and artefacts contemporary.

Unfortunately, no other dating technique (notably U-series) has been attempted on the flowstone coating of the Breccia which could have constrained the age of this deposit. No special pre-treatment of the shells was undertaken, with all other dating in the rockshelter (non-breccia) c.30 ka, and associated with much richer cultural assemblages. The dating for this site should therefore be accepted with some caveats. Overall, the dating of sites from Wallacea should in most cases be regarded as minimum ages, confirming modern humans arrived before c.43 ka.

Sahul

Dating from Sahul is much more extensive. For New Guinea, two archaeological sequences provide dating evidence. On the Huon Peninsula (*138, 139*), artefacts within a series of tephra layers have been dated to at least 40 ka using thermoluminescence. The reason why artefacts are located within tephra layers is not addressed, and questions have been raised over the calibration of the dates, with Roberts (*140*) suggesting that recalculation of the ages could provide an age range of 47–61 ka. With these uncertainties, the original dates published by Groube (*139*) should be considered a minimal age. The other study is from Vilakuav, Ivane Valley (*141*), where a number of sites have been identified and are correlated with their stratigraphic sequence. The study clearly shows that multiple cultural layers are present during the Late Pleistocene, with the radiocarbon dates confirming occupation in the 40–48 ka period. However, no detailed stratigraphy of each site is provided for further interrogation, which could establish the association between the dates and archaeology.

For Australia, the oldest sites are all situated in the Northern Territory. Studies at Nauwalabila I (*142, 143*) and Madjedbebe (*5*) both utilise radiocarbon and OSL dating and provide the oldest ages for occupation of Australia. At Nauwalabila I, there is good agreement between the dating techniques, with two dates constraining the base of the lowest occupation horizon, suggesting earliest occupation is between 53–60 ka. A similar pattern is also seen at Madjedbebe, this time with a much more extensive dating strategy. The lowest occupation horizon is bracketed by multiple OSL dates, which are replicated across four excavated sections. While the authors state that this lowest occupation layer dates between 65–52.7 ka, some caution is necessary. Some artefacts are observed deeper in the sequence, one associated with an OSL date of between 73–82 ka, with vertical spread of the lowest occupation layer also evident. It could be argued that the lower boundary of the archaeological layer is poorly defined, with several >60ka dates below the true occupation layer incorporated into its age estimate. While a minimum of 53 ka seems

beyond question, the true age range of this occupation may be within the 53–60 ka range, which would mirror the findings from Nauwalabila I.

The third rockshelter from the Northern Territory is Nwarla Gabarnmang (*144*). This site has a similar archaeological record to the other two sites, consisting of a low-density basal artefact horizon, with a much denser concentration of finds higher in the sequence. Radiocarbon dating indicated that occupation occurred 45.6–52.2 ka, much younger than the other two sites. However, the radiocarbon dates do not extend to the base of the artefact-bearing SU4 (artefacts are present up to 20cm below the lowest radiocarbon dates), meaning that the time of first occupation must precede their quoted age, and therefore older than 47–49 ka. This could suggest that the lowest occupation horizon is contemporary with those of Madjedbebe or Nauwalabila I in the 53–60 ka period. The final dated site is an open-air deposit with very sparse finds from Minjiwarra (*145*). This study claims the earliest occupation as 47.2–51.4 ka, but it is based upon a single complete flake with a corresponding OSL sample from the same elevation. While this age is comparable to other dated sequences in the area, the archaeological significance of this site and its date need to be treated cautiously.

In Western Australia, four sites show occupation >44 ka. For the Carpenter's Gap rockshelter (*146*), extensive (>100) radiocarbon dating has been undertaken using a range of different pre-treatments. The site shows repeated late Pleistocene occupations, though the earliest phase SU8, estimated as 44–51 ka, provides ages at the limit of radiocarbon dating, so these may be best regarded as minimum ages for occupation. At Riwi (*147*), extensive radiocarbon and OSL dating was undertaken through the stratified sequence and provide good agreement, with bracketing dates for the lowest occupation layer, giving an age of 44.6–46.4 ka. The Parnkupirti open air site (*148*) contains a stratified sequence with clearly defined archaeological horizons, dated using OSL. There are some limitations on dates from Site 3 (basal) due to saturation, meaning these should be interpreted as 'minimum ages'. However, there is consistency between the dating of the two sequences bracketing the archaeology, with an age estimate of 45–50 ka. On the northwest coast, Boodie Cave (*149*) is dated using radiocarbon (on marine molluscs) and OSL. The base of SU8 appears to be truncated, suggesting a c.20 ka hiatus in sequence, which the authors recognise could mean that earlier occupation cannot be ruled out. The dating of SU9 is 70 ka and interpreted as indicating a period when the entrance to the cave was more restricted, due to the high concentration of rodent remains, which could have limited human access to the cave.

The rockshelter of Karnatukul (Serpent's Glen (*150*)) contains extensive Holocene assemblages, though the deepest SU6 has provided a series of Pleistocene dates, albeit covering a very wide range. Only three of the six basal dates are >40 ka. Some caution needs to be applied to this study as clearly there are several hiatuses in the sequence and some reworking. The basal Pleistocene archaeology is low in abundance but sufficiently unique to not be a contaminant from the large Holocene assemblage overlying it, with the evidence best interpreted as giving a minimum age of 47.8 ka.

The final site from western Australia is the cave of Devil's Lair (*151*). The site has been extensively dated, most notably using radiocarbon dating with a range of different pre-treatments. While the original radiocarbon dates placed the site c.30 ka, further dating using

ABOX pre-treatment, coupled with OSL dating, has pushed the age back to c.50 ka. ESR on macropod teeth provided over-estimated ages, which the authors accept could have been calibrated if U-series were undertaken on the teeth too (like in the Lida Ajer study). U-series on flowstones is used to help constrain the basal age of this sequence.

In New South Wales, the Lake Mungo burials represent the oldest human remains in Australia. Bowler et al. (*152*) focused on the stratigraphic context of these burials, notably Mungo III, whose skeleton had previously been directly dated using ESR at 63±6 ka, with Th/U suggesting an age between 50.7–82 ka. OSL dates that could bracket the burial established that this skeleton was likely 40±2 ka. The oldest archaeology at Lake Mungo consists of 11 stratified flakes bracketed by OSL dates of 45.7±2.3 and 50.1±2.4 ka.

In South Australia, the rockshelter sequence at Warratyi (*153*) has been dated by OSL and radiocarbon. The early occupation of this site, estimated at 49 ka, is based on the presence of pigments in the sequence, with artefacts (bone and hafted tools) only appearing after 40 ka. The authors state that activity is ephemeral around this site, so its use for inferring first arrival should be treated cautiously.

Summary

The existing body of dating for pre-40 ka occupation of the Sunda, Wallacea and Sahul regions is compelling and shows strong agreement across multiple studies. The narrative pushed by some, that deep chronology is only provided by OSL, while the more accepted <50 ka occupation is radiocarbon based, is now a falsehood, as many studies have integrated the two dating techniques and shown they can provide good agreement on the age of deposits. In many studies, only minimum ages for occupation can be established, either because the dating of underlying 'sterile' stratigraphic units is missing, or the use of radiocarbon dating has hit the detectable limit of the technique with radiocarbon calibration. IntCal20 (which contains ShCal20 and Marine20 applicable for southern hemisphere studies) is now capable of pushing radiocarbon date calibration back to 55 ka, providing another opportunity to reassess the chronology of some of these sites, potentially leading to more in the >50 ka age range.

Authors tend to quote the maximum possible age for a site rather than a more conservative, and statistically robust, estimate – this is certainly the case for Madjedbebe, where a cautious estimate of 53–60ka, in line with dating from other sites in the Northern Territory, is most likely. If these sites are representative of the first arrival into Australia, then the timing would be around the MIS4–3 transition. Outside Sahul, only Lida Ajer appears to provide a robust age estimate for actual occupation (rather than just a minimum date) of 63–73 ka, within the early to middle part of MIS4.

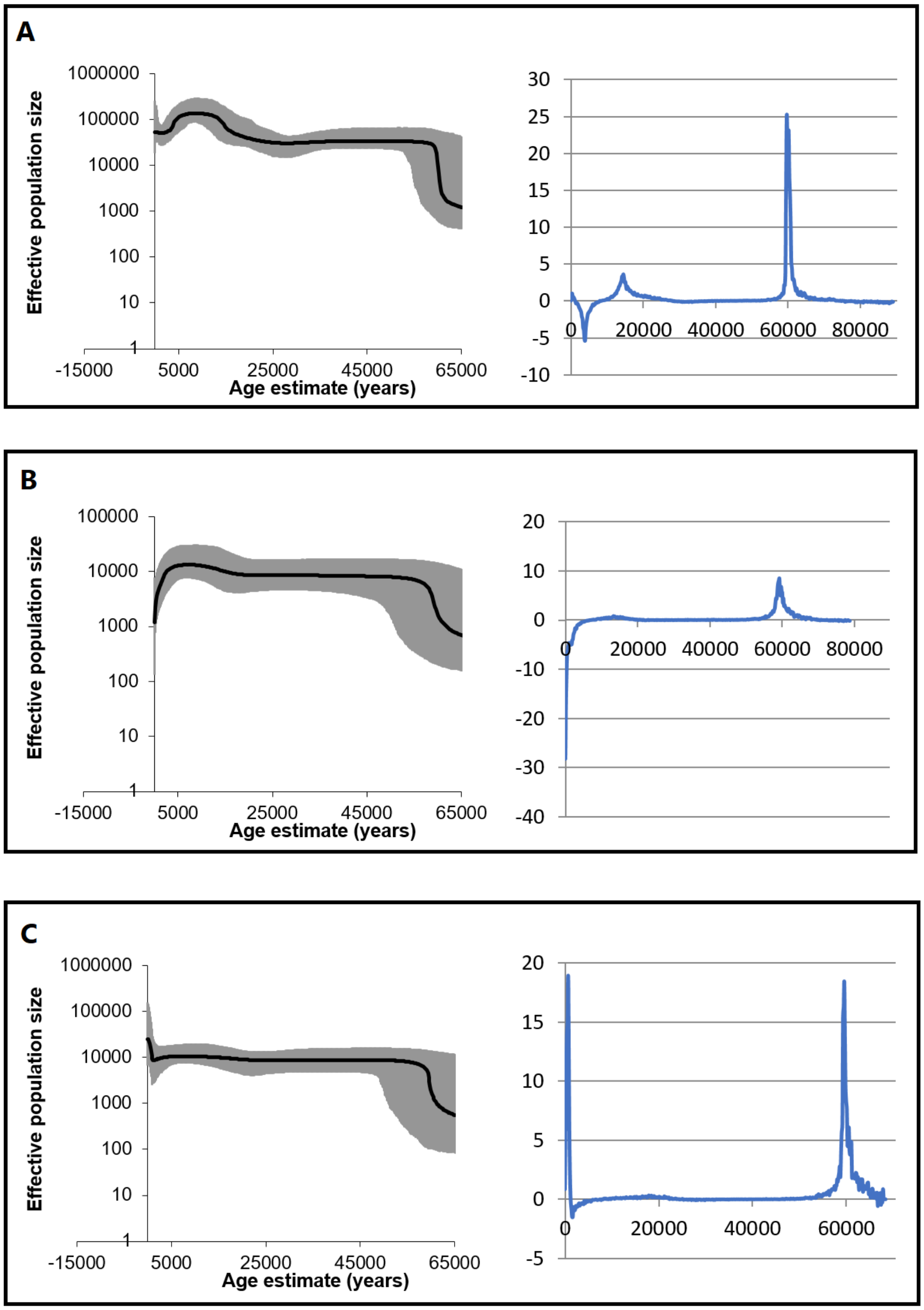

Figure S1. Bayesian skyline plots for New Guinea and the western Pacific. BSPs for (**A**), Eastern New Guinea, (**B**) the Bismarck Archipelago and (**C**) the Solomon Islands. The left-hand plot shows the effective population size against time; the right-hand plot shows the change in effective population size over time. We performed the Eastern New Guinea plot on three random subsets of the data, of which one is shown, as well as the highlands only and the whole dataset partitioned into west and east. The first three plots were similar, with the main increment at ~60–63 ka; the latter both gave slightly reduced expansion time of ~56 ka. The three randomly selected analyses all gave a second peak at ~15–18 ka that is present in the other Eastern New Guinea analyses but missing in both the Bismarcks and Solomons. When we separated out the

Eastern New Guinea highlands, the peak was at ~59 ka. We also performed the Bismarcks and Solomons together, with a similar increment to the separate runs at ~59–60 ka. Vanuatu gave an increment at ~58 ka and a more recent peak at ~15 ka, like that in New Guinea, which may reflect substantial recent New Guinean ancestry.

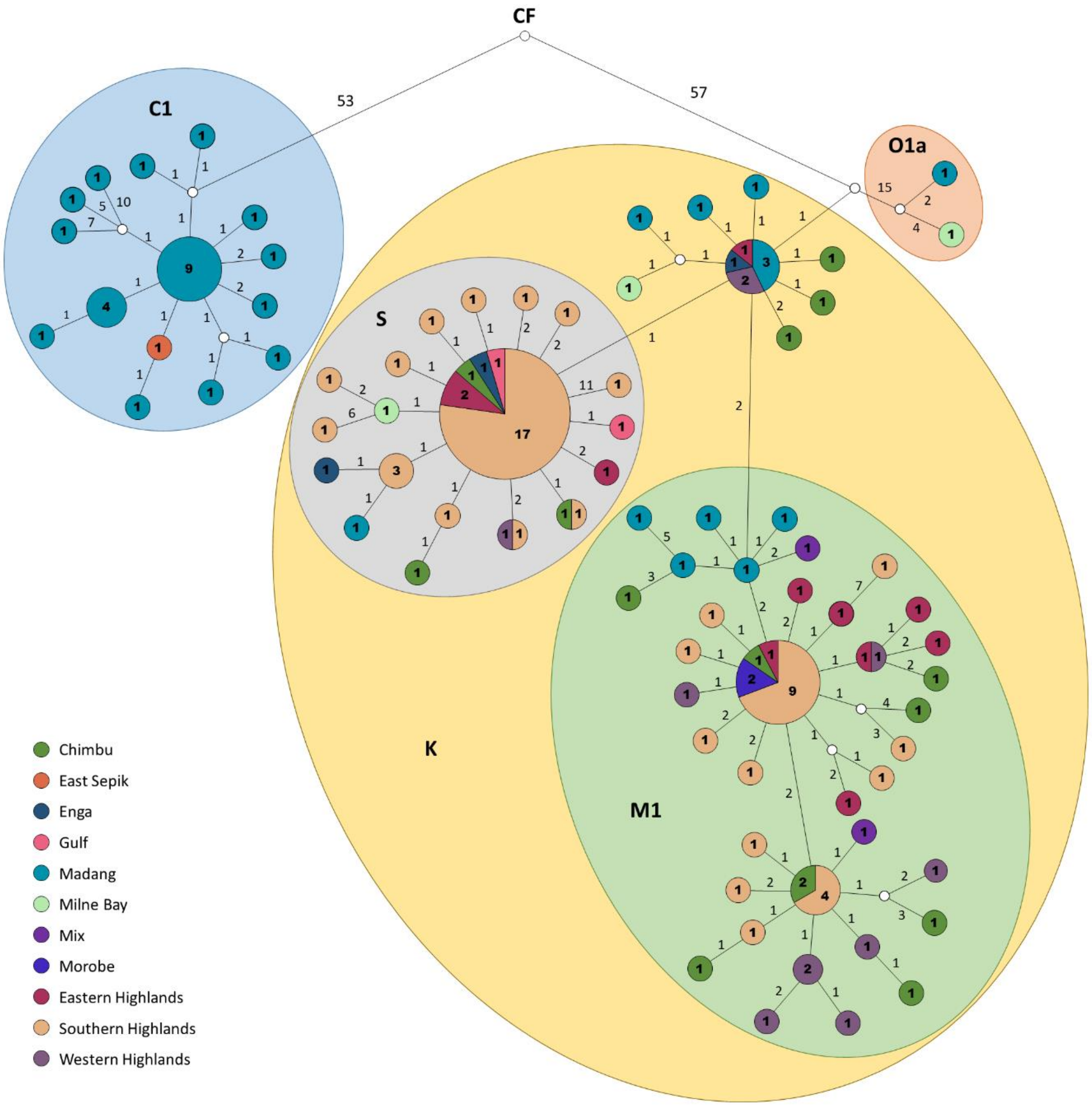

Figure S2. Y-chromosome SNP tree. Tree generated using the reduced-median algorithm of Network 10 from the Eastern New Guinea data of Bergström et al. (*23*). Haplogroup and sub-haplogroup affiliations are shown. The circles indicate SNP haplotypes, numbers within the circles indicate the number of samples with each haplotype, the links indicate branches of the tree with mutations separating the haplotypes, and the numbers on the links indicate the number of mutations on each branch.

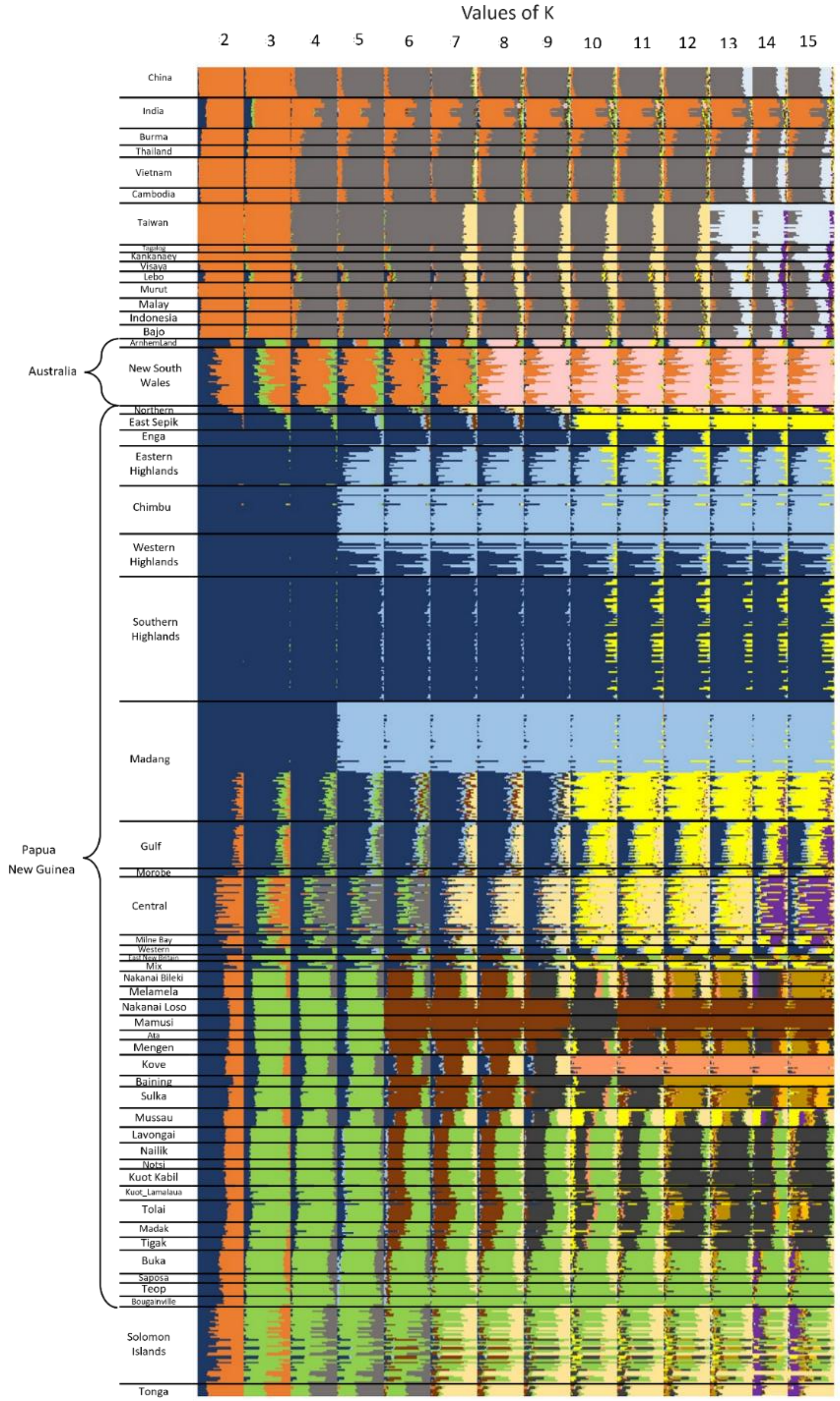

Figure S3. ADMIXTURE plots of genome-wide data. Data from analysis 1 (including more Aboriginal Australians) for K=2 to K=15, using 37,216 SNPs. Lowest CV error K=9.

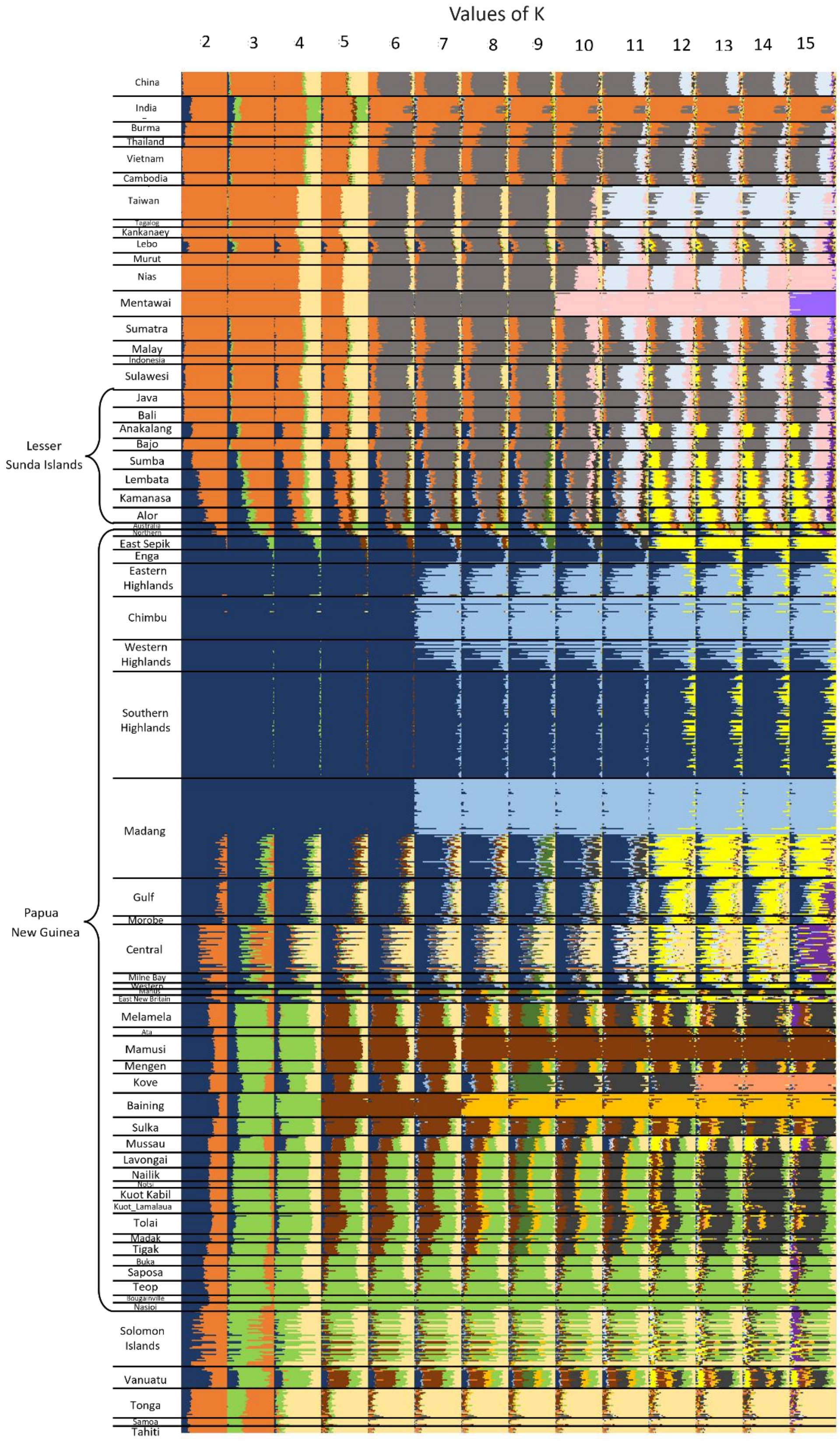

Figure S4. ADMIXTURE plots of genome-wide data. Data from analysis 2 (including more Indonesians and Pacific islanders) for *K*=2 to *K*=15, using 21,328 SNPs. Lowest CV error *K*=12.

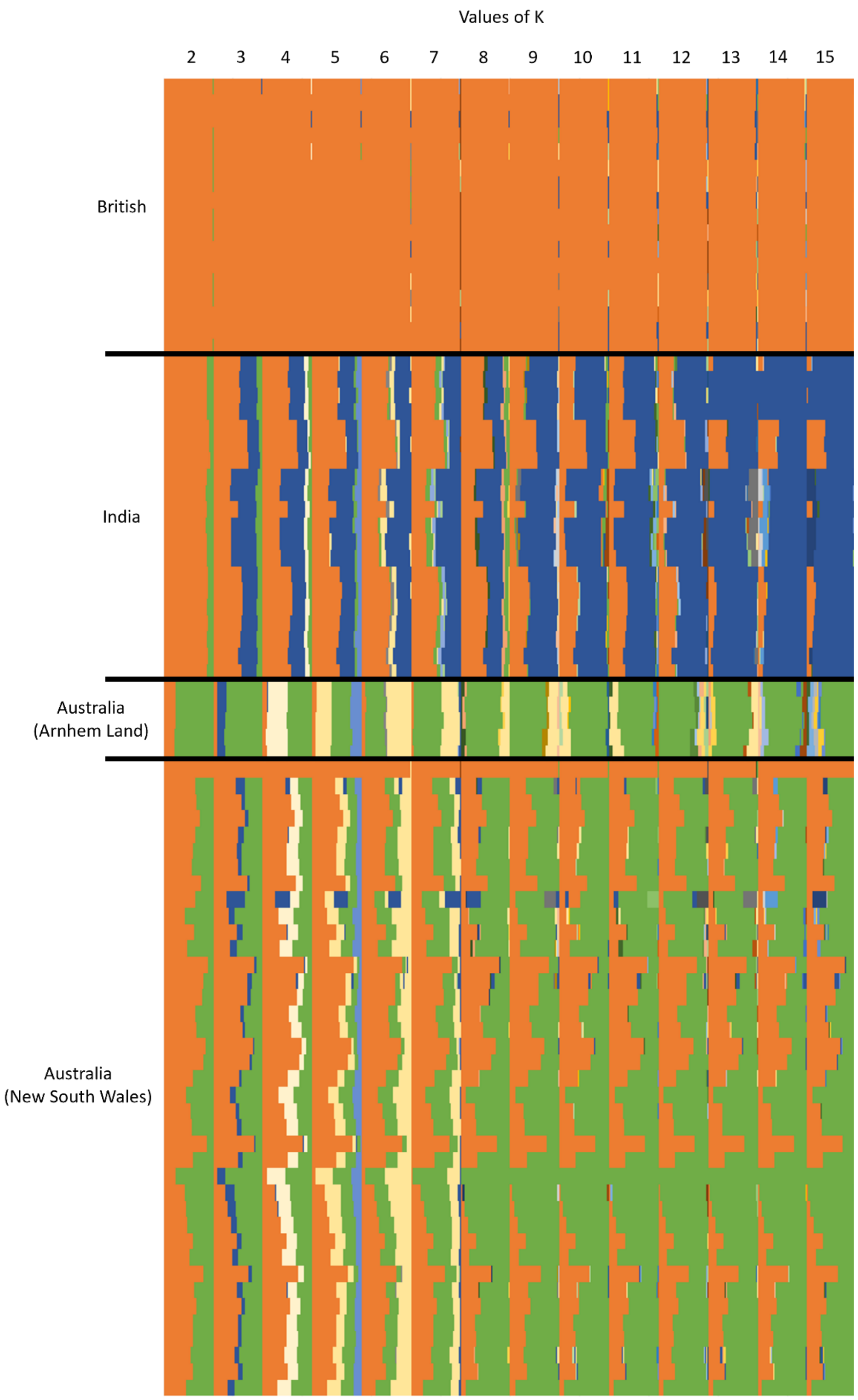

Figure S5. **ADMIXTURE plots of Aboriginal Australians and Indians genome-wide data.** Data from analysis 1 including British, indicating European ancestry in Aboriginal Australians, for *K*=2 to *K*=15, using 23,088 SNPs.

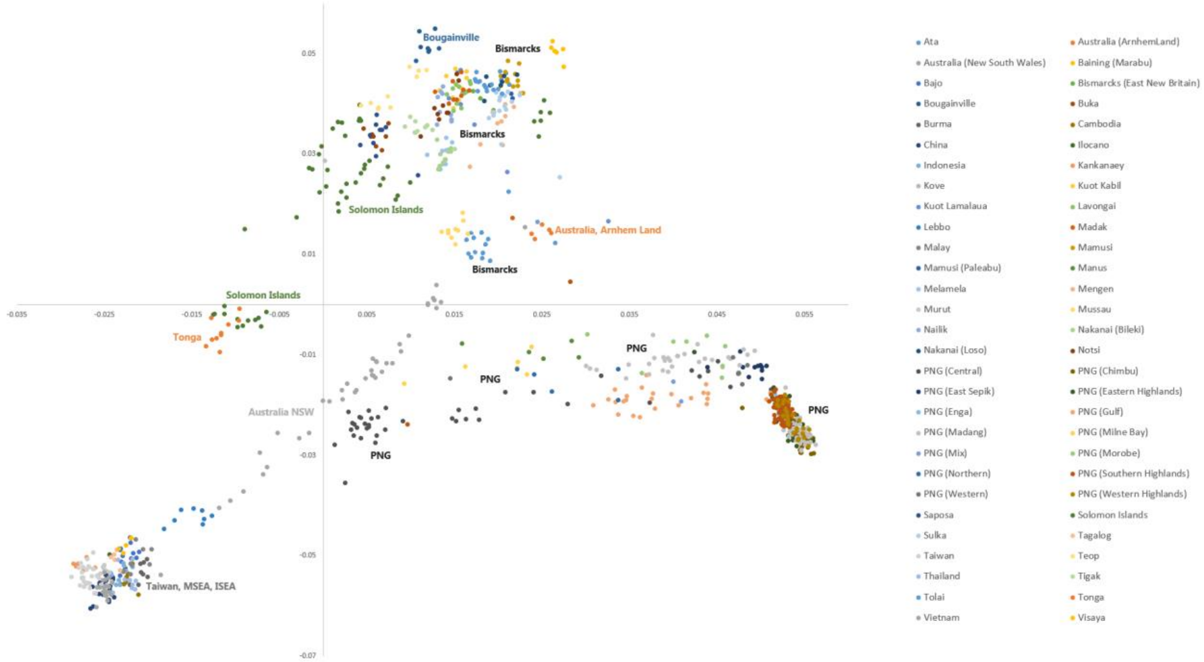

Figure S6. Plot of the first two principal components of PCA of genome-wide data. Data from analysis 1 (including more Aboriginal Australians), using 37,216 SNPs.

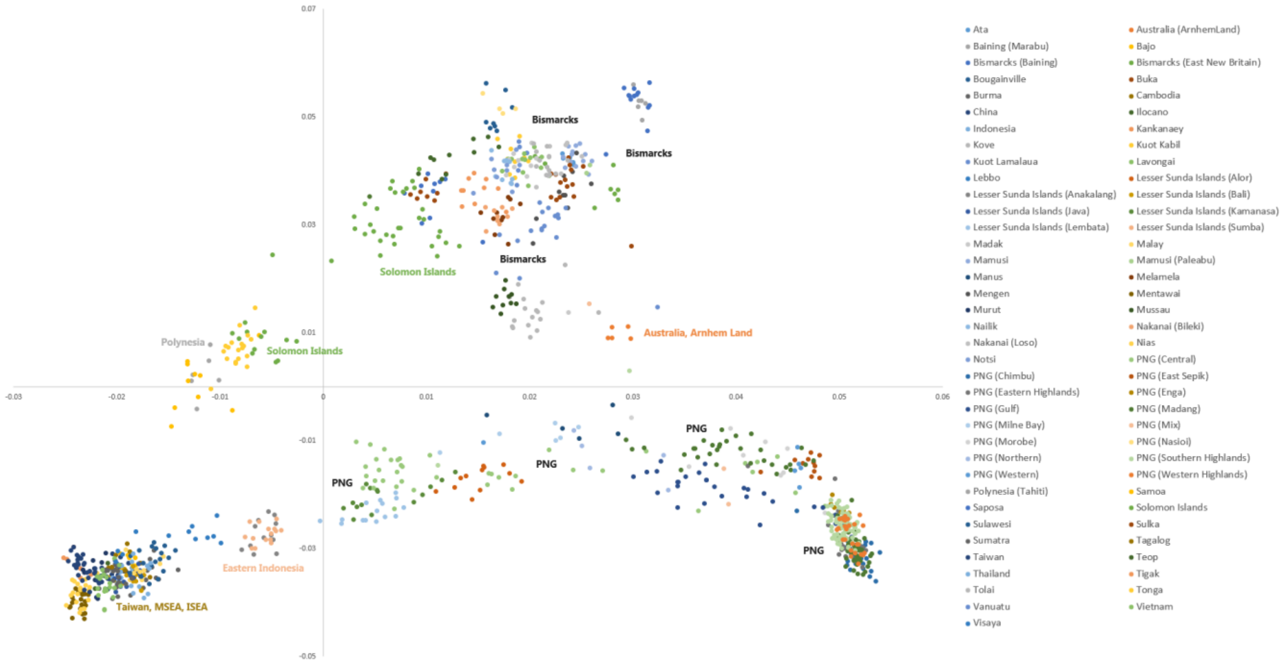

Figure S7. Plot of the first two principal components of PCA of genome-wide data. Data from analysis 2 (weighted to more Indonesians and Pacific islanders), using 21,328 SNPs.

(A)

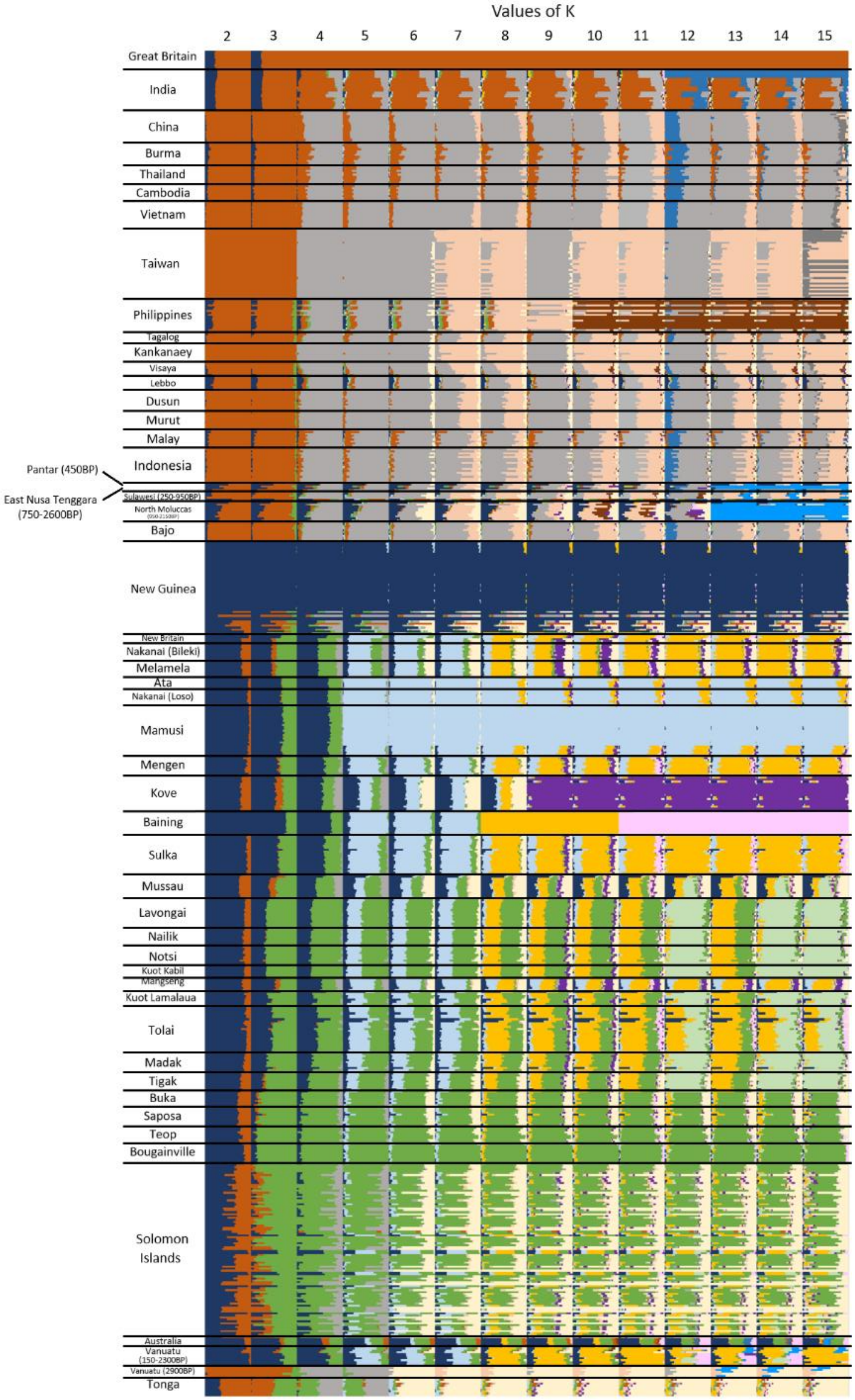
Values of K
2
3
4
5
6
7
8
9
10
11
12
13
14
15
Great Britain
India
China
Burma
Thailand
Cambodia
Vietnam
Taiwan
Philippines
Tagalog
Kankanaey
Visaya
Lebbo
Dusun
Murut
Malay
Indonesia
Pantar (450BP)
East Nusa Tenggara (750-2600BP)
Sulawesi (250-950BP)
North Moluccas
Bajo
New Guinea
New Britain
Nakanai (Bileki)
Melamela
Ata
Nakanai (Loso)
Mamusi
Mengen
Kove
Baining
Sulka
Mussau
Lavongai
Nailik
Notsi
Kuot Kabil
Mangseng
Kuot Lamalaua
Tolai
Madak
Tigak
Buka
Saposa
Teop
Bougainville
Solomon Islands
Australia
Vanuatu (150-2300BP)
Vanuatu (2900BP)
Tonga

(B)

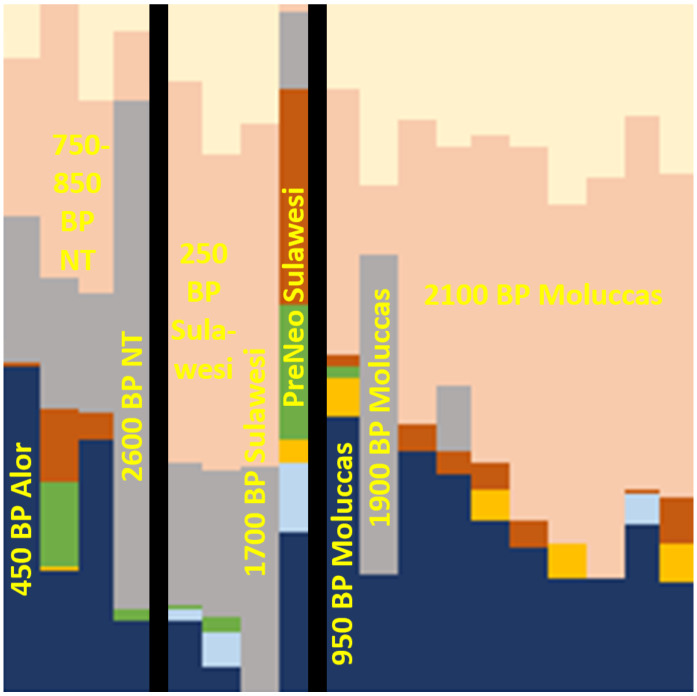

Figure S8. ADMIXTURE plots with aDNA genomes included. (A) ADMIXTURE plots including archaeological remains from Sulawesi, Moluccas and Lesser Sundas (*42*), including a pre-Neolithic Toalean young female from southwest Sulawesi (*41*) and an elderly female dating to 256–393 AD from Gua Talimbue, southeast Sulawesi (this study), as well as archaeological remains from Vanuatu dated from 150 to 2900 years ago (*26*), alongside modern data of Pugach et al. (*25*) and Skoglund et al. (*28*) for *K*=2 to *K*=15, using 53,161 SNPs. Lowest CV error = 8. The Vanuatu data show the shift from a Polynesian-like profile to largely Bismarck-like profile over the first few hundred years. **(B)** Details from plots in A showing Wallacean aDNA genomes.

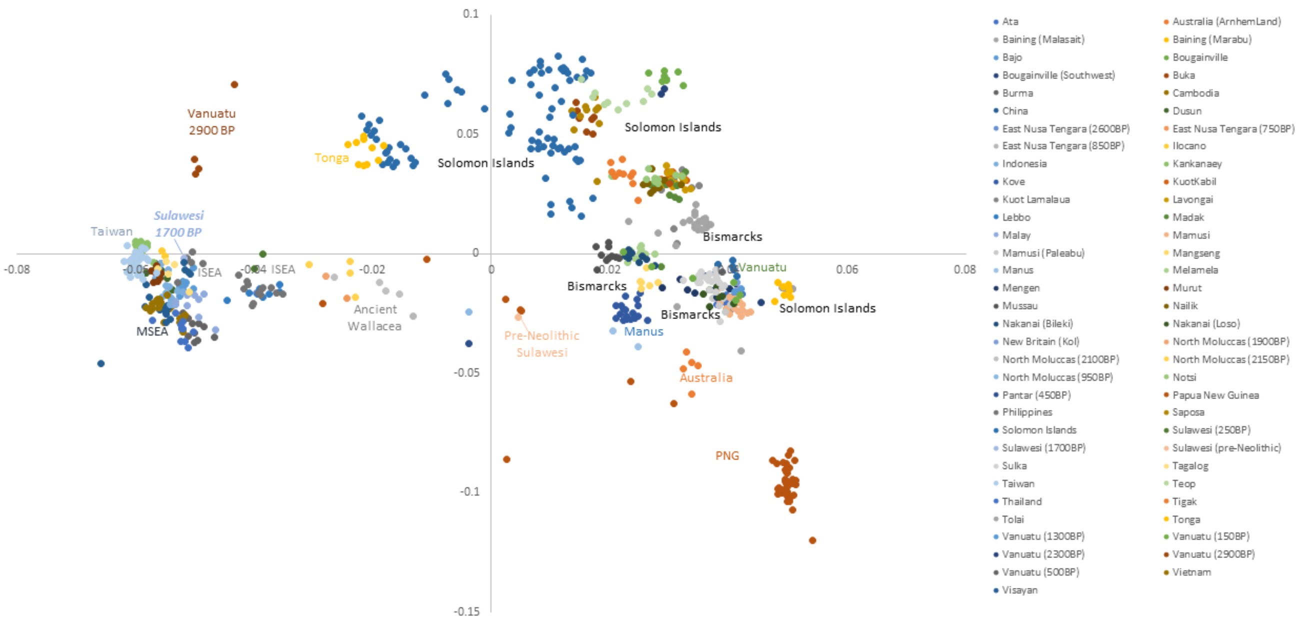

Figure S9. Plot of the first two principal components of PCA with aDNA genomes included. Plot includes data of Pugach et al. (*25*) and Skoglund et al. (*28*), using 53,161 SNPs, with aDNA genomes projected, including the 1.7 ka Iron Age sample from Gua Talimbue, Sulawesi.

(A)

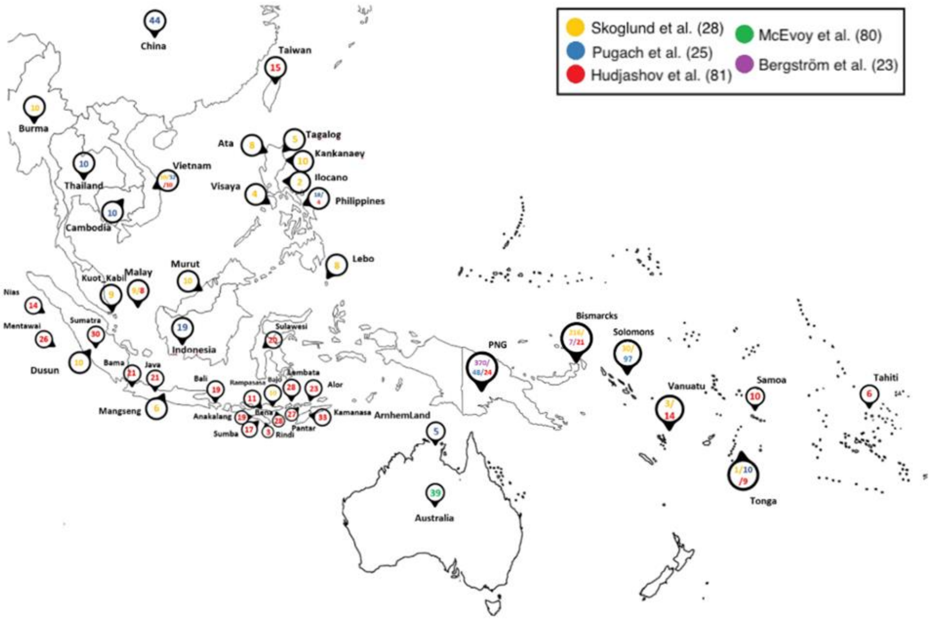

(B)

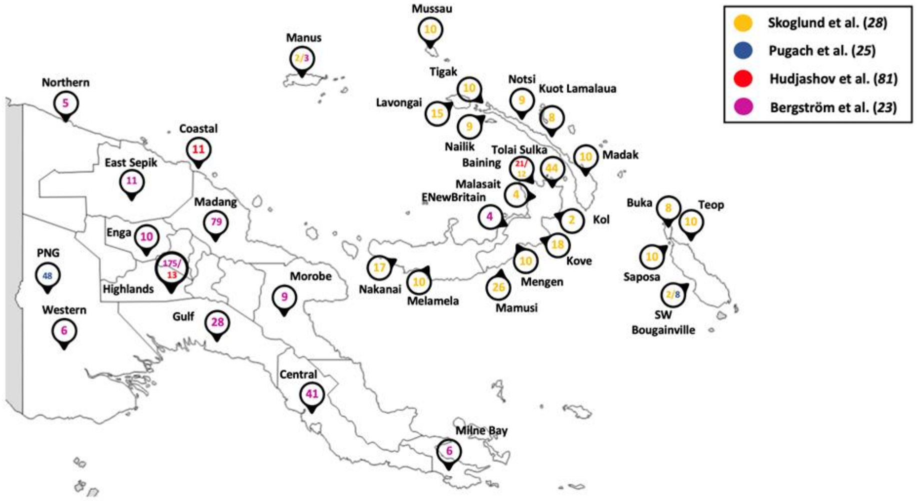

Figure S10. Data used for genome-wide analyses. (A) Numbers in each balloon record the sample size used for the location indicated, with the colour of numbers matching publications key (see also Table S4). **(B)** Details for New Guinea region.

	Bergström et al. (*23*)	McEvoy et al. (*80*)	Pugach et al. (*25*)	Hudjashov et al. (*81*)	Skoglund et al. (*28*)	Totals
**China**			44			44
**Mainland Southeast Asia**			20	10	29	59
**Taiwan**				15		15
**Philippines**			18	4	37	59
**Lesser Sunda Islands**				250	35	285
**Murut**					10	10
**Indonesia**			19			19
**Sulawesi**				20		20
**Papua New Guinea**	370		48	24		442
**Bismarcks**	3			21	216	240
**Solomons**			97		30	127
**Vanuatu**				14	3	17
**Samoa**				10		10
**Tonga**			10	9	1	20
**Tahiti**				6		6
**Australia**		39	5			44

Table S4. Published samples and data used in this study.

Lineages indigenous to northern or southern Sahul	Inferred route from Sunda	Total	Fraction
Northern Sahul (New Guinea, Bismarcks, Solomons)			
P	Northern	334	0.342
Q	Northern	424	0.434
M29	Northern	16	0.016
M28	Northern	50	0.051
M27	Northern	144	0.148
M25	Northern	7	0.007
R14 (orphan lineage)	*Northern*	*1*	*0.001*
Total excluding postglacial		976	1.000
E, M7c3c, M73, N13, S2	Postglacial	74	0.070
New Guinea/Near Oceania total		1050	
Southern Sahul (Australia, including Tasmania)			
P	Northern	130	0.362
M42	Southern	67	0.187
S	Southern	93	0.259
O	Southern	52	0.145
N13	Southern	9	0.025
M14, M15, M16	Southern	5	0.014
R12	Southern	3	0.008
Total excluding postglacial		359	
Q	Postglacial	10	0.028
Australia/Tasmania total		369	
Sahul/Near Oceania total		1419	

Table S5. Lineage fractions in northern and southern Sahul.

Region	Dating techniques						
	Radiocarbon	Luminescence	Radiocarbon and luminescence	Radiocarbon and U-series	U-series	Luminescence, U-series and ESR	Radiocarbon, luminescence, U-series and ESR
**Sunda**	>43 ka			>46	40–52 ka	63–73 ka	
**Wallacea**	>43 ka<br>42 ka<br>42.3 ka		>44 ka		>47 ka<br>>36 ka<br>>39 ka<br>>40 ka<br>>45.5 ka<br>>32 ka<br>>50 ka<br>>53.5 ka		
**Sahul**	43–49 ka<br>45.6–52.2 ka<br>44–51 ka<br>>47.8 ka	47–61 ka<br>47.2–51.4 ka<br>45–50 ka<br>46–50 ka	65–52.7 ka<br>60.3–53.4 ka<br>44.6–46.4 ka<br>46–51 ka<br>49 ka				48 ka

Table S6. Summary of estimated ages for initial occupation, and method used to date each site, by region.

Supplementary Excel File

Data S1. Maximum-parsimony trees of macrohaplogroups M and N. They encompass respectively 1699 and 757 complete mitochondrial DNA sequences (listed in Tables S1 and S2), respectively. Mutations are shown on the branches (relative to rCRS), they are transitions unless the base change is explicitly indicated; suffixes indicate: transversions (to A, G, C, or T) and reversions (@). Heteroplasmies are indicated with the prefix "het", deletions with the suffix "d" and insertions with the suffix "ins" followed by the inserted base(s), recurrent mutations are underlined. The mutations 309insC(C), 315insC, AC indels at 515–522, 16182C, 16183C, 16193ins1C(C) and 16519T were not considered for phylogenetic reconstruction and were therefore excluded from the trees. Accession and ID numbers of mitogenomes correspond to those in Tables S1 and S2. Samples are coloured according to their geographic origin as shown in the legend (top left). Nodes are marked in red and maximum-likelihood and Bayesian age estimates, calculated with the mutation rate of Soares et al. (*33*), are shown with orange and yellow backgrounds, respectively. These, alongside ages obtained with other methods/rates, are listed in full in Table S3.

Table S1. Geographic origin and haplogroup affiliation of newly sequenced mitogenomes. Includes the 973 newly sequenced mitogenomes used to build the phylogenetic trees in Data. S1.

Table S2. Geographic origin and haplogroup affiliation of previously published mitogenomes. Includes the 1483 previously published modern and ancient mitogenomes used to build phylogenetic trees in Data S1.

Table S3. Age estimates of the haplogroups and sub-haplogroups included in the phylogenetic trees in Data S1. Age estimates are in ka and were calculated with maximum-likelihood, Bayesian estimates and ρ statistics, with both Soares et al. (*33*) and Fu et al. (*44*) mutation rates.

REFERENCES AND NOTES

1. D. Bulbeck, Where river meets sea: A parsimonious model for *Homo sapiens* colonization of the Indian Ocean rim and Sahul. *Curr. Anthropol.* **48**, 315–321 (2007).

2. P. Hiscock, *Archaeology of Ancient Australia* (Routledge, 2007).

3. E. K. Kuijjer, I. D. Haigh, R. Marsh, R. H. Farr, Changing tidal dynamics and the role of the marine environment in the maritime migration to Sahul. *PaleoAnthropology* **1**, 134–148 (2022).

4. M. I. Bird, S. A. Condie, S. O'Connor, D. O'Grady, C. Reepmeyer, S. Ulm, M. Zega, F. Saltre, C. J. A. Bradshaw, Early human settlement of Sahul was not an accident. *Sci. Rep.* **9**, 8220 (2019).

5. C. Clarkson, Z. Jacobs, B. Marwick, R. Fullagar, L. Wallis, M. Smith, R. G. Roberts, E. Hayes, K. Lowe, X. Carah, S. A. Florin, J. McNeil, D. Cox, L. J. Arnold, Q. Hua, J. Huntley, H. E. A. Brand, T. Manne, A. Fairbairn, J. Shulmeister, L. Lyle, M. Salinas, M. Page, K. Connell, G. Park, K. Norman, T. Murphy, C. Pardoe, Human occupation of northern Australia by 65,000 years ago. *Nature* **547**, 306–310 (2017).

6. K. E. Westaway, J. Louys, R. D. Awe, M. J. Morwood, G. J. Price, J. X. Zhao, M. Aubert, R. Joannes-Boyau, T. M. Smith, M. M. Skinner, T. Compton, R. M. Bailey, G. D. van den Bergh, J. de Vos, A. W. G. Pike, C. Stringer, E. W. Saptomo, Y. Rizal, J. Zaim, W. D. Santoso, A. Trihascaryo, L. Kinsley, B. Sulistyanto, An early modern human presence in Sumatra 73,000–63,000 years ago. *Nature* **548**, 322–325 (2017).

7. P. Veth, Breaking through the radiocarbon barrier: Madjedbebe and the new chronology for Aboriginal occupation of Australia. *Aust. Archaeol.* **83**, 165–167 (2017).

8. S. E. Freidline, K. E. Westaway, R. Joannes-Boyau, P. Duringer, J.-L. Ponche, M. W. Morley, V. C. Hernandez, M. S. McAllister-Hayward, H. McColl, C. Zanolli, P. Gunz, I. Bergmann, P. Sichanthongtip, D. Sihanam, S. Boualaphane, T. Luangkhoth, V. Souksavatdy, A. Dosseto, Q. Boesch, E. Patole-Edoumba, F. Aubaile, F. Crozier, E. Suzzoni, S. Frangeul, N. Bourgon, A. Zachwieja, T. E. Dunn, A.-M. Bacon, J.-J. Hublin, L. Shackelford, F. Demeter, Early presence

of *Homo sapiens* in Southeast Asia by 86-68 kyr at Tam Pa Ling, Northern Laos. *Nat. Commun.* **14**, 3193 (2023).

9. J. Allen, J. F. O'Connell, A different paradigm for the initial colonisation of Sahul. *Archaeol. Ocean.* **55**, 1–14 (2020).

10. S. M. van Holst Pellekaan, M. Ingman, J. Roberts-Thomson, R. M. Harding, Mitochondrial genomics identifies major haplogroups in Aboriginal Australians. *Am. J. Phys. Anthropol.* **131**, 282–294 (2006).

11. G. Hudjashov, T. Kivisild, P. A. Underhill, P. Endicott, J. J. Sanchez, A. A. Lin, P. Shen, P. Oefner, C. Renfrew, R. Villems, P. Forster, Revealing the prehistoric settlement of Australia by Y chromosome and mtDNA analysis. *Proc. Natl. Acad. Sci. U.S.A.* **104**, 8726–8730 (2007).

12. N. Nagle, M. van Oven, S. Wilcox, S. V. Pellekaan, C. Tyler-Smith, Y. L. Xue, K. N. Ballantyne, L. Wilcox, L. Papac, K. Cooke, R. A. H. van Oorschot, P. McAllister, L. Williams, M. Kayser, R. J. Mitchell, Genographic Consortium, Aboriginal Australian mitochondrial genome variation—An increased understanding of population antiquity and diversity. *Sci. Rep.* **7**, 43741 (2017).

13. N. Nagle, K. N. Ballantyne, M. van Oven, C. Tyler-Smith, Y. L. Xue, S. Wilcox, L. Wilcox, R. Turkalov, R. A. H. van Oorschot, S. V. Pellekaan, T. G. Schurr, P. McAllister, L. Williams, M. Kayser, R. J. Mitchell, Genographic Consortium, Mitochondrial DNA diversity of present-day Aboriginal Australians and implications for human evolution in Oceania. *J. Hum. Genet.* **62**, 343–353 (2017).

14. R. Tobler, A. Rohrlach, J. Soubrier, P. Bover, B. Llamas, J. Tuke, N. Bean, A. Abdullah-Highfold, S. Agius, A. O'Donoghue, I. O'Loughlin, P. Sutton, F. Zilio, K. Walshe, A. N. Williams, C. S. M. Turney, M. Williams, S. M. Richards, R. J. Mitchell, E. Kowal, J. R. Stephen, L. Williams, W. Haak, A. Cooper, Aboriginal mitogenomes reveal 50,000 years of regionalism in Australia. *Nature* **544**, 180–184 (2017).

15. N. Pedro, N. Brucato, V. Fernandes, M. Andre, L. Saag, W. Pomat, C. Besse, A. Boland, J. F. Deleuze, C. Clarkson, H. Sudoyo, M. Metspalu, M. Stoneking, M. P. Cox, M. Leavesley, L. Pereira, F. X. Ricaut, Papuan mitochondrial genomes and the settlement of Sahul. *J. Hum. Genet.* **65**, 875–887 (2020).

16. A. Bergström, N. Nagle, Y. Chen, S. McCarthy, M. O. Pollard, Q. Ayub, S. Wilcox, L. Wilcox, R. A. H. van Oorschot, P. McAllister, L. Williams, Y. Xue, R. J. Mitchell, C. Tyler-Smith, Deep roots for Aboriginal Australian Y chromosomes. *Curr.Biol.* **26**, 809–813 (2016).

17. J. C. Teixeira, A. Cooper, Using hominin introgression to trace modern human dispersals. *Proc. Natl. Acad. Sci. U.S.A.* **116**, 15327–15332 (2019).

18. J. F. O'Connell, J. Allen, M. A. J. Williams, A. N. Williams, C. S. M. Turney, N. A. Spooner, J. Kamminga, G. Brown, A. Cooper, When did *Homo sapiens* first reach Southeast Asia and Sahul? *Proc. Natl. Acad. Sci. U.S.A.* **115**, 8482–8490 (2018).

19. A.-S. Malaspinas, M. C. Westaway, C. Muller, V. C. Sousa, O. Lao, I. Alves, A. Bergström, G. Athanasiadis, J. Y. Cheng, J. E. Crawford, T. H. Heupink, E. Macholdt, S. Peischl, S. Rasmussen, S. Schiffels, S. Subramanian, J. L. Wright, A. Albrechtsen, C. Barbieri, I. Dupanloup, A. Eriksson, A. Margaryan, I. Moltke, I. Pugach, T. S. Korneliussen, I. P. Levkivskyi, J. V. Moreno-Mayar, S. Ni, F. Racimo, M. Sikora, Y. Xue, F. A. Aghakhanian, N. Brucato, S. Brunak, P. F. Campos, W. Clark, S. Ellingvåg, G. Fourmile, P. Gerbault, D. Injie, G. Koki, M. Leavesley, B. Logan, A. Lynch, E. A. Matisoo-Smith, P. J. McAllister, A. J. Mentzer, M. Metspalu, A. B. Migliano, L. Murgha, M. E. Phipps, W. Pomat, D. Reynolds, F.-X. Ricaut, P. Siba, M. G. Thomas, T. Wales, C. M. Wall, S. J. Oppenheimer, C. Tyler-Smith, R. Durbin, J. Dortch, A. Manica, M. H. Schierup, R. A. Foley, M. M. Lahr, C. Bowern, J. D. Wall, T. Mailund, M. Stoneking, R. Nielsen, M. S. Sandhu, L. Excoffier, D. M. Lambert, E. Willerslev, A genomic history of Aboriginal Australia. *Nature* **538**, 207–214 (2016).

20. M. Rasmussen, X. Guo, Y. Wang, K. E. Lohmueller, S. Rasmussen, A. Albrechtsen, L. Skotte, S. Lindgreen, M. Metspalu, T. Jombart, T. Kivisild, W. Zhai, A. Eriksson, A. Manica, L. Orlando, F. M. De La Vega, S. Tridico, E. Metspalu, K. Nielsen, M. C. Ávila-Arcos, J. V. Moreno-Mayar, C. Muller, J. Dortch, M. T. P. Gilbert, O. Lund, A. Wesolowska, M. Karmin,

L. A. Weinert, B. Wang, J. Li, S. Tai, F. Xiao, T. Hanihara, G. van Driem, A. Jha, F.-X. Ricaut, P. de Knijff, A. B. Migliano, I. G. Romero, K. Kristiansen, D. M. Lambert, S. Brunak, P. Forster, B. Brinkmann, O. Nehlich, M. Bunce, M. Richards, R. Gupta, C. Bustamante, A. Krogh, R. A. Foley, M. M. Lahr, F. Balloux, T. Sicheritz-Pontén, R. Villems, R. Nielsen, J. Wang, E. Willerslev, An Aboriginal Australian genome reveals separate human dispersals into Asia. *Science* **334**, 94–98 (2011).

21. J. D. Wall, Inferring human demographic histories of non-African populations from patterns of allele sharing. *Am. J. Hum. Genet.* **100**, 766–772 (2017).

22. N. Brucato, M. Andre, R. Tsang, L. Saag, J. Kariwiga, K. Sesuki, T. Beni, W. Pomat, J. Muke, V. Meyer, A. Boland, J. F. Deleuze, H. Sudoyo, M. Mondal, L. Pagani, I. G. Romero, M. Metspalu, M. P. Cox, M. Leavesley, F. X. Ricaut, Papua New Guinean genomes reveal the complex settlement of North Sahul. *Mol. Biol. Evol.* **38**, 5107–5121 (2021).

23. A. Bergström, S. J. Oppenheimer, A. J. Mentzer, K. Auckland, K. Robson, R. Attenborough, M. P. Alpers, G. Koki, W. Pomat, P. Siba, Y. L. Xue, M. S. Sandhu, C. Tyler-Smith, A Neolithic expansion, but strong genetic structure, in the independent history of New Guinea. *Science* **357**, 1160–1163 (2017).

24. G. A. Purnomo, K. J. Mitchell, S. O'Connor, S. Kealy, L. Taufik, S. Schiller, A. Rohrlach, A. Cooper, B. Llamas, H. Sudoyo, J. C. Teixeira, R. Tobler, Mitogenomes reveal two major influxes of Papuan ancestry across Wallacea following the Last Glacial Maximum and Austronesian contact. *Genes* **12**, 965 (2021).

25. I. Pugach, A. T. Duggan, D. A. Merriwether, F. R. Friedlaender, J. S. Friedlaender, M. Stoneking, The gateway from Near into Remote Oceania: New insights from genome-wide data. *Mol. Biol. Evol.* **35**, 871–886 (2018).

26. M. Lipson, P. Skoglund, M. Spriggs, F. Valentin, S. Bedford, R. Shing, H. Buckley, I. Phillip, G. K. Ward, S. Mallick, N. Rohland, N. Broomandkhoshbacht, O. Cheronet, M. Ferry, T. K. Harper, M. Michel, J. Oppenheimer, K. Sirak, K. Stewardson, K. Auckland, A. V. S. Hill, K. Maitland, S. J. Oppenheimer, T. Parks, K. Robson, T. N. Williams, D. J. Kennett, A. J.

Mentzer, R. Pinhasi, D. Reich, Population turnover in Remote Oceania shortly after initial settlement. *Curr. Biol.* **28**, 1157–1165.e7 (2018).

27. C. Posth, K. Nagele, H. Colleran, F. Valentin, S. Bedford, K. W. Kami, R. Shing, H. Buckley, R. Kinaston, M. Walworth, G. R. Clark, C. Reepmeyer, J. Flexner, T. Maric, J. Moser, J. Gresky, L. Kiko, K. J. Robson, K. Auckland, S. J. Oppenheimer, A. V. S. Hill, A. J. Mentzer, J. Zech, F. Petchey, P. Roberts, C. Jeong, R. D. Gray, J. Krause, A. Powell, Language continuity despite population replacement in Remote Oceania. *Nat. Ecol. Evol.* **2**, 731–740 (2018).

28. P. Skoglund, C. Posth, K. Sirak, M. Spriggs, F. Valentin, S. Bedford, G. R. Clark, C. Reepmeyer, F. Petchey, D. Fernandes, Q. Fu, E. Harney, M. Lipson, S. Mallick, M. Novak, N. Rohland, K. Stewardson, S. Abdullah, M. P. Cox, F. R. Friedlaender, J. S. Friedlaender, T. Kivisild, G. Koki, P. Kusuma, D. A. Merriwether, F.-X. Ricaut, J. T. S. Wee, N. Patterson, J. Krause, R. Pinhasi, D. Reich, Genomic insights into the peopling of the Southwest Pacific. *Nature* **538**, 510–513 (2016).

29. M. Lipson, M. Spriggs, F. Valentin, S. Bedford, R. Shing, W. Zinger, H. Buckley, F. Petchey, R. Matanik, O. Cheronet, N. Rohland, R. Pinhasi, D. Reich, Three phases of ancient migration shaped the ancestry of human populations in Vanuatu. *Curr. Biol.* **30**, 4846–4856.e6 (2020).

30. S. O'Connor, P. Hiscock, in *The Oxford Handbook of Prehistoric Oceania*, E. E. Cochrane, T. L. Hunt, Eds. (Oxford Univ. Press, 2018).

31. A. S. Malaspinas, M. C. Westaway, S. Subramanian, J. Wright, L. David, E. Willerslev, The genomic history of the first Australians. *Am. J. Phys. Anthropol.* **162**, 407–407 (2017).

32. S. Mallick, H. Li, M. Lipson, I. Mathieson, M. Gymrek, F. Racimo, M. Zhao, N. Chennagiri, S. Nordenfelt, A. Tandon, P. Skoglund, I. Lazaridis, S. Sankararaman, Q. Fu, N. Rohland, G. Renaud, Y. Erlich, T. Willems, C. Gallo, J. P. Spence, Y. S. Song, G. Poletti, F. Balloux, G. van Driem, P. de Knijff, I. G. Romero, A. R. Jha, D. M. Behar, C. M. Bravi, C. Capelli, T. Hervig, A. Moreno-Estrada, O. L. Posukh, E. Balanovska, O. Balanovsky, S. Karachanak-Yankova, H. Sahakyan, D. Toncheva, L. Yepiskoposyan, C. Tyler-Smith, Y. Xue, M. S.

Abdullah, A. Ruiz-Linares, C. M. Beall, A. Di Rienzo, C. Jeong, E. B. Starikovskaya, E. Metspalu, J. Parik, R. Villems, B. M. Henn, U. Hodoglugil, R. Mahley, A. Sajantila, G. Stamatoyannopoulos, J. T. S. Wee, R. Khusainova, E. Khusnutdinova, S. Litvinov, G. Ayodo, D. Comas, M. F. Hammer, T. Kivisild, W. Klitz, C. A. Winkler, D. Labuda, M. Bamshad, L. B. Jorde, S. A. Tishkoff, W. S. Watkins, M. Metspalu, S. Dryomov, R. Sukernik, L. Singh, K. Thangaraj, S. Paabo, J. Kelso, N. Patterson, D. Reich, The Simons Genome Diversity Project: 300 genomes from 142 diverse populations. *Nature* **538**, 201–206 (2016).

33. P. Soares, L. Ermini, N. Thomson, M. Mormina, T. Rito, A. Röhl, A. Salas, S. Oppenheimer, V. Macaulay, M. B. Richards, Correcting for purifying selection: An improved human mitochondrial molecular clock. *Am. J. Hum. Genet.* **84**, 740–759 (2009).

34. G. D. Poznik, Y. Xue, F. L. Mendez, T. F. Willems, A. Massaia, M. A. Wilson Sayres, Q. Ayub, S. A. McCarthy, A. Narechania, S. Kashin, Y. Chen, R. Banerjee, J. L. Rodriguez-Flores, M. Cerezo, H. Shao, M. Gymrek, A. Malhotra, S. Louzada, R. Desalle, G. R. S. Ritchie, E. Cerveira, T. W. Fitzgerald, E. Garrison, A. Marcketta, D. Mittelman, M. Romanovitch, C. Zhang, X. Zheng-Bradley, G. R. Abecasis, S. A. McCarroll, P. Flicek, P. A. Underhill, L. Coin, D. R. Zerbino, F. Yang, C. Lee, L. Clarke, A. Auton, Y. Erlich, R. E. Handsaker, 1000 Genomes Project Consortium, C. D. Bustamante, C. Tyler-Smith, Punctuated bursts in human male demography inferred from 1,244 worldwide Y-chromosome sequences. *Nat. Genet.* **48**, 593–599 (2016).

35. P. Mellars, K. C. Gori, M. Carr, P. A. Soares, M. B. Richards, Genetic and archaeological perspectives on the initial modern human colonization of southern Asia. *Proc. Natl. Acad. Sci. U.S.A.* **110**, 10699–10704 (2013).

36. V. Macaulay, C. Hill, A. Achilli, C. Rengo, D. Clarke, W. Meehan, J. Blackburn, O. Semino, R. Scozzari, F. Cruciani, A. Taha, N. K. Shaari, J. M. Raja, P. Ismail, Z. Zainuddin, W. Goodwin, D. Bulbeck, H.-J. Bandelt, S. Oppenheimer, A. Torroni, M. Richards, Single, rapid coastal settlement of Asia revealed by analysis of complete mitochondrial genomes. *Science* **308**, 1034–1036 (2005).

37. L. Pagani, T. Kivisild, A. Tarekegn, R. Ekong, C. Plaster, I. G. Romero, Q. Ayub, S. Q. Mehdi, M. G. Thomas, D. Luiselli, E. Bekele, N. Bradman, D. J. Balding, C. Tyler-Smith, Ethiopian genetic diversity reveals linguistic stratification and complex influences on the Ethiopian gene pool. *Am. J. Hum. Genet.* **91**, 83–96 (2012).

38. C. J. Bae, K. Douka, M. D. Petraglia, On the origin of modern humans: Asian perspectives. *Science* **358**, eaai9067 (2017).

39. L. N. M. Iasi, M. Chintalapati, L. Skov, A. B. Mesa, M. Hajdinjak, B. M. Peter, P. Moorjani, Neanderthal ancestry through time: Insights from genomes of ancient and present-day humans. *Science* **386**, eadq3010 (2024).

40. A. P. Sumer, H. Rougier, V. Villalba-Mouco, Y. Huang, L. N. M. Iasi, E. Essel, A. Bossoms Mesa, A. Furtwaengler, S. Peyregne, C. de Filippo, A. B. Rohrlach, F. Pierini, F. Mafessoni, H. Fewlass, E. I. Zavala, D. Mylopotamitaki, R. A. Bianco, A. Schmidt, J. Zorn, B. Nickel, A. Patova, C. Posth, G. M. Smith, K. Ruebens, V. Sinet-Mathiot, A. Stoessel, H. Dietl, J. Orschiedt, J. Kelso, H. Zeberg, K. I. Bos, F. Welker, M. Weiss, S. P. McPherron, T. Schuler, J. J. Hublin, P. Veleminsky, J. Bruzek, B. M. Peter, M. Meyer, H. Meller, H. Ringbauer, M. Hajdinjak, K. Prufer, J. Krause, Earliest modern human genomes constrain timing of Neanderthal admixture. *Nature* **638**, 711–717 (2025).

41. S. Carlhoff, A. Duli, K. Nagele, M. Nur, L. Skov, I. Sumantri, A. A. Oktaviana, B. Hakim, B. Burhan, F. A. Syahdar, D. P. McGahan, D. Bulbeck, Y. L. Perston, K. Newman, A. M. Saiful, M. Ririmasse, S. Chia, Hasanuddin, D. A. T. Pulubuhu, Suryatman, Supriadi, C. Jeong, B. M. Peter, K. Prufer, A. Powell, J. Krause, C. Posth, A. Brumm, Genome of a middle Holocene hunter-gatherer from Wallacea. *Nature* **596**, 543–547 (2021).

42. S. Oliveira, K. Nägele, S. Carlhoff, I. Pugach, T. Koesbardiati, A. Hübner, M. Meyer, A. A. Oktaviana, M. Takenaka, C. Katagiri, D. B. Murti, R. S. Putri, Mahirta, F. Petchey, T. Higham, C. F. W. Higham, S. O'Connor, S. Hawkins, R. Kinaston, P. Bellwood, R. Ono, A. Powell, J. Krause, C. Posth, M. Stoneking, Ancient genomes from the last three millennia support multiple human dispersals into Wallacea. *Nat. Ecol. Evol.* **6**, 1024–1034 (2022).

43. J. L. Wright, S. Wasef, T. H. Heupink, M. C. Westaway, S. Rasmussen, C. Pardoe, G. G. Fourmile, M. Young, T. Johnson, J. Slade, R. Kennedy, P. Winch, M. Pappin, T. Wales, W. B. Bates, S. Hamilton, N. Whyman, S. V. Pellekaan, P. J. McAllister, P. S. C. Taçon, D. Curnoe, R. Q. Li, C. Millar, S. Subramanian, E. Willerslev, A.-S. Malaspinas, M. Sikora, D. M. Lambert, Ancient nuclear genomes enable repatriation of Indigenous human remains. *Sci. Adv.* **4**, eaau5064 (2018).

44. Q. Fu, A. Mittnik, P. L. F. Johnson, K. Bos, M. Lari, R. Bollongino, C. Sun, L. Giemsch, R. Schmitz, J. Burger, A. M. Ronchitelli, F. Martini, R. G. Cremonesi, J. Svoboda, P. Bauer, D. Caramelli, S. Castellano, D. Reich, S. Pääbo, J. Krause, A revised timescale for human evolution based on ancient mitochondrial genomes. *Curr. Biol.* **23**, 553–559 (2013).

45. A. Scally, R. Durbin, Revising the human mutation rate: Implications for understanding human evolution. *Nat. Rev. Genet.* **13**, 745–753 (2012).

46. P. Soares, J. A. Trejaut, J. H. Loo, C. Hill, M. Mormina, C. L. Lee, Y. M. Chen, G. Hudjashov, P. Forster, V. MacAulay, D. Bulbeck, S. Oppenheimer, M. Lin, M. B. Richards, Climate change and postglacial human dispersals in Southeast Asia. *Mol. Biol. Evol.* **25**, 1209–1218 (2008).

47. C. Hill, P. Soares, M. Mormina, V. Macaulay, W. Meehan, J. Blackburn, D. Clarke, J. M. Raja, P. Ismail, D. Bulbeck, S. Oppenheimer, M. Richards, Phylogeography and ethnogenesis of aboriginal Southeast Asians. *Mol. Biol. Evol.* **23**, 2480–2491 (2006).

48. M. van Oven, M. Kayser, Updated comprehensive phylogenetic tree of global human mitochondrial DNA variation. *Hum. Mutat.* **30**, E386–E394 (2009).

49. S. Kumar, R. R. Ravuri, P. Koneru, B. P. Urade, B. N. Sarkar, A. Chandrasekar, V. R. Rao, Reconstructing Indian-Australian phylogenetic link. *BMC Evol. Biol.* **9**, 173 (2009).

50. P. A. Soares, J. A. Trejaut, T. Rito, B. Cavadas, C. Hill, K. K. Eng, M. Mormina, A. Brandao, R. M. Fraser, T. Y. Wang, J. H. Loo, C. Snell, T. M. Ko, A. Amorim, M. Pala, V. Macaulay, D. Bulbeck, J. F. Wilson, L. Gusmao, L. Pereira, S. Oppenheimer, M. Lin, M. B. Richards,

Resolving the ancestry of Austronesian-speaking populations. *Hum. Genet.* **135**, 309–326 (2016).

51. C. Reepmeyer, S. O'Connor, Mahirta, T. Maloney, S. Kealy, Late Pleistocene/early Holocene maritime interaction in Southeastern Indonesia - Timor Leste. *J. Archaeol. Sci.* **76**, 21–30 (2016).

52. S. O'Connor, S. Kealy, C. Reepmeyer, S. C. S. Carro, C. Shipton, Terminal Pleistocene emergence of maritime interaction networks across Wallacea. *World Archaeol.* **54**, 244–263 (2022).

53. D. Bulbeck, F. Arifin Aziz, S. O'Connor, A. Calo, J. N. Fenner, B. Marwick, J. Feathers, R. Wood, D. Prastiningtyas, Mortuary caves and the Dammar Trade in the Towuti–Routa region, Sulawesi, in an Island Southeast Asian context. *Asian Persp.* **55**, 148–183 (2016).

54. A. M. S. Ko, C. Y. Chen, Q. Fu, F. Delfin, M. Li, H. L. Chiu, M. Stoneking, Y. C. Ko, Early Austronesians: Into and out of Taiwan. *Am. J. Hum. Genet.* **94**, 426–436 (2014).

55. D. Pierron, M. Heiske, H. Razafindrazaka, V. Pereda-loth, J. Sanchez, O. Alva, A. Arachiche, A. Boland, R. Olaso, J. F. Deleuze, F. X. Ricaut, J. A. Rakotoarisoa, C. Radimilahy, M. Stoneking, T. Letellier, Strong selection during the last millennium for African ancestry in the admixed population of Madagascar. *Nat. Commun.* **9**, 932 (2018).

56. M. Haber, A. L. Jones, B. A. Connell, Asan, E. Arciero, H. M. Yang, M. G. Thomas, Y. L. Xue, C. Tyler-Smith, A rare deep-rooting D0 African Y-chromosomal haplogroup and its implications for the expansion of modern humans out of Africa. *Genetics* **212**, 1421–1428 (2019).

57. P. Soares, F. Alshamali, J. B. Pereira, V. Fernandes, N. M. Silva, C. Afonso, M. D. Costa, E. Musilová, V. Macaulay, M. B. Richards, V. Cerny, L. Pereira, The expansion of mtDNA haplogroup L3 within and out of Africa. *Mol. Biol. Evol.* **29**, 915–927 (2012).

58. Q. Fu, H. Li, P. Moorjani, F. Jay, S. M. Slepchenko, A. A. Bondarev, P. L. F. Johnson, A. Aximu-Petri, K. Prüfer, C. de Filippo, M. Meyer, N. Zwyns, D. C. Salazar-Garcia, Y. V.

Kuzmin, S. G. Keates, P. A. Kosintsev, D. I. Razhev, M. P. Richards, N. V. Peristov, M. Lachmann, K. Douka, T. F. Higham, M. Slatkin, J.-J. Hublin, D. Reich, J. Kelso, T. B. Viola, S. Pääbo, Genome sequence of a 45,000-year-old modern human from western Siberia. *Nature* **514**, 445–449 (2014).

59. M. Kayser, S. Brauer, G. Weiss, P. A. Underhill, L. Roewer, W. Schiefenhövel, M. Stoneking, Melanesian origin of Polynesian Y chromosomes. *Curr. Biol.* **10**, 1237–1246 (2000).

60. M. Kayser, S. Brauer, G. Weiss, W. Schiefenhövel, P. Underhill, P. D. Shen, P. Oefner, M. Tommaseo-Ponzetta, M. Stoneking, Reduced Y-chromosome, but not mitochondrial DNA, diversity in human populations from West New Guinea. *Am. J. Hum. Genet.* **72**, 281–302 (2003).

61. P. Soares, T. Rito, J. Trejaut, M. Mormina, C. Hill, E. Tinkler-Hundal, M. Braid, D. J. Clarke, J.-H. Loo, N. Thomson, T. Denham, M. Donohue, V. Macaulay, M. Lin, S. Oppenheimer, M. B. Richards, Ancient voyaging and Polynesian origins. *Am. J. Hum. Genet.* **88**, 239–247 (2011).

62. B. Griffiths, L. Russell, What we were told: Responses to 65,000 years of Aboriginal history. *Aborig. Hist.* **42**, 31–53 (2018).

63. C. Posth, G. Renaud, A. Mittnik, D. G. Drucker, H. Rougier, C. Cupillard, F. Valentin, C. Thevenet, A. Furtwangler, C. Wissing, M. Francken, M. Malina, M. Bolus, M. Lari, E. Gigli, G. Capecchi, I. Crevecoeur, C. Beauval, D. Flas, M. Germonpre, J. van der Plicht, R. Cottiaux, B. Gely, A. Ronchitelli, K. Wehrberger, D. Grigorescu, J. Svoboda, P. Semal, D. Caramelli, H. Bocherens, K. Harvati, N. J. Conard, W. Haak, A. Powell, J. Krause, Pleistocene mitochondrial genomes suggest a single major dispersal of non-Africans and a Late Glacial population turnover in Europe. *Curr. Biol.* **26**, 827–833 (2016).

64. P. Deckker, L. J. Arnold, S. van der Kaars, G. Bayon, J.-B. W. Stuut, K. Perner, R. Lopes dos Santos, R. Uemura, M. Demuro, Marine isotope stage 4 in Australasia: A full glacial culminating 65,000 years ago—Global connections and implications for human dispersal. *Quat. Sci. Rev.* **204**, 187e207 (2019).

65. M. I. Bird, R. J. Beaman, S. A. Condie, A. Cooper, S. Ulm, P. Veth, Palaeogeography and voyage modeling indicates early human colonization of Australia was likely from Timor-Roti. *Quat. Sci. Rev.* **191**, 431–439 (2018).

66. J. B. Birdsell, in *Sunda and Sahul: Prehistoric Studies in Southeast Asia, Melanesia and Australia*, J. Allen, J. Golson, R. Jones, Eds. (Academic Press, 1977), pp. 113–167.

67. C. J. A. Bradshaw, K. Norman, S. Ulm, A. N. Williams, C. Clarkson, J. Chadœuf, S. C. Lin, Z. Jacobs, R. G. Roberts, M. I. Bird, L. S. Weyrich, S. G. Haberle, S. O'Connor, B. Llamas, T. J. Cohen, T. Friedrich, P. Veth, M. Leavesley, F. Saltré, Stochastic models support rapid peopling of Late Pleistocene Sahul. *Nat. Commun.* **12**, 2440 (2021).

68. S. Kealy, J. Louys, S. O'Connor, Least-cost pathway models indicate northern human dispersal from Sunda to Sahul. *J. Hum. Evol.* **125**, 59–70 (2018).

69. G. Irwin, *The Prehistoric Exploration and Colonisation of the Pacific* (Cambridge Univ. Press, 2010).

70. K. Norman, J. Inglis, C. Clarkson, J. T. Faith, J. Shulmeister, D. Harris, An early colonisation pathway into northwest Australia 70-60,000 years ago. *Quat. Sci. Rev.* **180**, 229–239 (2018).

71. E. Tamm, T. Kivisild, M. Reidla, M. Metspalu, D. G. Smith, C. J. Mulligan, C. M. Bravi, O. Rickards, C. Martinez-Labarga, E. K. Khusnutdinova, S. A. Fedorova, M. V. Gulubenko, V. A. Stepanov, M. A. Gubina, S. I. Zhadanov, L. P. Ossipova, L. Damba, M. I. Voevoda, J. E. Dipierra, R. Villems, R. S. Malhi, Beringian standstill and spread of Native American founders. *PLOS ONE* **2**, e829 (2007).

72. S. M. Gomes, M. Bodner, L. Souto, B. Zimmermann, G. Huber, C. Strobl, A. W. Röck, A. Achilli, A. Olivieri, A. Torroni, F. Côrte-Real, W. Parson, Human settlement history between Sunda and Sahul: A focus on East Timor (Timor-Leste) and the Pleistocenic mtDNA diversity. *BMC Genomics* **16**, 70 (2015).

73. J. E. Terrell, The 'sleeping giant' hypothesis and New Guinea's place in the prehistory of Greater Near Oceania. *World Archaeol.* **36**, 601–609 (2004).

74. P. McAllister, N. Nagle, R. J. Mitchell, The Australian Barrineans and their relationship to Southeast Asian negritos: An investigation using mitochondrial genomics. *Hum. Biol.* **85**, 485–494 (2013).

75. F. Delfin, A. M.-S. Ko, M. Li, E. D. Gunnarsdóttir, K. A. Tabbada, J. M. Salvador, G. C. Calacal, M. S. Sagum, F. A. Datar, S. G. Padilla, M. C. A. De Ungria, M. Stoneking, Complete mtDNA genomes of Filipino ethnolinguistic groups: A melting pot of recent and ancient lineages in the Asia-Pacific region. *Eur. J. Hum. Genet.* **22**, 228–237 (2013).

76. M. Arenas, A. Gorostiza, J. M. Baquero, E. Campoy, C. Branco, H. Rangel-Villalobos, A. González-Martín, The early peopling of the Philippines based on mtDNA. *Sci. Rep.* **10**, 4901 (2020).

77. M. Larena, F. Sanchez-Quinto, P. Sjödin, J. McKenna, C. Ebeo, R. Reyes, O. Casel, J.-Y. Huang, K. P. Hagada, D. Guilay, J. Reyes, F. P. Allian, V. Mori, L. S. Azarcon, A. Manera, C. Terando, L. Jamero Jr., G. Sireg, R. Manginsay-Tremedal, M. S. Labos, R. D. Vilar, A. Latiph, R. L. Saway, E. Marte, P. Magbanua, A. Morales, I. Java, R. Reveche, B. Barrios, E. Burton, J. C. Salon, M. J. T. Kels, A. Albano, R. B. Cruz-Angeles, E. Molanida, L. Granehäll, M. Vicente, H. Edlund, J.-H. Loo, J. Trejaut, S. Y. W. Ho, L. Reid, H. Malmström, C. Schlebusch, K. Lambeck, P. Endicott, M. Jakobsson, Multiple migrations to the Philippines during the last 50,000 years. *Proc. Natl. Acad. Sci. U.S.A.* **118**, e2026132118 (2021).

78. G. S. Jacobs, G. Hudjashov, S. Lauri, K. Pradiptajati, C. C. Darusallam, D. J. Lawson, M. Mayukh, L. Pagani, F.-X. Ricaut, M. Stoneking, M. Metspalu, H. Sudoyo, J. S. Lansing, M. P. Cox, Multiple deeply divergent Denisovan ancestries in Papuans. *Cell* **177**, 1010–1021.e32 (2019).

79. K. Ditchfield, S. Ulm, T. Manne, H. Farr, D. O'Grady, P. Veth, Framing Australian Pleistocene coastal occupation and archaeology. *Quat. Sci. Rev.* **293**, 107706 (2022).

80. B. P. McEvoy, J. M. Lind, E. T. Wang, R. K. Moyzis, P. M. Visscher, S. M. van Holst Pellekaan, A. N. Wilton, Whole-genome genetic diversity in a sample of Australians with deep Aboriginal ancestry. *Am. J. Hum. Genet.* **87**, 297–305 (2010).

81. G. Hudjashov, T. M. Karafet, D. J. Lawson, S. Downey, O. Savina, H. Sudoyo, J. S. Lansing, M. F. Hammer, M. P. Cox, Complex patterns of admixture across the Indonesian Archipelago. *Mol. Biol. Evol.* **34**, 2439–2452 (2017).

82. S. Brandini, P. Bergamaschi, M. F. Cerna, F. Gandini, F. Bastaroli, E. Bertolini, C. Cereda, L. Ferretti, A. Gómez-Carballa, V. Battaglia, A. Salas, O. Semino, A. Achilli, A. Olivieri, A. Torroni, The Paleo-Indian entry into South America according to mitogenomes. *Mol. Biol. Evol.* **35**, 299–311 (2018).

83. K. Dulias, S. Birch, J. F. Wilson, P. Justeau, F. Gandini, A. Flaquer, P. Soares, M. Pala, M. B. Richards, C. J. Edwards, Maternal relationships within an Iron Age burial at the High Pasture Cave, Isle of Skye, Scotland. *J. Archaeol. Sci.* **110**, 104978 (2019).

84. H. Weissensteiner, D. Pacher, A. Kloss-Brandstätter, L. Forer, G. Specht, H.-J. Bandelt, F. Kronenberg, A. Salas, S. Schönherr, HaploGrep 2: Mitochondrial haplogroup classification in the era of high-throughput sequencing. *Nucleic Acids Res.* **44**, W58–W63 (2016).

85. R. M. Andrews, I. Kubacka, P. F. Chinnery, R. N. Lightowlers, D. M. Turnbull, N. Howell, Reanalysis and revision of the Cambridge reference sequence for human mitochondrial DNA. *Nat. Genet.* **23**, 147 (1999).

86. T. M. Keane, C. J. Creevey, M. M. Pentony, T. J. Naughton, J. O. Mclnerney, Assessment of methods for amino acid matrix selection and their use on empirical data shows that ad hoc assumptions for choice of matrix are not justified. *BMC Evol. Biol.* **6**, 29 (2006).

87. P. Forster, R. Harding, A. Torroni, H.-J. Bandelt, Origin and evolution of Native American mtDNA variation: A reappraisal. *Am. J. Hum. Genet.* **59**, 935–945 (1996).

88. J. Saillard, P. Forster, N. Lynnerup, H.-J. Bandelt, S. S. Nørby, mtDNA variation among Greenland Eskimos: The edge of the Beringian expansion. *Am. J. Hum. Genet.* **67**, 718–726 (2000).

89. Z. Yang, PAML: A program package for phylogenetic analysis by maximum likelihood. *CABIOS* **13**, 555–556 (1997).

90. A. J. Drummond, A. Rambaut, BEAST: Bayesian evolutionary analysis by sampling trees. *BMC Evol. Biol.* **7**, 214 (2007).

91. P. Danecek, A. Auton, G. Abecasis, C. A. Albers, E. Banks, M. A. DePristo, R. E. Handsaker, G. Lunter, G. T. Marth, S. T. Sherry, G. McVean, R. Durbin, 1000 Genomes Project Analysis Group, Genomes Project Analysis, the variant call format and VCFtools. *Bioinformatics* **27**, 2156–2158 (2011).

92. D. H. Alexander, J. Novembre, K. Lange, Fast model-based estimation of ancestry in unrelated individuals. *Genome Res.* **19**, 1655–1664 (2009).

93. A. L. Price, N. J. Patterson, R. M. Plenge, M. E. Weinblatt, N. A. Shadick, D. Reich, Principal components analysis corrects for stratification in genome-wide association studies. *Nat. Genet.* **38**, 904–909 (2006).

94. A. Cooper, A. Rambaut, V. Macaulay, E. Willerslev, A. J. Hansen, C. Stringer, Human origins and ancient human DNA. *Science* **292**, 1655–1656 (2001).

95. T. H. Heupink, S. Subramanian, J. L. Wright, P. Endicott, M. C. Westaway, L. Huynen, W. Parson, C. D. Millar, E. Willerslev, D. M. Lambert, Ancient mtDNA sequences from the First Australians revisited. *Proc. Natl. Acad. Sci. U.S.A.* **113**, 6892–6897 (2016).

96. D. Y. Yang, B. Eng, J. S. Waye, J. C. Dudar, S. R. Saunders, Improved DNA extraction from ancient bones using silica-based spin columns. *Am. J. Phys. Anthropol.* **105**, 539–543 (1998).

97. D. E. MacHugh, C. J. Edwards, J. F. Bailey, D. R. Bancroft, D. G. Bradley, The extraction and analysis of ancient DNA from bone and teeth: A survey of current methodologies. *Ancient Biomolecules* **3**, 81–102 (2000).

98. M. Meyer, M. Kircher, Illumina sequencing library preparation for highly multiplexed target capture and sequencing. *Cold Spring Harb. Protoc.* **2010**, pdb.prot5448 (2010).

99. I. Mathieson, I. Lazaridis, N. Rohland, S. Mallick, N. Patterson, S. A. Roodenberg, E. Harney, K. Stewardson, D. Fernandes, M. Novak, K. Sirak, C. Gamba, E. R. Jones, B. Llamas, S. Dryomov, J. Pickrell, J. L. Arsuaga, J. M. B. de Castro, E. Carbonell, F. Gerritsen,

A. Khokhlov, P. Kuznetsov, M. Lozano, H. Meller, O. Mochalov, V. Moiseyev, M. A. R. Guerra, J. Roodenberg, J. M. Vergès, J. Krause, A. Cooper, K. W. Alt, D. Brown, D. Anthony, C. Lalueza-Fox, W. Haak, R. Pinhasi, D. Reich, Genome-wide patterns of selection in 230 ancient Eurasians. *Nature* **528**, 499–503 (2015).

100. M. Lipson, P. Skoglund, M. Spriggs, F. Valentin, S. Bedford, R. Shing, H. Buckley, I. Phillip, G. K. Ward, S. Mallick, N. Rohland, N. Broomandkhoshbacht, O. Cheronet, M. Ferry, T. K. Harper, M. Michel, J. Oppenheimer, K. Sirak, K. Stewardson, K. Auckland, A. V. S. Hill, K. Maitland, S. J. Oppenheimer, T. Parks, K. Robson, T. N. Williams, D. J. Kennett, A. J. Mentzer, R. Pinhasi, D. Reich, Population turnover in Remote Oceania shortly after initial settlement. *Curr. Biol.* **28**, 11157–11165.e7 (2018).

101. M. Pala, G. Chaubey, P. Soares, M. B. Richards, *Encyclopedia of Life Sciences (ELS)* (John Wiley and Sons, 2014).

102. P. Soares, T. Rito, L. Pereira, M. B. Richards, in *Africa from MIS 6 to 2*, S. C. Jones, B. A. Stewart, Eds. (Springer, 2016).

103. T. Rieth, E. E. Cochrane, in *The Oxford Handbook of Prehistoric Oceania*, E. E. Cochrane, T. L. Hunt, Eds. (Oxford Univ. Press, 2018), chap. 6, pp. 133–161.

104. S. Bedford, M. Spriggs, in *The Oxford Handbook of Prehistoric Oceania*, E. E. Cochrane, T. L. Hunt, Eds. (Oxford Univ. Press, 2018), chap. 7, pp. 162–184.

105. S. Bedford, M. Spriggs, D. V. Burley, C. Sand, P. Sheppard, G. R. Summerhayes, in *Debating Lapita*, S. Bedford, M. Spriggs, Eds. (Australian National Univ. Press, 2019), chap. 1, pp. 5–36.

106. M. Almeida, F. Gandini, T. Rito, M. George Foody, A. Brandão, M. Oliveira, A. Olivieri, A. Fichera, G. Oteo-Garcia, Z. Zainuddin, K. K. Eng, W. Pomat, J. Bryk, L. Pereira, H. Farr, M. Pala, S. J. Oppenheimer, M. B. Richards, P. Soares, Leveraging known Pacific colonisation times to test models for the ancestry of Southeast Asians. *Sci. Rep.* **15**, 37044 (2025).

107. V. Macaulay, M. B. Richards, *Encyclopedia of Life Sciences (ELS)* (John Wiley & Sons Ltd., 2013).

108. T. Kivisild, P. Shen, D. P. Wall, B. Do, R. Sung, K. Davis, G. Passarino, P. A. Underhill, C. Scharfe, A. Torroni, R. Scozzari, D. Modiano, A. Coppa, P. de Knijff, M. Feldman, L. L. Cavalli-Sforza, P. Oefner, The role of selection in the evolution of human mitochondrial genomes. *Genetics* **172**, 373–387 (2006).

109. P. Soares, D. Abrantes, T. Rito, N. Thomson, P. Radivojac, B. Li, V. Macaulay, D. C. Samuels, L. Pereira, Evaluating purifying selection in the mitochondrial DNA of various mammalian species. *PLOS ONE* **8**, e58993 (2013).

110. A. T. Duggan, B. Evans, F. R. Friedlaender, J. S. Friedlaender, G. Koki, D. A. Merriwether, M. Kayser, M. Stoneking, Maternal history of Oceania from complete mtDNA genomes: Contrasting ancient diversity with recent homogenization due to the Austronesian expansion. *Am. J. Hum. Genet.* **94**, 721–733 (2014).

111. D. A. Merriwether, J. A. Hodgson, F. R. Friedlaender, R. Allaby, S. Cerchio, G. Koki, J. S. Friedlaender, Ancient mitochondrial M haplogroups identified in the Southwest Pacific. *Proc. Natl. Acad. Sci. U.S.A.* **102**, 13034–13039 (2005).

112. P. Mitchell, Settling Madagascar: When did people first colonize the world's largest island? *J. Isl. Coast. Archaeol.* **15**, 576–595 (2020).

113. S. O'Connor, D. Bulbeck, P. J. Piper, F. Aziz, B. Marwick, F. Campos, J. Fenner, K. Aplin, Suryatman, Fakhri, T. Maloney, B. Hakim, R. Wood, in *The Archaeology of Sulawesi: Current Research on the Pleistocene to the Historic Period*, S. O'Connor, D. Bulbeck, J. Meyer, Eds. (ANU Press, 2018), chap. 9, vol. 48, pp. 117–151.

114. C. Hill, P. Soares, M. Mormina, V. Macaulay, D. Clarke, P. B. Blumbach, M. Vizuete-Forster, P. Forster, D. Bulbeck, S. Oppenheimer, M. Richards, A mitochondrial stratigraphy for Island Southeast Asia. *Am. J. Hum. Genet.* **80**, 29–43 (2007).

115. C.-H. Tsang, K.-T. Li, T.-F. Hsu, Y.-C. Tsai, P.-H. Fang, Y.-l. C. Hsing, Broomcorn and foxtail millet were cultivated in Taiwan about 5000 years ago. *Bot. Stud.* **58**, 3 (2017).

116. I. Pugach, A. Hubner, H.-C. Hung, M. Meyer, M. T. Carson, M. Stoneking, Ancient DNA from Guam and the peopling of the Pacific. *Proc. Natl. Acad. Sci. U.S.A.* **118**, e2022112118 (2021).

117. S. van Holst Pellekaan, Genetic evidence for the colonization of Australia. *Quat. Int.* **285**, 44–56 (2013).

118. D. Adamov, V. Guryanov, S. Karzhavin, V. Tagankin, V. Urasin, Defining a new rate constant for Y-chromosome SNPs based on full sequencing data. *Russ. J. Genet. Geneal. (Русская версия)* **7**, 68–89 (2015).

119. Q. Fu, M. Hajdinjak, O. T. Moldovan, S. Constantin, S. Mallick, P. Skoglund, N. Patterson, N. Rohland, I. Lazaridis, B. Nickel, B. Viola, K. Prufer, M. Meyer, J. Kelso, D. Reich, S. Paabo, An early modern human from Romania with a recent Neanderthal ancestor. *Nature* **524**, 216–219 (2015).

120. Q. M. Fu, C. Posth, M. Hajdinjak, M. Petr, S. Mallick, D. Fernandes, A. Furtwangler, W. Haak, M. Meyer, A. Mittnik, B. Nickel, A. Peltzer, N. Rohland, V. Slon, S. Talamo, I. Lazaridis, M. Lipson, I. Mathieson, S. Schiffels, P. Skoglund, A. P. Derevianko, N. Drozdov, V. Slavinsky, A. Tsybankov, R. G. Cremonesi, F. Mallegni, B. Gely, E. Vacca, M. R. G. Morales, L. G. Straus, C. Neugebauer-Maresch, M. Teschler-Nicola, S. Constantin, O. T. Moldovan, S. Benazzi, M. Peresani, D. Coppola, M. Lari, S. Ricci, A. Ronchitelli, F. Valentin, C. Thevenet, K. Wehrberger, D. Grigorescu, H. Rougier, I. Crevecoeur, D. Flas, P. Semal, M. A. Mannino, C. Cupillard, H. Bocherens, N. J. Conard, K. Harvati, V. Moiseyev, D. G. Drucker, J. Svoboda, M. P. Richards, D. Caramelli, R. Pinhasi, J. Kelso, N. Patterson, J. Krause, S. Paabo, D. Reich, The genetic history of Ice Age Europe. *Nature* **534**, 200–205 (2016).

121. S. R. Browning, B. L. Browning, Y. Zhou, S. Tucci, J. M. Akey, Analysis of human sequence data reveals two pulses of archaic Denisovan admixture. *Cell* **173**, 53–61.e9 (2018).

122. A. Helgason, A. W. Einarsson, V. B. Guðmundsdóttir, Á. Sigurðsson, E. D. Gunnarsdóttir, A. Jagadeesan, S. S. Ebenesersdóttir, A. Kong, K. Stefánsson, The Y-chromosome point mutation rate in humans. *Nat. Genet.* **47**, 453–457 (2015).

123. S. Sankararaman, S. Mallick, N. Patterson, D. Reich, The combined landscape of Denisovan and Neanderthal ancestry in present-day humans. *Curr. Biol.* **26**, 1241–1247 (2016).

124. P. Moorjani, S. Sankararaman, Q. Fu, M. Przeworski, N. Patterson, D. Reich, A genetic method for dating ancient genomes provides a direct estimate of human generation interval in the last 45,000 years. *Proc. Natl. Acad. Sci. U.S.A.* **113**, 5652–5657 (2016).

125. L. N. M. Iasi, H. Ringbauer, B. M. Peter, An extended admixture pulse model reveals the limitations to human-Neandertal introgression dating. *Mol. Biol. Evol.* **38**, 5156–5174 (2021).

126. N. Nagle, K. N. Ballantyne, M. van Oven, C. Tyler-Smith, Y. Xue, D. Taylor, S. Wilcox, L. Wilcox, R. Turkalov, R. A. H. van Oorschot, P. McAllister, L. Williams, M. Kayser, R. J. Mitchell, Genographic Consortium, Antiquity and diversity of aboriginal Australian Y-chromosomes. *Am. J. Phys. Anthropol.* **159**, 367–381 (2016).

127. D. D. Anderson, Cave archaeology in Southeast Asia. *Geoarchaeology* **12**, 607–638 (1997).

128. G. Barker, H. Barton, M. Bird, P. Daly, I. Datan, A. Dykes, L. Farr, D. Gilbertson, B. Harrisson, C. Hunt, T. Higham, L. Kealhofer, J. Krigbaum, H. Lewis, S. M. Laren, V. Paz, A. Pike, P. Piper, B. Pyatt, R. Rabett, T. Reynolds, J. Rose, G. Rushworth, M. Stephens, C. Stringer, J. Thompson, C. Turney, The 'human revolution' in lowland tropical Southeast Asia: The antiquity and behavior of anatomically modern humans at Niah Cave (Sarawak, Borneo). *J. Hum. Evol.* **52**, 243–261 (2007).

129. M. Aubert, A. Brumm, P. S. C. Taçon, The timing and nature of human colonization in Southeast Asia in the Late Pleistocene: A rock art perspective. *Curr. Anthropol.* **58**, S553–S566 (2017).

130. M. Aubert, P. Setiawan, A. A. Oktaviana, A. Brumm, P. H. Sulistyarto, E. W. Saptomo, B. Istiawan, T. A. Ma'rifat, V. N. Wahyuono, F. T. Atmoko, J.-X. Zhao, J. Huntley, P. S. C.

Taçon, D. L. Howard, H. E. A. Brand, Palaeolithic cave art in Borneo. *Nature* **564**, 254–257 (2018).

131. A. Brumm, A. A. Oktaviana, B. Burhan, B. Hakim, R. Lebe, J.-X. Zhao, P. H. Sulistyarto, M. Ririmasse, S. Adhityatama, I. Sumantri, M. Aubert, Oldest cave art found in Sulawesi. *Sci. Adv.* **7**, eabd4648 (2021).

132. A. A. Oktaviana, R. Joannes-Boyau, B. Hakim, B. Burhan, R. Sardi, S. Adhityatama, Hamrullah, I. Sumantri, M. Tang, R. Lebe, I. Ilyas, A. Abbas, A. Jusdi, D. E. Mahardian, S. Noerwidi, M. N. R. Ririmasse, I. Mahmud, A. Duli, L. M. Aksa, D. McGahan, P. Setiawan, A. Brumm, M. Aubert, Narrative cave art in Indonesia by 51,200 years ago. *Nature* **631**, 814–818 (2024).

133. F. Détroit, E. Dizon, C. Falguères, S. Hameau, W. Ronquillo, F. Sémah, Upper Pleistocene *Homo sapiens* from the Tabon cave (Palawan, The Philippines): Description and dating of new discoveries. *C. R. Palevol* **3**, 705–712 (2004).

134. H. A. F. Kaharudin, S. O'Connor, S. Kealy, M. N. Ririmasse, Islands on the edge: 42,000-year-old occupation of the Tanimbar islands and its implications for the Sunda-Sahul early human migration discourse. *Quat. Sci. Rev.* **338**, 108834 (2024).

135. S. Hawkins, S. O'Connor, T. R. Maloney, M. Litster, S. Kealy, J. N. Fenner, K. Aplin, C. Boulanger, S. Brockwell, R. Willan, E. Piotto, J. Louys, Oldest human occupation of Wallacea at Laili Cave, Timor-Leste, shows broad-spectrum foraging responses to late Pleistocene environments. *Quat. Sci. Rev.* **171**, 58–72 (2017).

136. C. Shipton, S. O'Connor, N. Jankowski, J. O'Connor-Veth, T. Maloney, S. Kealy, C. Boulanger, A new 44,000-year sequence from Asitau Kuru (Jerimalai), Timor-Leste, indicates long-term continuity in human behaviour. *Archaeol. Anthropol. Sci.* **11**, 5717–5741 (2019).

137. S. O'Connor, A. Barham, M. Spriggs, P. Veth, K. Aplin, E. St Pierre, Cave archaeology and sampling issues in the tropics: A case study from Lene Hara Cave, a 42,000 year old occupation site in East Timor, Island Southeast Asia. *Aust. Archaeol.* **71**, 29–40 (2010).

138. J. Chappell, A. Omura, T. Esat, M. McCulloch, J. Pandolfi, Y. Ota, B. Pillans, Reconciliation of late Quaternary sea levels derived from coral terraces at Huon Peninsula with deep sea oxygen isotope records. *Earth Planet. Sci. Lett.* **141**, 227–236 (1996).

139. L. Groube, J. Chappell, J. Muke, D. Price, A 40,000 year-old human occupation site at the Huon Peninsula, Papua New Guinea. *Nature* **324**, 453–455 (1986).

140. R. G. Roberts, Luminescence dating in archaeology: From origins to optical. *Radiat. Meas.* **27**, 819–892 (1997).

141. G. R. Summerhayes, M. Leavesley, A. Fairbairn, H. Mandui, J. Field, A. Ford, R. Fullagar, Human adaptation and plant use in Highland New Guinea 49,000 to 44,000 years ago. *Science* **330**, 78–81 (2010).

142. R. Roberts, R. Jones, M. A. Smith, Optical dating at Deaf Adder Gorge, Northern Territory, indicates human occupation between 53,000 and 60,000 years ago. *Aust. Archaeol.* **37**, 58–59 (1993).

143. R. G. Roberts, R. Jones, N. A. Spooner, M. J. Head, A. S. Murray, M. A. Smith, The human colonisation of Australia: Optical dates of 53,000 and 60,000 years bracket human arrival at Deaf Adder Gorge, Northern Territory. *Quat. Sci. Rev.* **13**, 575–583 (1994).

144. B. David, J.-J. Delannoy, J. Mialanes, C. Clarkson, F. Petchey, J.-M. Geneste, T. Manne, M. I. Bird, B. Barker, T. Richards, E. Chalmin, G. Castets, 45,610–52,160 years of site and landscape occupation at Nawarla Gabarnmang, Arnhem Land plateau (northern Australia). *Quat. Sci. Rev.* **215**, 64–85 (2019).

145. P. Veth, K. Ditchfield, M. Bateman, S. Ouzman, M. Benoit, A. P. Motta, D. Lewis, S. Harper, Balanggarra Aboriginal Corporation, Minjiwarra: Archaeological evidence of human occupation of Australia's northern Kimberley by 50,000 BP. *Aust. Archaeol.* **85**, 115–125 (2019).

146. T. Maloney, S. O'Connor, R. Wood, K. Aplin, J. Balme, Carpenters Gap 1: A 47,000 year old record of indigenous adaption and innovation. *Quat. Sci. Rev.* **191**, 204–228 (2018).

147. R. Wood, Z. Jacobs, D. Vannieuwenhuyse, J. Balme, S. O'Connor, R. Whitau, Towards an accurate and precise chronology for the colonization of Australia: The example of Riwi, Kimberley, Western Australia. *PLOS ONE* **11**, e0160123 (2016).

148. P. Veth, M. Smith, J. Bowler, K. Fitzsimmons, A. Williams, P. Hiscock, Excavations at Parnkupirti, Lake Gregory, Great Sandy Desert: OSL ages for occupation before the Last Glacial Maximum. *Aust. Archaeol.* **69**, 1–10 (2009).

149. P. Veth, I. Ward, T. Manne, S. Ulm, K. Ditchfield, J. Dortch, F. Hook, F. Petchey, A. Hogg, D. Questiaux, M. Demuro, L. Arnold, N. Spooner, M. Demuro, V. Levchenko, J. Skippington, C. Byrne, M. Basgall, D. Zeanah, D. Belton, P. Helmholz, P. Kendrick, Early human occupation of a maritime desert, Barrow Island, North-West Australia. *Quat. Sci. Rev.* **168**, 19–29 (2017).

150. J. McDonald, W. Reynen, F. Petchey, K. Ditchfield, C. Byrne, D. Vannieuwenhuyse, M. Leopold, P. Veth, Karnatukul (Serpent's Glen): A new chronology for the oldest site in Australia's Western Desert. *PLOS ONE* **13**, e0202511 (2018).

151. C. S. M. Turney, M. I. Bird, L. K. Fifield, R. G. Roberts, M. Smith, C. E. Dortch, R. Grun, E. Lawson, L. K. Ayliffe, G. H. Miller, J. Dortch, R. G. Cresswell, Early human occupation at Devil's Lair, southwestern Australia 50,000 years ago. *Quat. Res.* **55**, 3–13 (2001).

152. J. M. Bowler, H. Johnston, J. M. Olley, J. R. Prescott, R. G. Roberts, W. Shawcross, N. A. Spooner, New ages for human occupation and climatic change at Lake Mungo, Australia. *Nature* **421**, 837–840 (2003).

153. G. Hamm, P. Mitchell, L. J. Arnold, G. J. Prideaux, D. Questiaux, N. Spooner, V. A. Levchenko, E. C. Foley, T. H. Worthy, B. Stephenson, V. Coulthard, C. Coulthard, S. Wilton, D. Johnston, Cultural innovation and megafauna interaction in the early settlement of arid Australia. *Nature* **539**, 280–283 (2016).

154.1000G enomes Project Consortium, A. Auton, L. D. Brooks, R. M. Durbin, E. P. Garrison, H. M. Kang, J. O. Korbel, J. L. Marchini, S. McCarthy, G. A. McVean, G. R. Abecasis, A global reference for human genetic variation. *Nature* **526**, 68–74 (2015).

155. K. K. Abu-Amero, A. M. González, J. M. Larruga, T. M. Bosley, V. M. Cabrera, Eurasian and African mitochondrial DNA influences in the Saudi Arabian population. *BMC Evol. Biol.* **7**, 32 (2007).

156. D. M. Behar, M. van Oven, S. Rosset, M. Metspalu, E.-L. Loogväli, N. M. Silva, T. Kivisild, A. Torroni, R. Villems, A "Copernican" reassessment of the human mitochondrial DNA tree from its root. *Am. J. Hum. Genet.* **90**, 675–684 (2012).

157. N. Brucato, V. Fernandes, P. Kusuma, V. Černý, C. J. Mulligan, P. Soares, T. Rito, C. Besse, A. Boland, J.-F. Deleuze, M. P. Cox, H. Sudoyo, M. Stoneking, L. Pereira, F.-X. Ricaut, Evidence of Austronesian genetic lineages in East Africa and South Arabia: Complex dispersal from Madagascar and Southeast Asia. *Genome Biol. Evol.* **11**, 748–758 (2019).

158. A. Chandrasekar, S. Kumar, J. Sreenath, B. N. Sarkar, B. P. Urade, S. Mallick, S. S. Bandopadhyay, P. Barua, S. S. Barik, D. Basu, U. Kiran, P. Gangopadhyay, R. Sahani, B. V. R. Prasad, S. Gangopadhyay, G. R. Lakshmi, R. R. Ravuri, K. Padmaja, P. N. Venugopal, M. B. Sharma, V. R. Rao, Updating phylogeny of mitochondrial DNA macrohaplogroup M in India: Dispersal of modern human in South Asian corridor. *PLOS ONE* **4**, e7447 (2009).

159. J. Friedlaender, T. Schurr, F. Gentz, G. Koki, F. Friedlaender, G. Horvat, P. Babb, S. Cerchio, F. Kaestle, M. Schanfield, R. Deka, R. Yanagihara, D. A. Merriwether, Expanding Southwest Pacific mitochondrial haplogroups P and Q. *Mol. Biol. Evol.* **22**, 1506–1517 (2005).

160. M. Derenko, B. Malyarchuk, A. Bahmanimehr, G. Denisova, M. Perkova, S. Farjadian, L. Yepiskoposyan, Complete mitochondrial DNA diversity in Iranians. *PLOS ONE* **8**, e80673 (2013).

161. N. T. Duong, E. Macholdt, N. D. Ton, L. Arias, R. Schröder, N. Van Phong, V. T. B. Thuy, N. H. Ha, H. T. T. Hue, N. T. Xuan, K. T. P. Oanh, L. T. T. Hien, N. H. Hoang, B. Pakendorf, M. Stoneking, N. Van Hai, Complete human mtDNA genome sequences from Vietnam and the phylogeography of Mainland Southeast Asia. *Sci. Rep.* **8**, 11651 (2018).

162. R. Fregel, K. Seetah, E. Betancor, N. M. Suárez, D. Calaon, S. Čaval, A. Janoo, J. Pestano, Multiple ethnic origins of mitochondrial DNA lineages for the population of Mauritius. *PLOS ONE* **9**, e93294 (2014).

163. J. S. Friedlaender, F. R. Friedlaender, J. A. Hodgson, M. Stoltz, G. Koki, G. Horvat, S. Zhadanov, T. G. Schurr, D. A. Merriwether, Melanesian mtDNA complexity. *PLOS ONE* **2**, e248 (2007).

164. FamilyTreeDNA. Public Y-DNA and mtDNA results. https://familytreedna.com.

165. E. D. Gunnarsdóttir, M. Li, M. Bauchet, K. Finstermeier, M. Stoneking, High-throughput sequencing of complete human mtDNA genomes from the Philippines. *Genome Res.* **21**, 1–11 (2011).

166. A. Hartmann, M. Thieme, L. K. Nanduri, T. Stempfl, C. Moehle, T. Kivisild, P. J. Oefner, Validation of microarray-based resequencing of 93 worldwide mitochondrial genomes. *Hum. Mutat.* **30**, 115–122 (2009).

167. G. Hudjashov, P. Endicott, H. Post, N. Nagle, S. Y. W. Ho, D. J. Lawson, M. Reidla, M. Karmin, S. Rootsi, E. Metspalu, L. Saag, R. Villems, M. P. Cox, R. J. Mitchell, R. L. Garcia-Bertrand, M. Metspalu, R. J. Herrera, Investigating the origins of eastern Polynesians using genome-wide data from the Leeward Society Isles. *Sci. Rep.* **8**, 1823 (2018).

168. M. Ingman, U. Gyllensten, Mitochondrial genome variation and evolutionary history of Australian and New Guinean aborigines. *Genome Res.* **13**, 1600–1606 (2003).

169. M. Ingman, H. Kaessmann, S. Pääbo, U. Gyllensten, Mitochondrial genome variation and the origin of modern humans. *Nature* **408**, 708–713 (2000).

170. T. A. Jinam, L.-C. Hong, M. E. Phipps, M. Stoneking, M. Ameen, J. Edo, HUGO Pan-Asian SNP Consortium, N. Saitou, Evolutionary history of continental southeast Asians: “Early train” hypothesis based on genetic analysis of mitochondrial and autosomal DNA data. *Mol. Biol. Evol.* **29**, 3513–3527 (2012).

171. N. A. Khan, P. Govindaraj, N. Soumittra, S. Sharma, S. Srilekha, S. Ambika, A. Vanniarajan, A. K. Meena, M. S. Uppin, C. Sundaram, P. S. Bindu, N. Gayathri, A. B. Taly, K. Thangaraj, Leber's hereditary optic neuropathy-specific mutation m.11778G>A exists on diverse mitochondrial haplogroups in India. *Invest. Ophthalmol. Vis. Sci.* **58**, 3923–3930 (2017).

172. A. Kloss-Brandstätter, M. Summerer, D. Horst, B. Horst, G. Streiter, J. Raschenberger, F. Kronenberg, T. Sanguansermsri, J. Horst, H. Weissensteiner, An in-depth analysis of the mitochondrial phylogenetic landscape of Cambodia. *Sci. Rep.* **11**, 10816 (2021).

173. Q.-P. Kong, C. Sun, H.-W. Wang, M. Zhao, W.-Z. Wang, L. Zhong, X.-D. Hao, H. Pan, S.-Y. Wang, Y.-T. Cheng, C.-L. Zhu, S.-F. Wu, L.-N. Liu, J.-Q. Jin, Y.-G. Yao, Y.-P. Zhang, Large-scale mtDNA screening reveals a surprising matrilineal complexity in East Asia and its implications to the peopling of the region. *Mol. Biol. Evol.* **28**, 513–522 (2011).

174. W. Kutanan, J. Kampuansai, A. Brunelli, S. Ghirotto, P. Pittayaporn, S. Ruangchai, R. Schröder, E. Macholdt, M. Srikummool, D. Kangwanpong, A. Hübner, L. Arias, M. Stoneking, New insights from Thailand into the maternal genetic history of Mainland Southeast Asia. *Eur. J. Hum. Genet.* **26**, 898–911 (2018).

175. W. Kutanan, J. Kampuansai, M. Srikummool, D. Kangwanpong, S. Ghirotto, A. Brunelli, M. Stoneking, Complete mitochondrial genomes of Thai and Lao populations indicate an ancient origin of Austroasiatic groups and demic diffusion in the spread of Tai-Kadai languages. *Hum. Genet.* **136**, 85–98 (2017).

176. J. M. Larruga, P. Marrero, K. K. Abu-Amero, M. V. Golubenko, V. M. Cabrera, Carriers of mitochondrial DNA macrohaplogroup R colonized Eurasia and Australasia from a southeast Asia core area. *BMC Evol. Biol.* **17**, 115 (2017).

177. S. Lippold, H. Xu, A. Ko, M. Li, G. Renaud, A. Butthof, R. Schröder, M. Stoneking, Human paternal and maternal demographic histories: Insights from high-resolution Y chromosome and mtDNA sequences. *Investig. Genet.* **5**, 13 (2014).

178. J.-H. Loo, J. A. Trejaut, J.-C. Yen, Z.-S. Chen, C.-L. Lee, M. Lin, Genetic affinities between the Yami tribe people of Orchid Island and the Philippine Islanders of the Batanes archipelago. *BMC Genet.* **12**, 21 (2011).

179. J.-H. Loo, J. A. Trejaut, J.-C. Yen, Z.-S. Chen, W.-M. Ng, C.-Y. Huang, K.-N. Hsu, K.-H. Hung, Y. Hsiao, Y.-H. Wei, M. Lin, Mitochondrial DNA association study of type 2 diabetes with or without ischemic stroke in Taiwan. *BMC. Res. Notes* **7**, 223 (2014).

180. P. Marrero, K. K. Abu-Amero, J. M. Larruga, V. M. Cabrera, Carriers of human mitochondrial DNA macrohaplogroup M colonized India from southeastern Asia. *BMC Evol. Biol.* **16**, 246 (2016).

181. M.-S. Peng, H. H. Quang, K. P. Dang, A. V. Trieu, H.-W. Wang, Y.-G. Yao, Q.-P. Kong, Y.-P. Zhang, Tracing the Austronesian footprint in Mainland Southeast Asia: A perspective from mitochondrial DNA. *Mol. Biol. Evol.* **27**, 2417–2430 (2010).

182. D. Pierron, M. Heiske, H. Razafindrazaka, I. Rakoto, N. Rabetokotany, B. Ravololomanga, L. M.-A. Rakotozafy, M. M. Rakotomalala, M. Razafiarivony, B. Rasoarifetra, M. A. Raharijesy, L. Razafindralambo, Ramilisonina, F. Fanony, S. Lejamble, O. Thomas, A. M. Abdallah, C. Rocher, A. Arachiche, L. Tonaso, V. Pereda-Loth, S. Schiavinato, N. Brucato, F.-X. Ricaut, P. Kusuma, H. Sudoyo, S. Ni, A. Boland, J.-F. Deleuze, P. Beaujard, P. Grange, S. Adelaar, M. Stoneking, J.-A. Rakotoarisoa, C. Radimilahy, T. Letellier, Genomic landscape of human diversity across Madagascar. *Proc. Natl. Acad. Sci. U.S.A.* **114**, E6498–E6506 (2017).

183. M. J. Pierson, R. Martinez-Arias, B. R. Holland, N. J. Gemmell, M. E. Hurles, D. Penny, Deciphering past human population movements in Oceania: Provably optimal trees of 127 mtDNA genomes. *Mol. Biol. Evol.* **23**, 1966–1975 (2006).

184. D. M. Reiff, R. Spathis, C. W. Chan, M. G. Vilar, K. Sankaranarayanan, D. Lynch, E. Ehrlich, S. Kerath, R. Chowdhury, L. Robinowitz, J. Koji Lum, R. M. Garruto, Inherited and somatic mitochondrial DNA mutations in Guam amyotrophic lateral sclerosis and parkinsonism-dementia. *Neurol. Sci.* **32**, 883–892 (2011).

185. M. Summerer, J. Horst, G. Erhart, H. Weißensteiner, S. Schönherr, D. Pacher, L. Forer, D. Horst, A. Manhart, B. Horst, T. Sanguansermsri, A. Kloss-Brandstätter, Large-scale mitochondrial DNA analysis in Southeast Asia reveals evolutionary effects of cultural isolation in the multi-ethnic population of Myanmar. *BMC Evol. Biol.* **14**, 17 (2014).

186. K. A. Tabbada, J. Trejaut, J.-H. Loo, Y.-M. Chen, M. Lin, M. Mirazón-Lahr, T. Kivisild, M. C. A. De Ungria, Philippine mitochondrial DNA diversity: A populated viaduct between Taiwan and Indonesia? *Mol. Biol. Evol.* **27**, 21–31 (2010).

187. L.-P. Wong, R. T.-H. Ong, W.-T. Poh, X. Liu, P. Chen, R. Li, K. K.-Y. Lam, N. E. Pillai, K.-S. Sim, H. Xu, N.-L. Sim, S.-M. Teo, J.-N. Foo, L. W.-L. Tan, Y. Lim, S.-H. Koo, L. S.-H. Gan, C.-Y. Cheng, S. Wee, E. P.-H. Yap, P. C. Ng, W.-Y. Lim, R. Soong, M. R. Wenk, T. Aung, T.-Y. Wong, C.-C. Khor, P. Little, K.-S. Chia, Y.-Y. Teo, Deep whole-genome sequencing of 100 Southeast Asian Malays. *Am. J. Hum. Genet.* **92**, 52–66 (2013).

188. Z. G. Yuan, Y. G. Yao, Z. X. Ma, Q. P. Pang, Y. R. Jie, J. Ma, Y. P. Zhang, Mitochondrial DNA sequence variations of Zhuang ethnic group in Guangxi. *Yi Chuan Xue Bao* **28**, 95–102 (2001).

189. X. Zhang, X. Qi, Z. Yang, B. Serey, T. Sovannary, L. Bunnath, H. Seang Aun, H. Samnom, H. Zhang, Q. Lin, M. van Oven, H. Shi, B. Su, Analysis of mitochondrial genome diversity identifies new and ancient maternal lineages in Cambodian aborigines. *Nat. Commun.* **4**, 2599 (2013).
